# Vitroprocines, new antibiotics against *Acinetobacter baumannii*, discovered from marine *Vibrio* sp. QWI-06 using mass-spectrometry-based metabolomics approach

**DOI:** 10.1038/srep12856

**Published:** 2015-08-04

**Authors:** Chih-Chuang Liaw, Pei-Chin Chen, Chao-Jen Shih, Sung-Pin Tseng, Ying-Mi Lai, Chi-Hsin Hsu, Pieter C. Dorrestein, Yu-Liang Yang

**Affiliations:** 1 Department of Marine Biotechnology and Resources, National Sun Yat-sen University, Kaohsiung 80424, Taiwan; 2 Doctoral Degree Program in Marine Biotechnology, National Sun Yat-sen University, Kaohsiung 80424, Taiwan; 3 Graduate Institute of Natural Products, Kaohsiung Medical University, Kaohsiung 807, Taiwan; 4 Agricultural Biotechnology Research Center, Academia Sinica, Taipei 11529, Taiwan; 5 Department of Medical Laboratory Sciences and Biotechnology, Kaohsiung Medical University, Kaohsiung 807, Taiwan; 6 Skaggs School of Pharmacy and Pharmaceutical Sciences, University of California, San Diego, La Jolla, CA 92093-0636, United States

## Abstract

A robust and convenient research strategy integrating state-of-the-art analytical techniques is needed to efficiently discover novel compounds from marine microbial resources. In this study, we identified a series of amino-polyketide derivatives, vitroprocines A-J, from the marine bacterium *Vibrio* sp. QWI-06 by an integrated approach using imaging mass spectroscopy and molecular networking, as well as conventional bioactivity-guided fractionation and isolation. The structure-activity relationship of vitroprocines against *Acinetobacter baumannii* is proposed. In addition, feeding experiments with ^13^C-labeled precursors indicated that a pyridoxal 5′-phosphate-dependent mechanism is involved in the biosynthesis of vitroprocines. Elucidation of amino-polyketide derivatives from a species of marine bacteria for the first time demonstrates the potential of this integrated metabolomics approach to uncover marine bacterial biodiversity.

Oceans cover 70% of the Earth’s surface and harbor most of the planet’s biodiversity. The abundant biodiversity of marine organisms, such as sponges, cnidaria and microorganisms are regarded as rich sources of novel chemical scaffolds of natural products, which has led to the discovery of several marine-based drugs^1^,^2^ such as the anticancer cytosine arabinoside, the FDA-approved peptidial ω-conotoxin MVIIA for releasing chronic pain, and ecteinascidin-743 (ET-743) to treat patients with advanced soft tissue sarcoma^3^,^4^. However, given the vast area of the world’s oceans from which bioactive natural marine products can potentially be gleaned, various dereplication strategies for bioactive agent discovery have been proposed, such as bioassay screening, genomic analysis, and even state-of-the-art analytical techniques such as NMR, imaging mass spectrometry (IMS) and LC-MS/MS, in which mass spectrometry-based metabolomics becomes critical and effective due to reduced analysis time and the necessity for less sample material^5^,^6^.

To explore the bioactive secondary metabolites of marine-derived microbes, 265 marine-derived microbes were uncovered from the sediments of the intertidal zone and deep-sea in the Taiwan Strait. The antibacterial activity of these isolated bacteria were screened against nine indicator strains, Gram positive *Bacillus cereus, Staphylococcus aureus*, Gram negative *Klebsiella pneumonia, Salmonella typhimurium, Pseudomonas aeruginosa, Vibrio harveyi, Escherichia coli*, and *Acinetobacter baumannii*, and one fungus strain *Candida albicans*. Nineteen of the marine-derived bacteria exhibited significant anti-microbial activities. Among them, one strain, QWI-06, showed significant inhibitory activity against *A. baumannii*, a major pathogen of nosocomial infections in Taiwan (Supplementary Fig. S1). The 16S rRNA sequence of the strain QWI-06 was close to the type strain *Vibrio proteolyticu*s ATCC 15338 with 99.4% similarity (Supplementary Fig. S2); however, to our knowledge, this type of bioactivity has not been reported for metabolites obtained from the genus *Vibrio*. We further confirmed that *V. proteolyticu*s ATCC 15338 and its crude extract were inactive against *A. baumannii* in both antagonistic and anti-bacterial assays. A comparative metabolomics analysis of *Vibrio* sp. QWI-06 and *V. proteolyticu*s ATCC 15338, using imaging mass spectrometry (IMS) and ultra-performance liquid chromatography–high-resolution electrospray ionization tandem mass spectrometry (UPLC-HR-ESIMS/MS), identified a series of amino-polyketide derivatives, named vitroprocines, from *Vibrio* sp. QWI-06, which contributed to the inhibitory effects against *A. baumannii*. Detailed structural elucidation, a possible biosynthetic pathway, and the structure-activity relationship of the vitroprocines are reported herein.

## Results

### Mass-spectrometry-based metabolomics analysis and bioactivity-guided isolation

**Figure 1:**
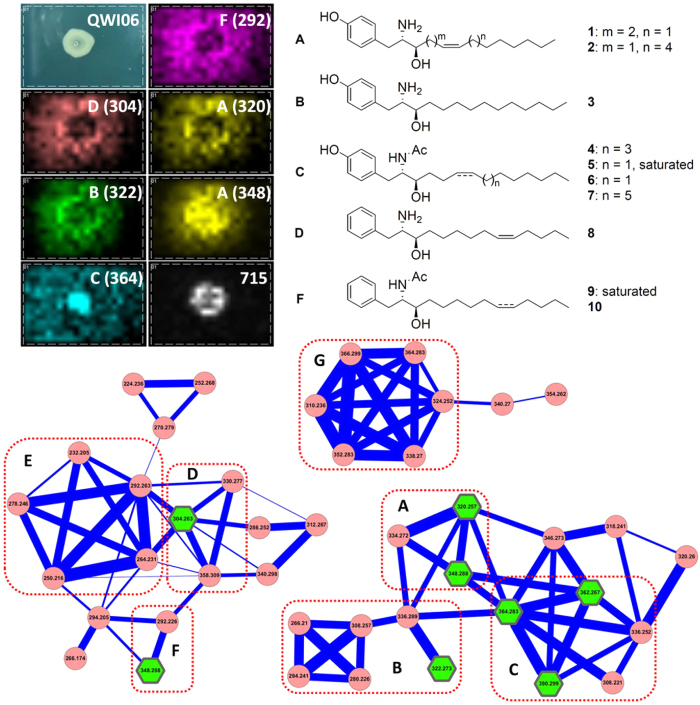
Imaging mass spectrometry and molecular networking analysis of *Vibrio* sp. QWI-06 and structures of vitroprocines A-J (**1**–**10**). Three clusters in molecular networking of *Vibrio* sp. QWI-06 extract consist of seven subgroups (**A**–**G**). Six nodes were observed in the imaging mass spectrometry of *Vibrio* sp. QWI-06 colony grown on marine agar medium including subgroup A: m/z 320 (**1**) and 348 (**2**); subgroup B: m/z 322 (**3**); subgroup C: m/z 364 (**5**); subgroup D: m/z 304 (**8**); and subgroup F: m/z 292. m/z 715 is polyglutamate analogue commonly found in bacteria. Eight nodes (shown is green hexagon) were isolated in this study: **1**–**6**, **8** and **10**.

Imaging mass spectrometry is a label-free imaging technique used to collect metabolic information from intact cells without further organic solvent extraction and fractionation^7^,^8^,^9^,^10^,^11^,^12^,^13^. The IMS data from the *Vibrio* sp. QWI-06 and *V. proteolyticu*s ATCC 15338 colonies were significantly different, ranging from m/z 200 to 400 (Fig. 1 and Supplementary Fig. S3). The spatial distribution of most of the ions in this range indicates that they are secreted metabolites and the tandem mass spectra collected directly from the IMS samples suggest they are structurally similar. Based on bioactivity-guided fractionation by HPLC using a MeOH-H_2_O gradient elution system, we found that the active fractions of the EtOAc extract of *Vibrio* sp. QWI-06, which potently inhibited *A. baumannii*, consisted of the same metabolites as revealed in the IMS analysis. Therefore, the EtOAc extract was further subjected to UPLC-HR-ESIMS/MS for molecular networking analysis and dereplication. Molecular networking is a platform for providing an overview of the molecular features in mass-spectrometry-based metabolomics by comparing fragmentation patterns to identify chemical relationships^14^,^15^,^16^,^17^,^18^. This comparison is based upon the similarity cosine score of the tandem mass spectra and the visualization of those relationships in a two-dimensional network in Cytoscape (Version 3.2.0)^19^, where each node represents a parent mass and each edge represents the fragmentation similarity. The thickness of the edges corresponds to the significance of the similarity with thicker lines corresponding to higher degrees of similarity. Three clusters, composed of 43 chemical species (nodes), including those revealed in IMS of the bacterial colony and LC-ESIMS of the active fractions, were generated by molecular networking analysis (Fig. 1). The interpretation of tandem mass fragmentations suggested that 31 of the metabolites (nodes) can be differentiated into seven subgroups. None of them was dereplicated from either the MarinLit or AntiMarin databases, which implies that this is the first time that these metabolites have been identified from marine microbial sources. In view of these results, we decided to isolate these metabolites for structure and bioactivity evaluation. The EtOAc extract was then partitioned with MeOH and *n*-hexane, and the MeOH-soluble fraction was repeatedly chromatographed to afford ten new compounds, vitroprocines A-J (**1**–**10**) (Fig. 1). The structures of the isolated metabolites were elucidated using NMR and high resolution mass spectrometry and are discussed in the next section. Eight vitroprocines were revealed in the molecular networking analysis: **1** and **2** were in subgroup A, **3** was in subgroup B, **4**–**6** were in subgroup C, **8** was in subgroup D, and **9** was in subgroup F. None of the analogues in subgroups E and G were isolated in this study due to the limited amount of materials, but their structures were proposed based on the interpretation of molecular formulas and tandem mass fragmentations deduced from high resolution mass spectra (Supplementary Table S3). The structures of subgroup E were close to those of subgroup D, and they were proposed to be phenylalanine-polyketide derivatives with a saturated aliphatic chain. On the other hand, the structures of subgroup G were proposed to be tyrosine-polyketide derivatives like subgroups A-C. They were not in the same cluster as subgroups A-C because one more hydroxyl group was proposed on the aliphatic chain of subgroup G. The structures of the rest of the analogues explored by molecular networking analysis are summarized in the Supplementary Information (Tables S2–S3, Figs S4–S12).

### Structure elucidation of vitroprocines A-J (1–10)

**Figure 2:**
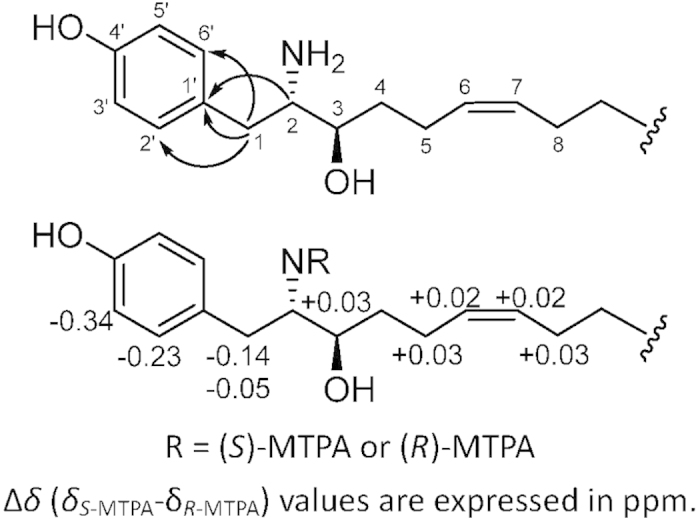
Key HMBC correlations and stereochemical analysis of vitroprocine A (**1**).

**Figure 3:**
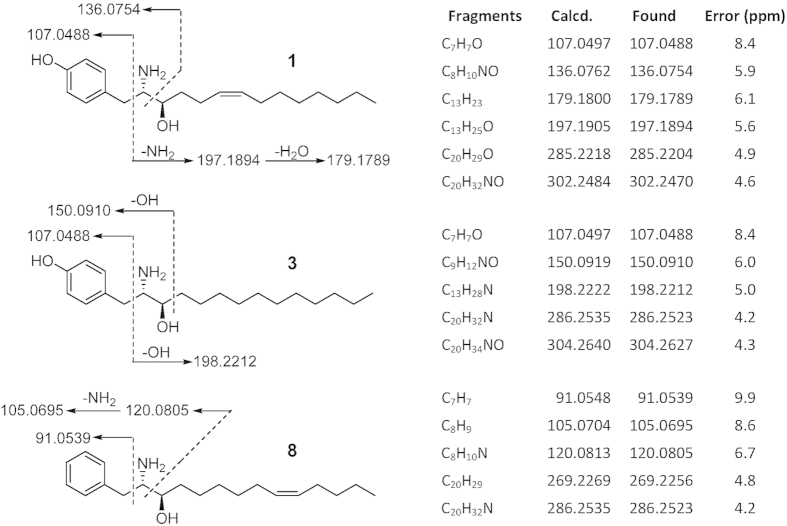
HR-ESIMS/MS fragmentations of compounds **1**, **3**, and **8.**

In subgroup A, compounds **1** and **2** were isolated. Compound **1** was obtained as a white amorphous solid by RP-HPLC. The HR-ESIMS data of **1** showed a base peak at m/z 320.2570 [M + H]^+^, consistent with a molecular formula of C_20_H_33_NO_2_ and an index of hydrogen deficiency (IHD) of 5. The UV absorption at 203, 224, and 278 nm and IR absorption at 3305 cm^−1^ suggested the presence of a phenyl group in **1**. The ^1^H NMR and ^1^H-^1^H COSY spectra of **1** exhibited two partial structures, a para-substituted phenyl group with a pair of signals [δ_H_ 7.10 (d, J = 8.5 Hz, 2H) and 6.78 (d, J = 8.5 Hz, 2H)], and an aliphatic long chain with two olefinic protons [δ_H_ 5.38 (m, 2H)], two methines [δ_H_ 3.74 (dt, J = 9.0, 3.2 Hz, 1H) and 3.38 (ddd, J = 9.0, 5.5, 3.2 Hz, 1H)], nine methylenes [one benzylic CH_2_: δ_H_ 2.92 (dd, J = 14.4, 5.5 Hz, 1H) and 2.72 (dd, J = 14.4, 9.0 Hz, 1H); two allylic CH_2_: 2.07 (m, 4 H); six aliphatic CH_2_: 1.25~1.60 (m, 12H)], and one terminal methyl [δ_H_ 0.90 (t, J = 7.0 Hz, 3H)] (Supplementary Table S1). The ^13^C and DEPT experiments of **1** showed the presence of 20 resonances, including six aromatic carbons, two olefinic carbons, one oxymethine carbon, one aminomethine carbon, nine methylene carbons, and one terminal methyl carbon (Supplementary Table S1). The connection of the two partial structures was established by the HMBC correlations between δ_H_ 2.92 and 2.74 (H_2_-1)/δ_C_ 128.1 (Ar-C-1′) and 131.4 (Ar-C-2′and 6′), and δ_H_ 3.38 (H-2)/δ_C_ 128.1 (Ar-C-1′) (Fig. 2). The placement of the para-substituted phenol, amine, and hydroxyl groups was also confirmed by close examination of the fragmentation of **1** (Fig. 3) in the HR-ESIMS/MS spectrum for the significant signals at m/z 197.1894 (C_13_H_25_O), 179.1789 (C_13_H_23_), 136.0754 (C_8_H_10_NO), and 107.0488 (C_7_H_7_O), which further allowed the placement of a para-substituted phenol moiety linked with C-1. The planar structure of **1** is similar to tyroscherin, which is a tyrosine-polyketide derivative^20^,^21^,^22^,^23^,^24^.

The double bond position in the aliphatic chain of **1** was determined by using electron impact mass spectrometry (EIMS) on the dimethyl disulfide derivative of **1**^25^. The EIMS fragment at m/z 254 indicated the double bond should be located at Δ_6,7_ (Supplementary Fig. S21). The ^13^C NMR chemical shifts at δ_C_ 28.4 and 28.1 of the allylic carbons (C-5 and C-8) suggested the geometric conformation of the double bond is *Z* form based on the rule that the chemical shifts of allylic carbons of linear olefins of *Z*-isomers resonate at higher field (about 5 ppm) than those of *E*-isomers^26^.

The stereochemistry of **1** was elucidated by (*S*)- and (*R*)-Mosher ester derivatives of **1** (Fig. 2 and Supplementary Fig. S20)^27^. The correlations between H_2_-1, H-2, H-3, and H-5 of the *N*-MTPA derivatives were established on the basis of their COSY spectrum. The Δδ_H(*S*-*R*)_ values indicated that the absolute configuration at C-2 was *S*. Furthermore, the close analysis of the ^1^H-^1^H coupling constants of H-2 and H-3 by the Newman projection model indicated that the vicinal proton-proton coupling constant ^3^J_H2-H3_ required a *gauche* form, which was consistent with the revised structure of tyroscherin, suggesting that the chiral center at C-3 is *R*^21^,^22^,^23^. Thus, the structure of **1** was determined to be 4-((2*S*,3*R*,*Z*)-2-amino-3-hydroxytetradec-6-en-1-yl) phenol and named vitroprocine A.

Compound **2** was obtained as a white amorphous solid by RP-HPLC and has the molecular formula C_22_H_37_NO_2_ as deduced from HR-ESIMS analysis (m/z 348.2882 [M + H]^+^, IHD 5). The ^1^H and ^13^C NMR spectra of **2** (Supplementary Table S1) were identical to those of vitroprocine A (**1**). The HR-ESIMS/MS fragmentations of **2** showed a similar fragmentation pattern to that of **1** (Supplementary Fig. S6). The significant peaks at m/z 225.2208 (C_13_H_29_O), 207.2104 (C_15_H_27_), 136.0754 (C_8_H_10_NO), and 107.0488 (C_7_H_7_O) allowed the placement of a para-substituted phenol moiety linked with C-1 and the aliphatic chain of **2** has two more methylenes than that of **1**. Moreover, the double bond was determined at Δ_5,6_ in the aliphatic chain according to the EIMS fragment of its dimethyl disulfide derivative (m/z 240) (Supplementary Fig. S29). The ^13^C NMR chemical shifts of the allylic methylene carbons (C-4 and C-7 at δ_C_ 28.3) suggested that the geometric conformation of the double bond is *Z* form. Thus, the structure of **2** was determined as shown (Fig. 1) and named vitroprocine B.

In subgroup B, compound **3** was obtained as a white amorphous solid by RP-HPLC. The HR-ESIMS data of compound **3** showed a base peak at m/z 322.2726 [M + H]^+^, consistent with a molecular formula of C_20_H_35_NO_2_, and the IHD was deduced to be 4. The UV absorption at 204, 225 and 279 nm together with the IR absorption at 3383 cm^−1^ suggested the presence of a phenyl group in **3**. The ^1^H and ^13^C NMR spectra of **3** were similar to that of **1**, except for the absence of the olefinic signals (Supplementary Table S1). The HR-ESIMS/MS fragmentation pattern of **3** (Fig. 3) at m/z 304.2627 (C_20_H_34_NO), 286.2523 (C_20_H_32_N), 198.2212 (C_13_H_28_N), 150.0910 (C_9_H_12_NO), and 107.0488 (C_7_H_7_O) confirmed the structure of compound **3** to be a derivative of **1**, devoid of a double bond on the aliphatic chain. Thus, **3** was determined as shown (Fig. 1) and named vitroprocine C. Compound **3** was clustered in subgroup B suggesting that it is a tyrosine-polyketide derivative with a saturated aliphatic chain.

Compounds **4**, **5** and **6**, belonging to subgroup C, were isolated. Compound **4** was obtained as a white amorphous solid by RP-HPLC and has a molecular formula of C_24_H_39_NO_3_ based on the HR-ESIMS analysis (m/z 390.2986 [M + H]^+^, IHD 6). The UV absorption at 204, 224 and 278 nm together with the IR absorption at 3341 cm^−1^ revealed the presence of a phenyl group in **4**. In addition, the IR absorption at 1543 cm^−1^ suggested **4** has an acetyl group. The ^1^H and ^13^C NMR spectra of **4** are very similar to those of **1**, except for two downfield methines [δ_H_ 3.96 (m, 1H)/δ_C_ 57.4, and δ_H_ 3.50 (m, 1H)/δ_C_ 74.5], two methylenes plus one acetyl group [δ_H_ 1.82 (s, 3H)/δ_C_ 172.8, 22.7 (C-18)], suggesting that **4** is an *N*-acetylized derivative of **1**^28^,^29^. The location of the acetyl group was further confirmed by the key HMBC correlations [δ_H_ 1.82 (H-18) and δ_H_ 3.96 (H-2) correlated with δ_C_ 172.8 (C-17)]. The double bond position in the aliphatic chain of **4** was determined by the dimethyl disulfide derivative method. The fundamental EIMS fragment signal at m/z 254 indicated that a double bond should be located at Δ_6,7_ (Supplementary Fig. S44). The ^13^C NMR chemical shifts of the allylic methylene carbons C-5 and C-8 at δ_C_ 28.3 suggested the geometric conformation of the double bond is *Z* form. Therefore, the structure of **4** was determined as shown and named vitroprocine D.

Compounds **5** and **6** were obtained as white amorphous solids from the same fraction by RP-HPLC and have molecular formulas of C_22_H_37_NO_3_ and C_22_H_35_NO_3_ based on HR-ESIMS signals at m/z 364.2831 [M + H]^+^ and m/z 362.2675 [M + H]^+^, respectively. IHDs of 5 and 6, respectively, were deduced, implying that **6** has one more double bond than **5**. Moreover, the ^1^H and ^13^C NMR spectra of **5** and **6** are identical with those of **4**, except for the absence of the olefinic protons at δ_H_ 5.35 (m, 2H) and carbons at δ_C_ 131.0 and 130.9 in **5**. Combining the mass spectral data of both compounds, **5** was identified as an *N*-acetylized derivative of **3**, whereas **6** was an *N*-acetylized derivative of **1**. The geometric conformation and location of the double bond in **6** was determined to be *Z*-form and at Δ_6,7_, based on ^13^C NMR chemical shifts of the allylic methylene carbons [δ_C_ 28.4 (C-5) and 28.3 (C-8)], and EIMS fragmentation from its dimethyl disulfide derivative (Supplementary Fig. S53). Thus, the structures of **5** and **6** were fully established and named vitroprocines E and F, respectively.

Compound **7** was obtained as a white amorphous solid by RP-HPLC and has a molecular formula of C_26_H_43_NO_3_ as deduced from the HR-ESIMS analysis (m/z 418.3399 [M + H]^+^). The UV, IR, and NMR spectra of **7** were similar to those of **4** and **6**, which indicated the presence of a para-substituted phenyl group [δ_H_ 7.01 (d, J = 8.4 Hz, 2H) and 6.67 (d, J = 8.4 Hz, 2H)], attached to an unsaturated aliphatic chain with one hydroxyl group and one acetylamine moiety. The double bond position on the aliphatic chain was determined at Δ_6,7_ according to the EIMS fragmentation of its dimethyl disulfide derivative (Supplementary Fig. S58), and the ^13^C NMR chemical shifts (δ_C_ 28.3, C-5 and 8) suggested a *Z* geometric conformation of the double bond. Thus, the structure of **7** was determined as shown (Fig. 1) and named vitroprocine G.

In subgroups D–F, two compounds were isolated: compound **8** belongs to subgroup D and compound **9** to subgroup F. They are different from the cluster of subgroups A-C because of the absence of an important fragment of C_7_H_7_O (Calcd. mass: 107.0497Da) in the tandem mass spectra, which corresponded to the para-substituted phenyl group. Compound **8** was obtained as a white amorphous solid by RP-HPLC. The HR-ESIMS data of **8** showed a base peak at m/z 304.2623 [M + H]^+^, consistent with a molecular formula of C_20_H_33_NO and the IHD was 5. The UV absorption at 208 and 258 nm and the IR absorption at 3411 cm^−1^ suggested that **8** has a benzyl group rather than a phenyl group as indicated by the subgrouping in the molecular networking. ^1^H NMR spectrum of **8** exhibited signals of a monosubstituted benzyl group [δ_H_ 7.35 (t, J = 7.5 Hz, 2H) and 7.28 (d, J = 6.8 Hz, 3H)] and two olefinic protons [δ_H_ 5.40 and 5.36 (m, each 1H)]. Comparison with the NMR data of **1** and **8** indicated that both possessed a similar skeleton, except that the para-substituted phenyl group in **1** was replaced by a benzyl group in **8**. The planar structure of **8** was further established by the close examination of the HR-ESIMS/MS fragmentations. The significant signals at m/z 120.0805 (C_8_H_10_N), 105.0695 (C_8_H_9_), and 91.0539 (C_7_H_7_) allowed the placement of the benzyl moiety linked with C-1 (Fig. 3). Furthermore, the dimethyl disulfide derivative of **8** was subjected to EIMS analysis to determine the location of the double bond at Δ_9,10_ on the aliphatic chain based on a fragment signal at m/z 279 (Supplementary Fig. S66). Besides, the ^13^C NMR chemical shifts of the allylic methylene carbons (C-8 and C-11, at δ_C_ 28.4 and 28.2) suggested a *Z* geometric conformation of the double bond. Thus, the structure of **8** was determined as shown (Fig. 1) and named vitroprocine H, a phenylalanine-polyketide derivative.

Compounds **9** and **10** were obtained as white amorphous solids from the same fraction by RP-HPLC and have molecular formulas of C_22_H_37_NO_2_ and C_22_H_35_NO_2_ based on HR-ESIMS (**9**, m/z 348.2903 [M + H]^+^; **10**, 346.2726 [M + H]^+^). The IHD deduced from molecular formula suggest both compounds have only one double bond difference. The UV absorption at 210 and 260 nm for **9**, 205 and 259 nm for **10** suggested both compounds have a benzyl group like **8**. However, the IR absorption at 1552 cm^−1^ for **9** and 1556 cm^−1^ for **10** indicated both compounds have an acetyl group, which is absent in **8**. Compound **9** contains a saturated aliphatic chain, whereas the NMR signals for C-6 [δ_H_ 5.37 (m, 1H; H-6); δ_C_ 131.3] and C-7 [δ_H_ 5.37 (m, 1H; H-7); δ_C_ 130.7] of compound **10** indicated a double bond on the aliphatic chain. The double bond position of **10** was determined at Δ_9,10_ due to the interpretation of EIMS fragmentation of its dimethyl disulfide derivative (Supplementary Fig. S75). Based on the aforementioned data, the structures of **9** and **10** were established and named vitroprocines I and J, respectively.

### Biosynthesis of Vitroprocines

**Figure 4:**
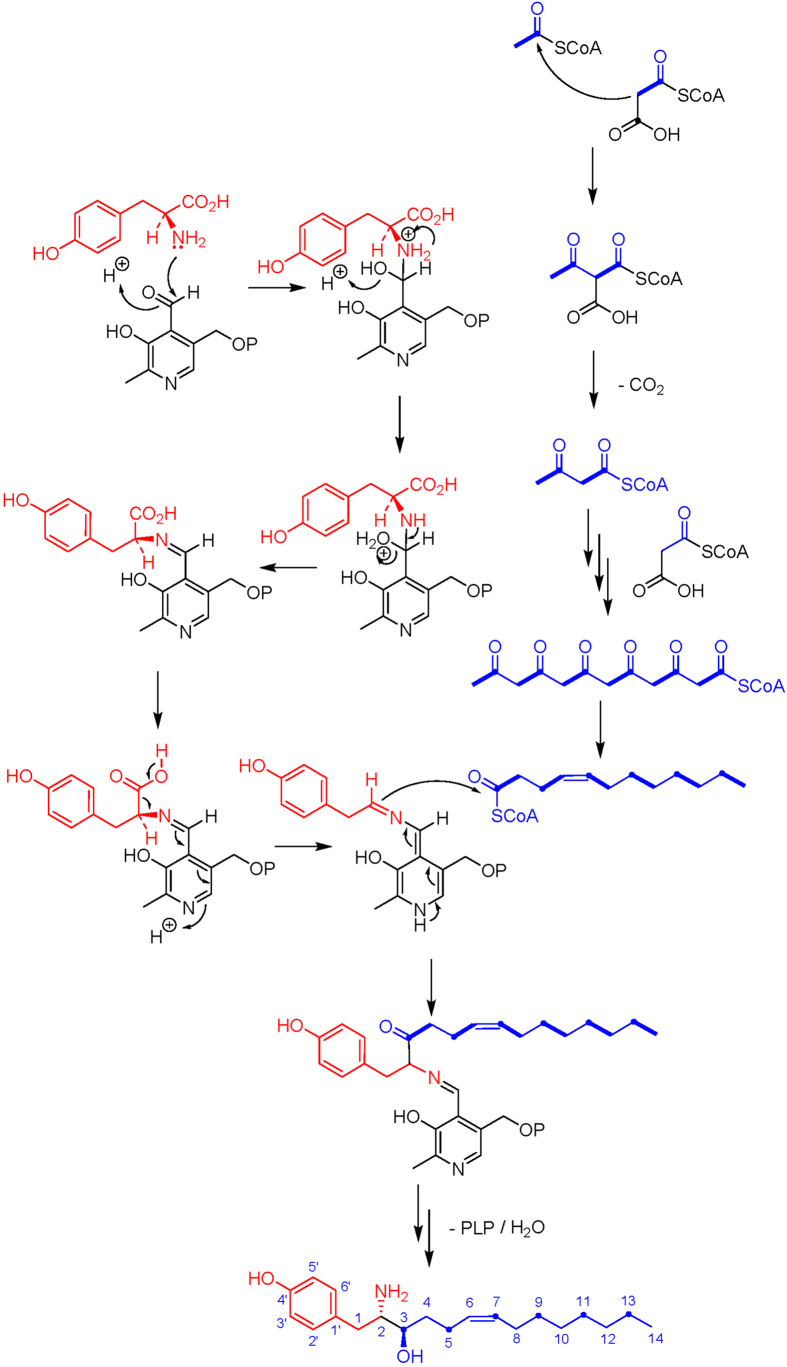
Plausible biosynthetic pathway of vitroprocines deduced from isotope labelling experiments.

From the structural perspective, the vitroprocines are proposed to be amino-polyketide derivatives. To confirm this, L- and D-tyrosines, together with L-phenylalanine, were added individually into a culture medium to evaluate the amino acid precursors of vitroprocines. The quantitative analysis deduced from the triple quadrupole LC-MS system demonstrated that the feeding of L-tyrosine and L-phenylalanine can significantly increase the production of tyrosine-polyketide derivatives (such as **1** and **3**) and phenylalanine-polyketide derivatives (such as **8**), respectively (Supplementary Fig. S76). On the other hand, the feeding of D-tyrosine obviously did not influence the production of vitroprocines. In addition, 1-^13^C-acetate and ^13^C_9_-tyrosine were used to evaluate the biosynthetic pathway. We found that the C-3 position of vitroprocines biosynthetically originated from an acetate rather than a tyrosine (Supplementary Figs S77 and S78), which suggested a pyridoxal 5′-phosphate (PLP)-dependent mechanism for the polyketide chain release involved in the biosynthesis of vitroprocines. A plausible biosynthetic pathway of vitroprocines is proposed in Fig. 4.

### Anti-bacterial activity and structure-activity relationship analysis

Vitroprocines were evaluated for their anti-bacterial effects against *A. baumannii*. Among them, **1**, **2**, and **3** showed inhibitory effects against *A. baumannii* (MIC ≤ 8, 32, and 16 μg/mL, respectively) and the MIC values of **1** and **3** are competitive with the clinical drugs ceftazidime and ticarcillin. The MIC values of the other vitroprocines are >32 and ≤64 μg/mL. The structure-activity relationship (SAR) of the vitroprocines is summarized into four points as follows: (1) the para-substituted phenyl group is important for their anti-bacterial activity, for example, the inhibitory effects of **1** and **3** were more potent than **8**; (2) the shorter the aliphatic chain the better the inhibitory effect, for example, **1** and **3** are more active than compound **2**; (3) the presence of the double bond on aliphatic chain will enhance the inhibitory effect, for example, **1** is more active than **3**; (4) the free amine is essential for the inhibitory effect because all *N*-acetylized derivatives are inactive against *A. baumannii*.

## Discussion

In this study, 43 chemical species (nodes) were generated as three clusters, composed of seven subgroups by molecular networking analysis. Among them, the structures of 31 metabolites were proposed based on the interpretation of tandem mass fragmentations. Ten vitroprocines, including three active ones, were isolated following the bioactivity-guided fractionation and isolation against *A. baumannii*. The results demonstrate that molecular networking analysis can not only be used for dereplication and new metabolite discovery, but also for the prediction of active metabolites. The SAR of the isolated vitroprocines suggests that metabolites A2, B1–B4 (Supplementary Table S3) are active against *A. baumannii* and the metabolites of subgroups C, D, and F are inactive. The antibiotic activity of subgroups E and G is unpredictable, since no members of subgroups E and G were isolated following bioactivity-guided fractionation and isolation. This could be due to the limitation of either trace amount or insufficient antibiotic activity.

In the biosynthetic analysis, we suggested a PLP-dependent mechanism for the polyketides chain release involved in the biosynthesis of vitroprocines. The PLP-dependent enzymes are responsible for offloading and elongating the polyketide or the fatty acid chain and introducing an amino acid into the structure, such as alanine into the sphinganine-analog mycotoxins^30^,^31^,^32^, serine into the sphingolipids^33^,^34^, or cysteate into the sulfonolipids^35^. Sphingolipids are ubiquitous and essential components in eukaryotes but occur in only a few α- and β-proteobacteria, such as genera *Pedobacter, Bacteroides, Prevotella, Porphyromonas, Fusobacterium, Sphingomonas, Sphingobacterium, Bdellovibrio, Cystobacter, Mycoplasma, Flectobacillus*, and *Acetobacter*^35^,^36^. Sulfonolipids are found in the genus *Cytophaga*^37^,^38^,^39^. Neither sphingolipids nor sulfonolipids have been reported to occur in the genus *Vibrio*. The bacterial serine palmitoyltransferases conduct the biosynthesis of sphingolipids and sulfonolipids^34^,^35^,^40^. Through genome mining of genus *Vibrio*, we found that a putative serine palmitoyltransferase gene (accession number GAD67194) exists in the whole genome sequence of *V. proteolyticus* NBRC 13287. This implies that *V. proteolyticus* has the potential to produce sphingolipids, sulfonolipids, or even vitroprocines. In the UPLC-HR-ESIMS and MS/MS analysis of *V. proteolyticus* ATCC 15338 crude extract, we indeed found a trace amount of compounds **1**, **3**, and **8**, which should be rationally involved with the presence of serine palmitoyltransferase-like enzyme in this *Vibrio* species.

In addition to the bacterial sphingolipids and sulfonolipids, a similar structure, tyroscherin, was identified from *Pseudallescheria* sp^20^,^21^,^22^,^23^. Vitroprocines share the same core structure and absolute configuration with tyroscherin, except for the aliphatic chain and the modification of the amine (the *N*-methyl group in tyroscherin and the free amine in the vitroprocines). From a biosynthetic perspective, it is reasonable to suggest that vitroprocines and tyroscherin are derived from a similar biosynthetic pathway. Tyroscherin showed strong cytotoxicity against IGF-1-dependent cancer cells; however, the vitroprocines were non-toxic to mammalian cells. The SAR analysis of tyroscherin demonstrated that the stereochemistries of C-2 and C-3 are important for cytotoxicity^23^. Thus, we surmised that the methylation of amine is important for the cytotoxicity of tyroscherin as well.

In this study we have demonstrated an example of the efficient discovery of bioactive compounds from a marine microbe using an integrated mass-spectrometry-based metabolomics approach. This approach has the potential to expedite the discovery of novel bioactive compound scaffolds from diverse marine microbial resources.

## Methods

### Isolation and identification of *Vibrio* sp. QWI-06

*Vibrio* sp. QWI-06 was isolated from the sediment collected in Dapeng Bay National Scenic Area, Pingtung County, Taiwan. The 16S rRNA gene sequencing (Accession: KR052023) of this strain was affiliated with the genus *Vibrio* and the most closely related type strain is *Vibrio proteolyticus* ATCC 15338 (Accession: NR_118927) with 99.4% identity (Supplementary Fig. S2). A voucher specimen (Strain QWI-06) was deposited in the Department of Marine Biotechnology and Resources, National Sun Yat-sen University, Taiwan.

### Microbial imaging mass spectrometry

he bacteria were grown in Difco marine agar medium in Petri dishes (90 mm × 15 mm) for three days at 30 °C. The agar medium was kept at 0.5–1 mm thickness. The regions of interest regions were cut off and moved to a MALDI target plate (ITO glass). A 1:1 mixture of α‐cyano‐4‐hydroxycinnamic acid and 2,5‐dihydroxybenzoic acid was sprinkled on top of the culture using a 50 μm sieve. Once the sample was completely covered with matrix, it was exposed to air in a 37 °C oven for 3 hours until it is deemed dried at which point it was subjected to IMS. The IMS data were acquired in a Bruker autoflex speed MALDI-TOF/TOF system, using reflectron positive mode ion detection between m/z 120-1200, 50% laser power and 333.3 Hz laser frequency, 1000 shots per raster and 1100 μm raster resolution.

### Acquisition of LC-ESIMS data for molecular networking and quantitation analysis

For quantitation analysis, the mass data were acquired in triplicate using UPLC-ESIMS (Thermo TSQ Quantum Access MAX Triple Quadrupole system). The EtOAc crude extract of *Vibrio* sp. QWI-06 (0.1 mg/mL) was dissolved in ACN from which 5 μL was injected and separated by C18 (ACQUITY UPLC HSS T3, 1.8 μm, 2.1 × 100 mm) with the following gradient: 0–6 min at 10–100% of B (A: ACN : H_2_O = 2 : 98 plus 0.1% formic acid; B: ACN plus 0.1% formic acid), 6–8 min at 100% of B, 8–8.2 min at 100–10% of B, 8.2–10 min at 10% of B. Flow rate was set at 0.5 mL/min. The precursor ions, m/z 320.05, 322.51 and 304.26, were selected and fragmented respectively with normalized collision energy 25.0 to give the product ions, m/z 107.07, 107.07, and 286.20, which were used multiple reaction monitoring. For molecular networking analysis, the mass data were acquired in triplicate using UPLC-HR-ESIMS (Thermo Orbitrap Elite system). The sample preparation and separation conditions were the same as mentioned above. The mass data were acquired in profile mode, positive mode ion detection between m/z 100–1000 with 30000 resolution. The top five intense ions from each full mass scan were selected for collision-induced dissociation (CID) fragmentation. For CID, isolation width was 2Da and the selected ions were fragmented with normalized collision energy 30.0, activation Q 0.250, activation time 10.0 and 15000 resolution. The mass data (.RAW files) from Xcalibur were converted to mzXML file format and subjected to GnPS (https://gnps.ucsd.edu/ProteoSAFe/static/gnps-splash.jsp) to generate molecular networking and the data were visualized in Cytoscape.

### Cultivation and isolation of vitroprocines

*Vibrio* sp. QWI-06 was grown in Difco marine agar medium on Petri dishes (90 mm × 15 mm) at 30 °C. The three-day cultures of *Vibrio* sp. QWI-06, total 2502 plates, were cut into small pieces and further soaked in EtOAc for one day at room temperature. This procedure was repeated for twice. The EtOAc extract was evaporated under reduced pressure to yield a brown syrup (5.10 g), which was further partitioned with MeOH and *n*-hexane to give the MeOH (4.44 g) and *n*-hexane layers (0.37 g). The MeOH layer was separated into 15 fractions by column chromatography (Sephadex LH-20) eluted by pure MeOH. Fr. 4, an active fraction against *A. baumannii* was separated by silica gel column chromatography eluted with a gradient system of *n*-hexane, CHCl_3_, and MeOH (*n*-hexane/CHCl_3_/MeOH = 100/0/0 to pure MeOH) to give 30 sub-fractions. Sub-fractions 17 and 18 were further purified by RP-HPLC (Discovery C18, 250 × 10 mm, 2.0 mL/min, MeOH/H_2_O = 85/15) to give **9** (0.7 mg, t_*R*_ 40.3 min) and **10** (0.8 mg, t_*R*_ 32.5 min). Sub-fractions 22 and 23 were further purified by RP-HPLC (Discovery C18, 250 × 10 mm, 3.0 mL/min, MeCN/H_2_O = 65/35) to give **4** (1.8 mg, t_*R*_ 23.8 min). Sub-fraction 24 was purified by silica gel column chromatography eluted with a gradient system of CH_2_Cl_2_ and MeOH (CH_2_Cl_2_/MeOH = 40/10 to 15/1) and then purified by RP-HPLC (Discovery C18, 250 × 10 mm, 3.0 mL/min, MeOH/H_2_O = 80/20) to give **5** (0.7 mg, t_*R*_ 24.9 min), **6** (1.0 mg, t_*R*_ 16.5 min), and **7** (0.7 mg, t_*R*_ 54.5 min). Sub-fraction 30 with anti-bacterial effect against *A. baumannii* was further purified by RP-HPLC (Discovery C18, 250 × 10 mm, 2.0 mL/min, MeOH/H_2_O = 65/35) to give **1** (9.0 mg, t_*R*_ 10.7 min), **3** (9.7 mg, t_*R*_ 19.6 min), **8** (0.8 mg, t_*R*_ 22.6 min), and **2** (2.3 mg, t_*R*_ 25.0 min).

Vitroprocine A (**1**) C_20_H_33_NO_2_; white amorphous solid; [α]_D_ = –26.3 (*c* *=* 0.750, MeOH); UV/Vis: λ_max_ 203, 224, 278 nm; IR (Neat): 3305 cm^−1^; ^1^H NMR (500 MHz, CD_3_OD) and ^13^C NMR (125 MHz, CD_3_OD) is shown in Supplementary Table S1; HR-ESIMS (m/z): [M + H]^+^ calcd. for C_20_H_34_NO_2_, 320.2590; found, 320.2570.

Vitroprocine B (**2**) C_22_H_37_NO_2_; white amorphous solid; [α]_D_ = –49.7 (*c* = 0.430, MeOH); UV/Vis: λ_max_ 204, 225, 279 nm; IR (Neat): 3339 cm^−1^; ^1^H NMR (500 MHz, CD_3_OD) and ^13^C NMR (125 MHz, CD_3_OD) is shown in Supplementary Table S1; HR-ESIMS (m/z): [M + H]^+^ calcd. for C_22_H_38_NO_2_, 348.2903; found, 348.2882.

Vitroprocine C (**3**) C_20_H_35_NO_2_; white amorphous solid; [α]_D_ = –29.3 (*c* = 0.325, MeOH); UV/Vis: λ_max_ 204, 225, 279 nm; IR (Neat): 3383 cm^−1^; ^1^H NMR (500 MHz, CD_3_OD) and ^13^C NMR (125 MHz, CD_3_OD) is shown in Supplementary Table S1; HR-ESIMS (m/z): [M + H]^+^ calcd. for C_20_H_36_NO_2_, 322.2746; found, 322.2726.

Vitroprocine D (**4**) C_24_H_39_NO_3_; white amorphous solid; UV/Vis: λ_max_ 204, 224, 278 nm; IR (Neat): 3341 cm^−1^; ^1^H NMR (400 MHz, CD_3_OD) δ_H_ 7.01 (d, J = 8.4 Hz, 2H; H-2′ and H-6′), 6.67 (d, J = 8.4 Hz, 2H; H-3′ and H-5′), 5.35 (m, 2H; H-6 and H-7), 3.96 (m, 1H; H-2), 3.50 (m, 1H; H-3), 2.91 (dd, J = 14.0, 4.0 Hz, 1H; H-1), 2.53 (dd, J = 14.0, 10.4 Hz, 1H; H-1), 2.03 (m, 4H; H-5 and H-8), 1.82 (s, 3H; 18-CH_3_), 1.25~1.60 (m; aliphatic CH_2_), 0.90 (t, J = 6.7 Hz, 3H; 16-CH_3_); ^13^C NMR (100 MHz, CD_3_OD) δ_C_ 172.8 (C-17), 156.8 (C-4′), 131.3 (C-2′ and C-6′), 131.1 (C-1′), 131.0 (C-6), 130.9 (C-7), 116.1 (C-3′ and C-5′), 74.5 (C-3), 57.4 (C-2), 35.9 (C-1), 34.7 (C-4), 33.1 (C-14), 31.0 (C-10, C-11), 30.5 (C-12), 30.2 (C-13), 28.3 (C-5, C-8), 27.1 (C-9), 23.9 (C-15), 22.7 (18-CH3), 14.6 (16-CH_3_); HR-ESIMS (m/z): [M + H]^+^ calcd. for C_24_H_40_NO_3_, 390.3008; found, 390.2986.

Vitroprocine E (**5**) C_22_H_37_NO_3_; white amorphous solid; [α]_D_ = –97.2 (*c* = 0.20, MeOH); UV/Vis: λ_max_ 204, 276 nm; IR (Neat): 3332 cm^−1^; ^1^H NMR (400 MHz, CD_3_OD) δ_H_ 7.01 (d, J = 8.5 Hz, 2H; H-2′ and H-6′), 6.67 (d, J = 8.5 Hz, 2H; H-3′ and H-5′), 3.96 (m, 1H; H-2), 3.49 (m, 1H; H-3), 2.92 (dd, J = 14.0, 4.0 Hz, 1H; H-1), 2.53 (dd, J = 14.0, 10.2 Hz, 1H; H-1), 1.89 (s, 3H; 16-CH_3_), 1.25~1.60 (m; aliphatic CH_2_), 0.90 (t, J = 6.8 Hz, 3H; 14-CH_3_); ^13^C NMR (100 MHz, CD_3_OD) δ_C_ 131.3 (C-2′ and C-6′), 116.2 (C-3′ and C-5′), 74.6 (C-3), 57.4 (C-2), 35.9 (C-1), 34.7 (C-4), 33.2 (C-12), 30.9 (C-6, C-7, C-8, C-9, C-10), 30.6 (C-11), 27.2 (C-5), 23.9 (C-13), 22.7 (16-CH_3_), 14.6 (14-CH_3_); HR-ESIMS (m/z): [M + H]^+^ (calcd. for C_22_H_38_NO_3_, 364.2852; found, 364.2831.

Vitroprocine F (**6**) C_22_H_35_NO_3_; white amorphous solid; [α]_D_ = –26.9 (*c = *0.550, MeOH); UV/Vis: λ_max_ 205, 274 nm; IR (Neat): 3310 cm^−1^; ^1^H NMR (400 MHz, CD_3_OD) δ_H_ 7.01 (d, J = 8.5 Hz, 2H; H-2′ and H-6′), 6.67 (d, J = 8.5 Hz, 2H; H-3′ and H-5′), 5.36 (m, 2H; H-6 and H-7), 3.96 (m, 1H; H-2), 3.49 (m, 1H; H-3), 2.91 (dd, J = 14.0, 4.0 Hz, 1H; H-1), 2.53 (dd, J = 14.0, 10.1 Hz, 1H; H-1), 2.04 (m, 4H; H-5 and H-8), 1.89 (s, 3H; 16-CH_3_), 1.25~1.60 (m; aliphatic CH_2_), 0.90 (t, J = 6.8 Hz, 3H; 14-CH_3_); ^13^C NMR (100 MHz, CD_3_OD) δ_C_ 131.3 (C-2′ and C-6′), 131.1 (C-6), 130.7 (C-7), 116.1 (C-3′ and C-5′), 74.5 (C-3), 57.4 (C-2), 35.9 (C-1), 34.4 (C-4), 33.1 (C-12), 31.0 (C-10), 30.2 (C-11), 28.4 (C-5), 28.3 (C-8), 27.4 (C-9), 23.9 (C-13), 22.7 (16-CH_3_), 14.6 (14-CH_3_); HR-ESIMS (m/z): [M + H]^+^ calcd. for C_22_H_36_NO_3_, 362.2695; found, 362.2675.

Vitroprocine G (**7**) C_26_H_43_NO_3_; white amorphous solid; UV/Vis: λ_max_ 204, 224, 278 nm; IR (Neat): 3345 cm^−1^; ^1^H NMR (400 MHz, CD_3_OD) δ_H_ 7.01 (d, J = 8.4 Hz, 2H; H-2′ and H-6′), 6.67 (d, J = 8.4 Hz, 2H; H-3′ and H-5′), 5.35 (m, 2H; H-6 and H-7), 3.96 (m, 1H; H-2), 3.50 (m, 1H; H-3), 2.91 (dd, J = 14.0, 4.0 Hz, 1H; H-1), 2.53 (dd, J = 14.0, 10.2 Hz, 1H; H-1), 2.03 (m, 4H; H-5 and H-8), 1.82 (s, 3H; 20-CH_3_), 1.25~1.60 (m, aliphatic CH_2_), 0.90 (t, J = 6.8 Hz, 3H; 18-CH_3_); ^13^C NMR (100 MHz, CD_3_OD) δ_C_ 173.2 (C-19), 156.9 (C-4′), 131.3 (C-2′ and C-6′), 131.1 (C-1′), 131.0 (C-6 and C-7), 116.1 (C-3′ and C-5′), 74.6 (C-3), 57.5 (C-2), 35.9 (C-1), 34.7 (C-4), 33.1 (C-16), 31.0 (C-10, C-11), 30.9 (C-12), 30.8 (C-13), 30.5 (C-14), 30.2 (C-15), 28.3 (C-5 and C-8), 27.2 (C-9), 23.9 (C-17), 22.7 (20-CH_3_), 14.6 (18-CH_3_); HR-ESIMS (m/z): [M + H]^+^ calcd. for C_26_H_44_NO_3_, 418.3321; found, 418.3299.

Vitroprocine H (**8**) C_20_H_33_NO; white amorphous solid; [α]_D_ = –8.0 (*c* *=* 0.30, MeOH); UV/Vis: λ_max_ 208, 258 nm; IR (Neat): 3411 cm^−1^; ^1^H NMR (500 MHz, CD_3_OD) and ^13^C NMR (125 MHz, CD_3_OD) is shown in Supplementary Table S1; HR-ESIMS (m/z): [M + H]^+^ calcd. for C_20_H_34_NO, 304.2640; found, 304.2623.

Vitroprocine I (**9**) C_22_H_37_NO_2_; white amorphous solid; [α]_D_ = –57.1 (*c* = 0.275, MeOH); UV/Vis: λ_max_ 210, 260 nm; IR (Neat): 3289 cm^−1^; ^1^H NMR (500 MHz, CD_3_OD) δ_H_ 7.20 (m, 5H; H-2′~H-6′), 4.03 (m, 1H; H-2), 3.52 (m, 1H; H-3), 3.03 (dd, J = 14.3, 4.5 Hz, 1H; H-1), 2.62 (dd, J = 14.3, 10.0 Hz, 1H; H-1), 1.90 (s, 3H; 14-CH_3_), 1.25~1.60 (m; aliphatic CH_2_), 0.90 (t, J = 7.0 Hz, 3H; 14-CH_3_); ^13^C NMR (125 MHz, CD_3_OD) δ_C_ 173.1 (C-15), 140.5 (C-1′), 130.4 (C-2′ and C-6′), 129.3 (C-3′ and C-5′), 127.3 (C-4′), 74.7 (C-3), 57.2 (C-2), 36.8 (C-1), 34.8 (C-4), 33.2 (C-12), 30.9 (C-6, C-7, C-8, C-9, C-10), 30.6 (C-11), 27.2 (C-5), 23.9 (C-13), 22.7 (16-CH_3_), 14.6 (14-CH_3_); HR-ESIMS (m/z): [M + H]^+^ calcd. for C_22_H_38_NO_2_, 348.2903; found, 348.2882.

Vitroprocine J (**10**) C_22_H_35_NO_2_; white amorphous solid; [α]_D_ = –169.3 (*c* = 0.150, MeOH); UV/Vis: λ_max_ 205, 259 nm; IR (Neat): 3292 cm^−1^; ^1^H NMR (400 MHz, CD_3_OD) δ_H_ 7.20 (m, 5H; H-2′~H-6′), 4.02 (m, 1H; H-2), 3.53 (m, 1H; H-3), 3.02 (dd, J = 14.0, 3.9 Hz, 1H; H-1), 2.62 (dd, J = 14.0, 10.4 Hz, 1H; H-1), 2.07 (m, 4H; H-5 and H-8), 1.90 (s, 3H; 16-CH_3_), 1.25~1.60 (m; aliphatic CH_2_), 0.90 (t, J = 6.9 Hz, 3H; 14-CH_3_); ^13^C NMR (100 MHz, CD_3_OD) δ_C_ 172.7 (C-15), 140.4 (C-1′), 131.3 (C-6), 130.7 (C-7), 130.4 (C-2′ and C-6′), 129.3 (C-3′ and C-5′), 127.3 (C-4′), 74.6 (C-3), 57.2 (C-2), 36.7 (C-1), 34.3 (C-4), 33.2 (C-12), 31.0 (C-10), 30.2 (C-11), 28.4 (C-5), 28.3 (C-8), 27.4 (C-9), 23.9 (C-13), 22.6 (16-CH_3_), 14.6 (14-CH_3_); HR-ESIMS (m/z): [M + H]^+^ (calcd. for C_22_H_36_NO_2_, 346.2746; found, 346.2726.

## Additional Information

**How to cite this article**: Liaw, C.-C. *et al.* Vitroprocines, new antibiotics against *Acinetobacter baumannii*, discovered from marine *Vibrio* sp. QWI-06 using mass-spectrometry-based metabolomics approach. *Sci. Rep.* **5**, 12856; doi: 10.1038/srep12856 (2015).

## Supplementary Material

Supplementary Information
