# Supplementary material for: Vitroprocines, new antibiotics against Acinetobacter baumannii, discovered from marine Vibrio sp. QWI-06 using mass-spectrometry-based metabolomics approach
Source: Sci Rep. 2015 Aug 4;5:12856. doi: 10.1038/srep12856 (PMC4523872; doi:10.1038/srep12856)
Supplement: Supplementary Information [file srep12856-s1.pdf]

Supplementary Information

Vitroprocines, new antibiotics against *Acinetobacter baumannii*, discovered from marine *Vibrio* sp. QWI-06 using mass-spectrometry-based metabolomics approach

Chih-Chuang Liaw[1,2,3]*, Pei-Chin Chen[1], Chao-Jen Shih[4], Sung-Pin Tseng[5], Ying-Mi Lai[4], Chi-Hsin Hsu[1,2], Pieter C. Dorrestein[6], and Yu-Liang Yang[2,3,4]*

[1]Department of Marine Biotechnology and Resources, National Sun Yat-sen University, Kaohsiung 80424, Taiwan

[2]Doctoral Degree Program in Marine Biotechnology, National Sun Yat-sen University, Kaohsiung 80424, Taiwan

[3]Graduate Institute of Natural Products, Kaohsiung Medical University, Kaohsiung 807, Taiwan

[4]Agricultural Biotechnology Research Center, Academia Sinica, Taipei 11529, Taiwan

[5]Department of Medical Laboratory Sciences and Biotechnology, Kaohsiung Medical University, Kaohsiung 807, Taiwan

[6]Skaggs School of Pharmacy and Pharmaceutical Sciences, University of California, San Diego, La Jolla, CA 92093-0636, United States

*Corresponding. ccliaw@mail.nsysu.edu.tw, ylyang@gate.sinica.edu.tw

Contents of Supplementary Information

	Contents	Pages
**Experimental Section**		**5**
**Table S1**	^{1}H (500 MHz) and ^{13}C NMR (125 MHz) Data of **1-3** and **8** in [D_4]MeOH	**6**
**Figure S1**.	Anti-microbial assay of *Vibrio* sp. QWI-06 crude extract (1, 100 μg) and compound **1** (2-7, 40 μg)	**7**
**Figure S2**.	Phylogenetic analysis of *Vibrio* sp. QWI-06	**7**
**Figure S3**	Intact-cell MALDI-MS analysis the colonies of *Vibrio* sp. QWI-06 (red) and *V. proteolyticus* ATCC 15338 (blue)	**8**
**Figure S4**	Full molecular networking of *Vibrio* sp. QWI-06	**8**
**Figure S5**	Mass *vs*. error distribution of vitroprocines nodes selected from molecular networking	**9**
**Table S2**	Summary of vitroprocines (I): subgroups A-D and F	**10**
**Table S3**	Summary of vitroprocines (II): subgroups E and G	**12**
**Figure S6**	HR-ESIMS/MS spectra of subgroup A	**13**
**Figure S7**	HR-ESIMS/MS spectra of subgroup B	**14**
**Figure S8**	HR-ESIMS/MS spectra of subgroup C	**15**
**Figure S9**	HR-ESIMS/MS spectra of subgroup D	**16**
**Figure S10**	HR-ESIMS/MS spectra of subgroup E	**17**
**Figure S11**	HR-ESIMS/MS spectra of subgroup F	**18**
**Figure S12**	HR-ESIMS/MS spectra of subgroup G	**19**
**Figure S13**	UV spectrum of **1**	**20**
**Figure S14**	FTIR spectrum of **1**	**20**
**Figure S15**	^{1}H-NMR spectrum of **1**	**21**
**Figure S16**	^{13}C NMR spectrum of **1**	**21**
**Figure S17**	HSQC spectrum of **1**	**22**
**Figure S18**	HMBC spectrum of **1**	**22**
**Figure S19**	^{1}H-^{1}H COSY spectrum of **1**	**23**
**Figure S20**	^{1}H NMR Spectra of **1** and its ***R*** and ***S*** Mosher derivatives in $CDCl_3$	**23**
**Figure S21**	EIMS spectrum and fragmentation of the dimethyl disulfide derivative of **1**	**24**
**Figure S22**	UV spectrum of **2**	**25**
**Figure S23**	FTIR spectrum of **2**	**25**
**Figure S24**	^{1}H NMR spectrum of **2**	**26**
**Figure S25**	^{13}C NMR spectrum of **2**	**26**
**Figure S26**	HSQC spectrum of **2**	**27**
**Figure S27**	HMBC spectrum of **2**	**27**
**Figure S28**	^{1}H-^{1}H COSY spectrum of **2**	**28**

**Figure S29**	EIMS spectrum and fragmentation of the dimethyl disulfide derivative of **2**	**28**
**Figure S30**	UV spectrum of **3**	**29**
**Figure S31**	FTIR spectrum of **3**	**29**
**Figure S32**	^{1}H NMR spectrum of **3**	**30**
**Figure S33**	^{13}C NMR spectrum of **3**	**30**
**Figure S34**	HSQC spectrum of **3**	**31**
**Figure S35**	HMBC spectrum of **3**	**31**
**Figure S36**	^{1}H-^{1}H COSY spectrum of **3**	**32**
**Figure S37**	UV spectrum of **4**	**33**
**Figure S38**	FTIR spectrum of **4**	**33**
**Figure S39**	^{1}H NMR spectrum of **4**	**34**
**Figure S40**	^{13}C NMR spectrum of **4**	**34**
**Figure S41**	HSQC spectrum of **4**	**35**
**Figure S42**	HMBC spectrum of **4**	**35**
**Figure S43**	^{1}H-^{1}H COSY spectrum of **4**	**36**
**Figure S44**	EIMS spectrum and fragmentation of the dimethyl disulfide derivative of **4**	**36**
**Figure S45**	UV spectrum of **5**	**37**
**Figure S46**	FTIR spectrum of **5**	**37**
**Figure S47**	^{1}H NMR spectrum of **5**	**38**
**Figure S48**	^{13}C NMR spectrum of **5**	**38**
**Figure S49**	UV spectrum of **6**	**39**
**Figure S50**	FTIR spectrum of **6**	**39**
**Figure S51**	^{1}H NMR spectrum of **6**	**40**
**Figure S52**	^{13}C NMR spectrum of **6**	**40**
**Figure S53**	EIMS spectrum and fragmentation of the dimethyl disulfide derivative of **6**	**41**
**Figure S54**	UV spectrum of **7**	**42**
**Figure S55**	FTIR spectrum of **7**	**42**
**Figure S56**	^{1}H NMR spectrum of **7**	**43**
**Figure S57**	^{13}C NMR spectrum of **7**	**43**
**Figure S58**	EIMS spectrum and fragmentation of the dimethyl disulfide derivative of **7**	**44**
**Figure S59**	UV spectrum of **8**	**45**
**Figure S60**	FTIR spectrum of **8**	**45**
**Figure S61**	^{1}H NMR spectrum of **8**	**46**
**Figure S62**	^{13}C NMR spectrum of **8**	**46**
**Figure S63**	HSQC spectrum of **8**	**47**
**Figure S64**	HMBC spectrum of **8**	**47**
**Figure S65**	^{1}H-^{1}H COSY spectrum of **8**	**48**

**Figure S66**	EIMS spectrum and fragmentation of the dimethyl disulfide derivative of **8**	**48**
**Figure S67**	UV spectrum of **9**	**49**
**Figure S68**	FTIR spectrum of **9**	**49**
**Figure S69**	^{1}H NMR spectrum of **9**	**50**
**Figure S70**	^{13}C NMR spectrum of **9**	**50**
**Figure S71**	UV spectrum of **10**	**51**
**Figure S72**	FTIR spectrum of **10**	**51**
**Figure S73**	^{1}H NMR spectrum of **10**	**52**
**Figure S74**	^{13}C NMR spectrum of **10**	**52**
**Figure S75**	EIMS spectrum and fragmentation of the dimethyl disulfide derivative of **10**	**53**
**Figure S76**	Quantitation results of **1**, **3**, and **8** from *Vibrio* sp. QWI-06 under different cultural condition	**54**
**Figure S77**	HR-ESIMS/MS spectrum and fragmentation of **1** from *Vibrio* sp. QWI-06 feed with $^{13}C_9$-tyrosine	**55**
**Figure S78**	^{13}C NMR spectra of **1** and **1a**	**56**

Experimental Section

Instrumentation. Optical rotations were measured on a JASCO P1020 digital polarimeter at 25 °C. UV spectra were recorded on a JASCO V-650 spectrophotometer. IR spectra were obtained on a JASCO FT/IR-4100 infrared spectrophotometer. NMR spectra were recorded with Bruker 500 AVII and Varian Unity 400 FT NMR spectrometers. HPLC-ESIMS data were processed using an AmaZon SL ion trap mass spectrometer (Bruker Daltonics, Bremen, Germany) and UPLC-HR-ESIMS and MS/MS data were acquired on a Thermo Orbitrap Elite system and a Thermo TSQ Quantum Access MAX Triple Quadrupole system. IMS data were acquired in a Bruker autoflex™ speed MALDI-TOF/TOF system. Extracts were chromatographed on Sephadex LH-20 (Amersham Biosciences) and purified using a semi-preparative RP HPLC column (Supelco Discovery C18, 250 x 10 mm, 5 μm). HPLC was performed on a Hitachi L-2130 apparatus equipped with a Hitachi L-2420 UV-VIS detector.

Minimum inhibitory concentration (MIC) assay. The MICs of compounds against *A. baumannii* ATCC 19606 were determined using the broth microdilution method as described previously.[1] Briefly, *A. baumannii* was inoculated in BHI agar at 37 °C overnight then diluted with CAMHB broth to adjust the turbidity approximately to the standard McFarland 0.5 (2×10^8 CFU/mL).[2] Then the bacterial suspension was further diluted by 200 times with CAMHB broth to achieve an initial loading of 1×10^6 CFU/mL. Compounds resolved in DMSO were series diluted in H_2O at various concentrations. The bacterial suspension (100 μL) was mixed with an equal volume of sample solution at various concentrations in each well of a 96-well plate, and incubated for 20~24 h at 37 °C. The MIC was defined as the lowest concentration, which no visible turbidity was detected with unaided eyes and a microplate reader (Bio-Rad) in OD600. Broth with bacteria only was used as a negative control, ceftazidime and ticarcillin were used as positive control (MICs of both drugs are 8 μg/mL), and all the data were measured in triplicate.

Biosynthetic studies. 1-^{13}C-sodium acetate and $^{13}C_9$-tyrosine were used (99% of ^{13}C, Cambridge Isotope Laboratories, Isotec, and Aldrich) for the isotope labeling experiments. *Vibrio* sp. QWI-06 was cultivated in Difco marine agar. For each experiment, 0.1 mM of 1-^{13}C-sodium acetate and $^{13}C_9$-tyrosine were respectively added in the sterile agar medium for 50 Petri dish plates (90 × 15 mm). Following the inoculum, all the cultures were grown for three days at 30 °C. The isolation of labeled vitroprocine A (**1**) was carried out according to the aforementioned procedure and the compound was subjected to NMR and LC-HR-ESIMS/MS analysis (Supporting information Figures S77 and S78). The yields of 1-^{13}C-sodium acetate labeled vitroprocine A (**1a**) were approximately 0.2 mg/plate.

References

[1] *Performance standards for antimicrobial susceptibility testing: twenty-third informational supplement, Vol. 33*, Clinical and Laboratory Standards Institute, Wayne, PA, **2013**.

[2] a) I. Wiegand, K. Hilpert, R. E. Hancock, *Nat. Protoc.* **2008**, *3*, 163-175; b) Y. Huang, N. Wiradharma, K. Xu, Z. Ji, S. Bi, L. Li, Y. Y. Yang, W. Fan, *Biomaterials* **2012**, *33*, 8841-8847.

Table S1. ^{1}H (500 MHz) and ^{13}C NMR (125 MHz) Data of **1-3** and **8** in [D$_4$]MeOH.

	**1**		**2**		**3**		**8**	
	δ_H (*J* in Hz)	δ_C	δ_H[a] (*J* in Hz)	δ_C[b]	δ_H (*J* in Hz)	δ_C	δ_H (*J* in Hz)	δ_C
**1**	2.92, dd (14.4, 5.5)	33.8 CH_2	2.92, dd (14.5, 5.6)	33.9 CH_2	2.92, dd (14.5, 5.6)	33.8 CH_2	3.01, dd (14.3, 5.3)	35.7 CH_2
	2.72, dd (14.4, 9.0)		2.72, dd (14.5, 9.2)		2.73, dd (14.5, 9.0)		2.76, dd (14.3, 9.1)	
**2**	3.38, ddd (9.0, 5.5, 3.2)	59.2 CH	3.38, ddd (9.2, 5.6, 3.1)	59.2 CH	3.39, ddd (9.0, 5.6, 3.2)	59.2 CH	3.37, m	58.9 CH
**3**	3.74, dt (9.0, 3.2)	71.5 CH	3.73, dt (9.2, 3.1)	71.5 CH	3.74, m	71.4 CH	3.70, m	72.3 CH
**4**	1.25~1.60, m	33.1 CH_2	2.04, m	28.3 CH_2	1.25~1.40, m	30.8 CH_2	1.25~1.60, m	33.1 CH_2
**5**	2.07, m	28.4 CH_2	5.38, m	131.2 CH	1.25~1.60, m	27.2 CH_2	1.25~1.40, m	31.1 CH_2
**6**	5.40, m	131.6 CH	5.34, m	130.7 CH	1.25~1.40, m	30.9 CH_2	1.25~1.40, m	30.2 CH_2
**7**	5.36, m	130.4 CH	2.04, m	28.3 CH_2	1.25~1.40, m	30.9 CH_2	1.25~1.60, m	27.5 CH_2
**8**	2.07, m	28.1 CH_2	1.25~1.60, m	27.1 CH_2	1.25~1.40, m	30.8 CH_2	2.05, m	28.4 CH_2
**9**	1.25~1.60, m	27.4 CH_2	1.25~1.40, m	31.0 CH_2	1.25~1.40, m	30.8 CH_2	5.40, m	131.6 CH
**10**	1.25~1.40, m	31.0 CH_2	1.25~1.40, m	30.9 CH_2	1.25~1.40, m	30.7 CH_2	5.36, m	130.4 CH
**11**	1.25~1.40, m	30.2 CH_2	1.25~1.40, m	30.3 CH_2	1.25~1.40, m	30.6 CH_2	2.05, m	28.2 CH_2
**12**	1.25~1.60, m	33.0 CH_2	1.25~1.40, m	30.2 CH_2	1.25~1.60, m	33.2 CH_2	1.25~1.60, m	33.1 CH_2
**13**	1.25~1.40, m	23.9 CH_2	1.25~1.60, m	33.3 CH_2	1.25~1.40, m	23.9 CH_2	1.25~1.40, m	23.9 CH_2
**14**	0.90, t (7.0)	14.6 CH_3	1.25~1.60, m	33.1 CH_2	0.90, t (6.9)	14.6 CH_3	0.90, t (6.5)	14.6 CH_3
**15**			1.25~1.40, m	23.9 CH_2				
**16**			0.90, t (6.8)	14.6 CH_3				
**Ar**								
**1’**		128.1 qC		128.1 qC		128.2 qC		138.5 qC
**2’**	7.10, d (8.5)	131.4 CH	7.10, d (8.5)	131.4 CH	7.10, d (8.5)	131.5 CH	7.28, d (6.8)	130.4 CH
**3’**	6.78, d (8.5)	117.0 CH	6.79, d (8.5)	117.0 CH	6.78, d (8.5)	117.3 CH	7.35, t (7.5)	130.2 CH
**4’**		158.0 qC		158.2 qC		158.1 qC	7.28, m	128.3 CH
**5’**	6.78, d (8.5)	117.0 CH	6.79, d (8.5)	117.0 CH	6.78, d (8.5)	117.3 CH	7.35, t (7.5)	130.2 CH
**6’**	7.10, d (8.5)	131.4 CH	7.10, d (8.5)	131.4 CH	7.10, d (8.5)	131.5 CH	7.28, d (6.8)	130.4 CH

[a] Data were recorded at 400 MHz and [b] 100 MHz.

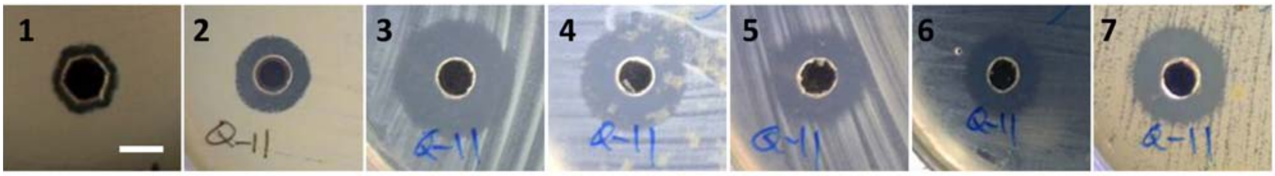

Figure S1. Anti-microbial assay of *Vibrio* sp. QWI-06 crude extract (1, 100 μg) and compound **1** (2-7, 40 μg). 1 and 2: *Acinetobacter baumanni*; 3: *Klebsiella pneumonia*; 4: *Escherichia coli*; 5: *Staphylococcus aureus*; 6: *Bacillus cereus*; 7: *Candida albicans*. Scale bar: 0.5 cm.

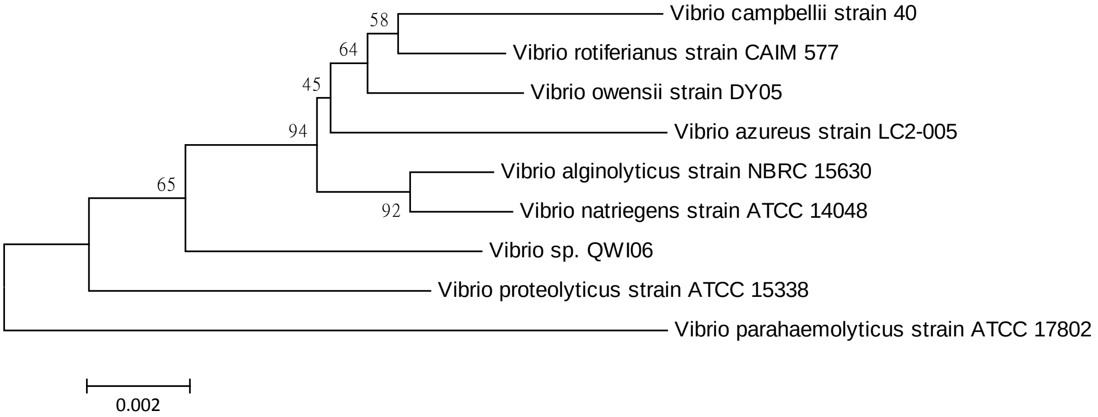

Figure S2. Phylogenetic analysis of *Vibrio* sp. QWI-06. The evolutionary history was inferred using the Neighbor-Joining method. The optimal tree with the sum of branch length = 0.05496040 is shown. The percentage of replicate trees in which the associated taxa clustered together in the bootstrap test (1000 replicates) are shown next to the branches. The tree is drawn to scale, with branch lengths in the same units as those of the evolutionary distances used to infer the phylogenetic tree. The evolutionary distances were computed using the Maximum Composite Likelihood method and are in the units of the number of base substitutions per site. The analysis involved 9 nucleotide sequences. All positions containing gaps and missing data were eliminated. There were a total of 1395 positions in the final dataset. Evolutionary analyses were conducted in MEGA5. The accession numbers of *Vibrio* type strains used for analysis: *V. alginolyticus* NBRC 15630 (NR_122060), *V. parahaemolyticus* ATCC 17802 (NR_118928), *V. campbellii* strain 40 (NR_029222), *V. azureus* LC2-005 (NR_117997), *V. natriegens* ATCC 14048 (NR_117890), *V. rotiferianus* CAIM 577 (NR_042081), *V. proteolyticus* ATCC 15338 (NR_118927), *V. owensii* DY05 (NR_117424).

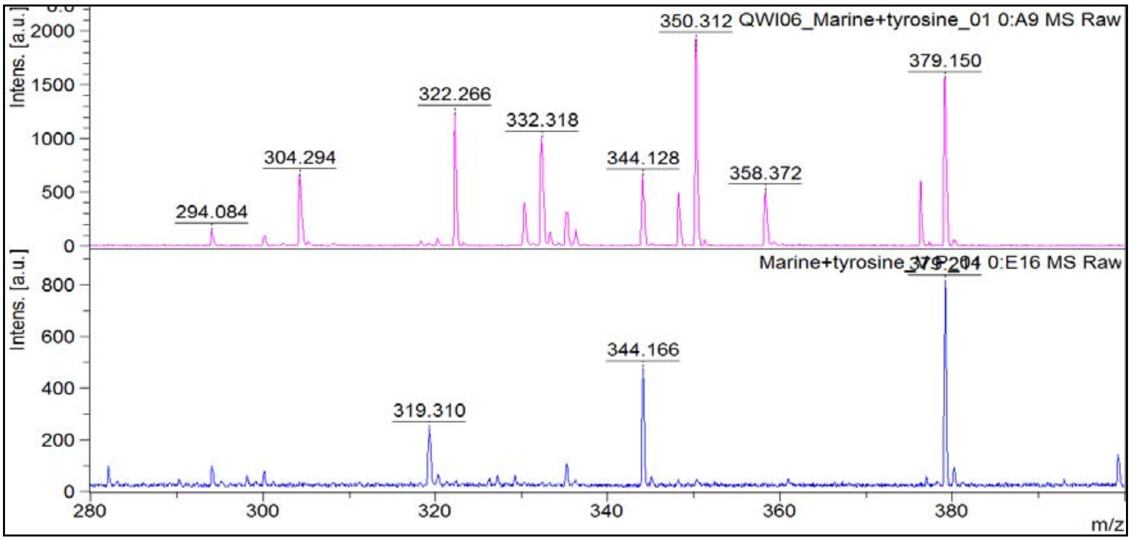

Figure S3. Intact-cell MALDI-MS analysis the colonies of *Vibrio* sp. QWI-06 (red) and *V. proteolyticus* ATCC 15338 (blue).

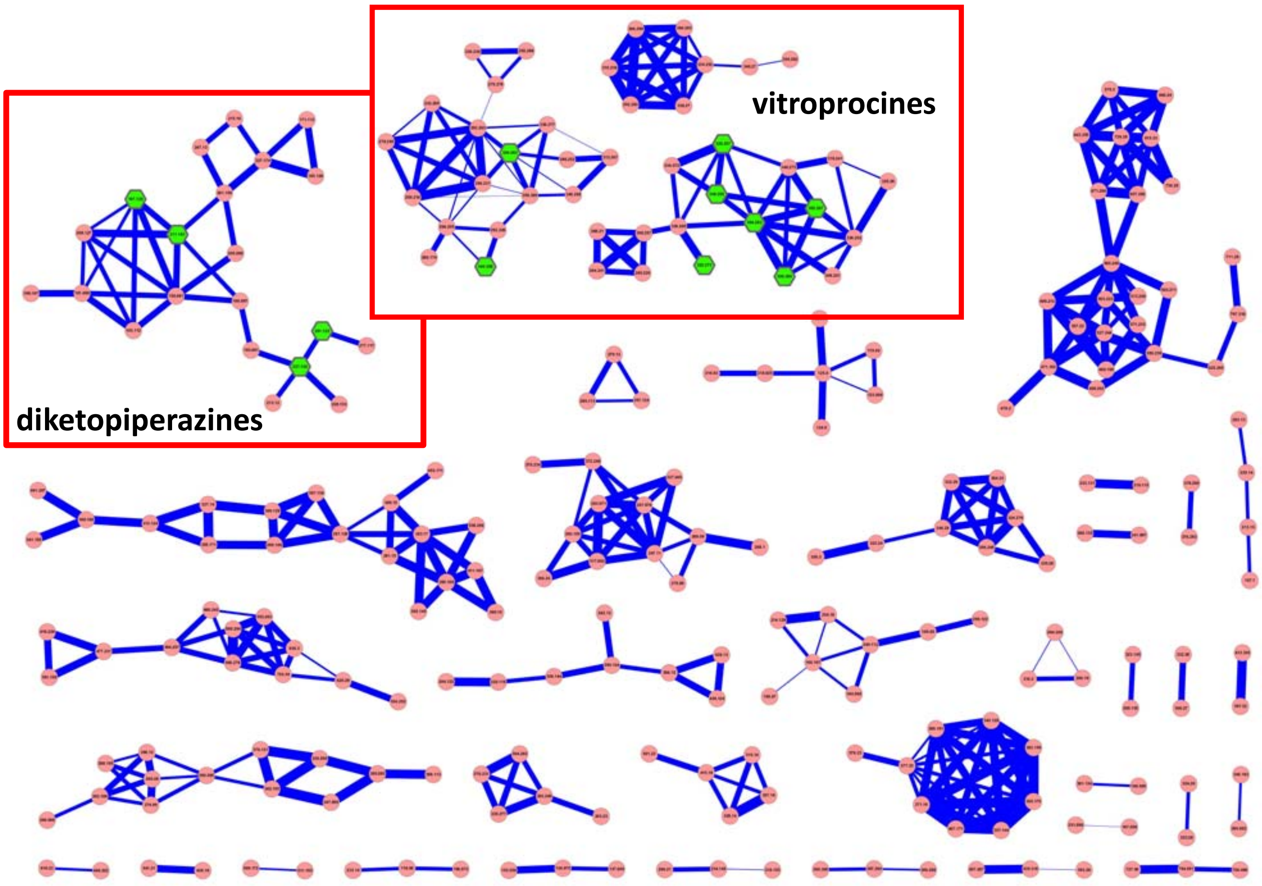

Figure S4. Full molecular networking of *Vibrio* sp. QWI-06.

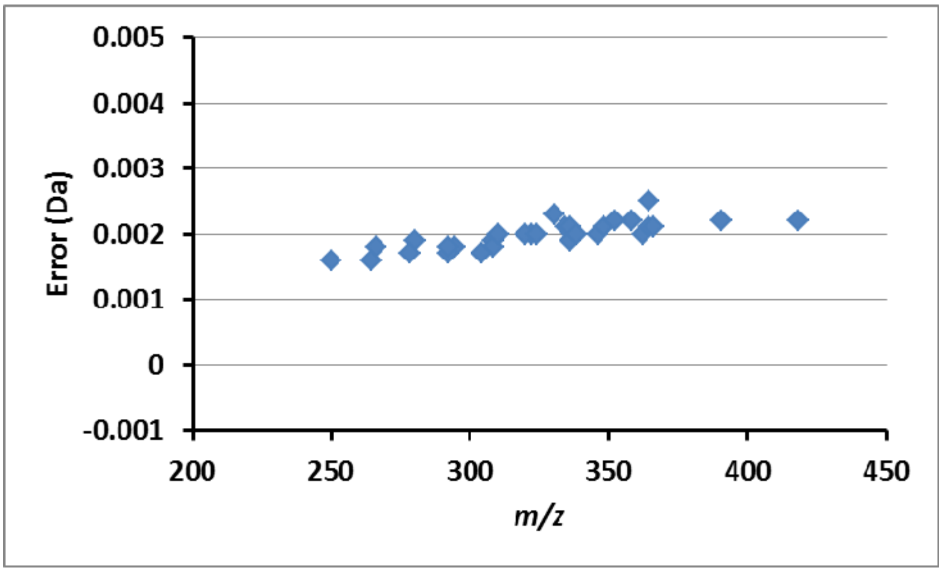

Figure S5. Mass *vs*. error distribution of vitroprocines nodes selected from molecular networking

Table S2. Summary of vitroprocines (I): subgroups A-D and F

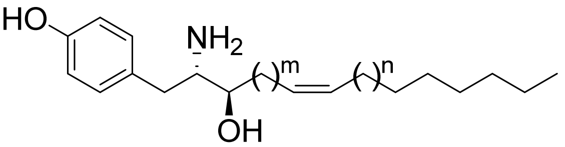

1: m = 2, n = 1
2: m = 1, n = 4

	[M+H]+	Found	Calcd.	Error (Da)	Isolated	Networking	Double bond
A1	$C_{20}H_{34}NO_2$	320.2570	320.2590	0.0020	**1**	V	$\Delta_{6,7}$
A2	$C_{21}H_{36}NO_2$	334.2725	334.2746	0.0021	-	V	DNC*
A3	$C_{22}H_{38}NO_2$	348.2882	348.2903	0.0021	**2**	V	$\Delta_{5,6}$

*Does not confirm (DNC)

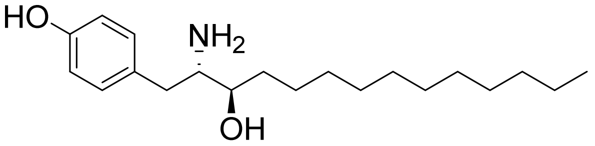

3

	[M+H]+	Found	Calcd.	Error (Da)	Isolated	Networking
B1	$C_{16}H_{28}NO_2$	266.2102	266.2120	0.0018	-	V
B2	$C_{17}H_{30}NO_2$	280.2258	280.2277	0.0019	-	V
B3	$C_{18}H_{32}NO_2$	294.2415	294.2433	0.0018	-	V
B4	$C_{19}H_{34}NO_2$	308.2571	308.2590	0.0019	-	V
B5	$C_{20}H_{36}NO_2$	322.2726	322.2746	0.0020	**3**	V
B6	$C_{21}H_{38}NO_2$	336.2884	336.2903	0.0019	-	V

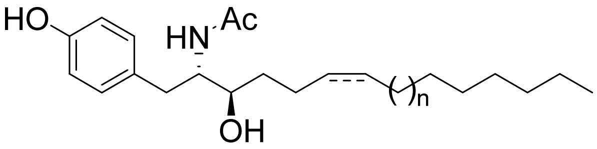

4: n = 3
5: n = 1, saturated
6: n = 1
7: n = 5

	[M+H]+	Found	Calcd.	Error (Da)	Isolated	Networking	Double bond
C1	$C_{18}H_{30}NO_3$	308.2208	308.2226	0.0018	-	V	-
C2	$C_{20}H_{34}NO_3$	336.2518	336.2539	0.0021	-	V	-
C3	$C_{22}H_{36}NO_3$	362.2675	362.2695	0.0020	**6**	V	$\Delta_{6,7}$
C4	$C_{22}H_{38}NO_3$	364.2831	364.2852	0.0021	**5**	V	-
C5	$C_{24}H_{40}NO_3$	390.2986	390.3008	0.0022	**4**	V	$\Delta_{6,7}$
C6	$C_{26}H_{44}NO_3$	418.3299	418.3321	0.0022	**7**	-*	$\Delta_{6,7}$

*C6 was detected in LC-MS but the intensity was too low to be selected for data-dependent LC-MS/MS analysis

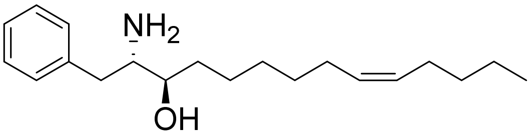

8

	$[M+H]^+$	Found	Calcd.	Error (Da)	Isolated	Networking	Double bond
D1	$C_{20}H_{34}NO$	304.2623	304.2640	0.0017	**8**	V	$\Delta_{9,10}$
D2	$C_{22}H_{36}NO$	330.2774	330.2797	0.0023	-	V	DNC*
D3	$C_{24}H_{40}NO$	358.3088	358.3110	0.0022	-	V	DNC*

*Two double bonds are on the aliphatic chain but the positions are unidentified.

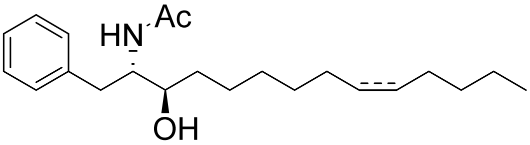

9: saturated
10

	$[M+H]^+$	Found	Calcd.	Error (Da)	Isolated	Networking	Double bond
F1	$C_{18}H_{30}NO_2$	292.2259	292.2277	0.0018	-	V	-
F2	$C_{22}H_{36}NO_2$	346.2726	346.2746	0.0020	**10**	-*	$\Delta_{9,10}$
F3	$C_{22}H_{38}NO_2$	348.2882	348.2903	0.0021	**9**	V	-

*F2 was detected in LC-MS but the intensity was too low to be selected for data-dependent LC-MS/MS analysis

Table S3. Summary of vitroprocines (II): subgroups E and G. The structures of E1 and G1 were proposed based on the HR-ESIMS/MS fragmentations. The stereochemistry of C-2 in subgroup E and C-2, C-3 in subgroup G were proposed based on the same biosynthetic origin of vitroprocines A-J (**1-10**).

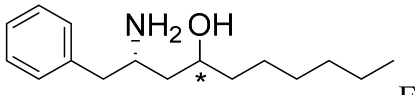

E1

	$[M+H]^+$	Found	Calcd.	Error (Da)
E1	$C_{16}H_{28}NO$	250.2155	250.2171	0.0016
E2	$C_{17}H_{30}NO$	264.2311	264.2327	0.0016
E3	$C_{18}H_{32}NO$	278.2467	278.2484	0.0017
E4	$C_{19}H_{34}NO$	292.2623	292.2640	0.0017

*The stereochemistry is unidentified.

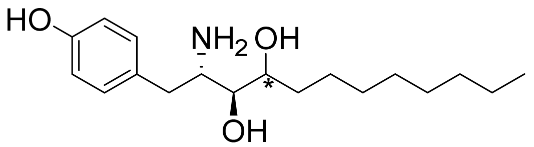

G1

	$[M+H]^+$	Found	Calcd.	Error (Da)	Double bond
G1	$C_{18}H_{32}NO_3$	310.2362	310.2382	0.0020	-
G2	$C_{19}H_{34}NO_3$	324.2519	324.2539	0.0020	-
G3	$C_{20}H_{36}NO_3$	338.2675	338.2695	0.0020	-
G4	$C_{21}H_{38}NO_3$	352.2830	352.2852	0.0022	-
G5	$C_{22}H_{38}NO_3$	364.2827	364.2852	0.0025	DNC
G6	$C_{22}H_{40}NO_3$	366.2987	366.3008	0.0021	-

*The stereochemistry is unidentified.

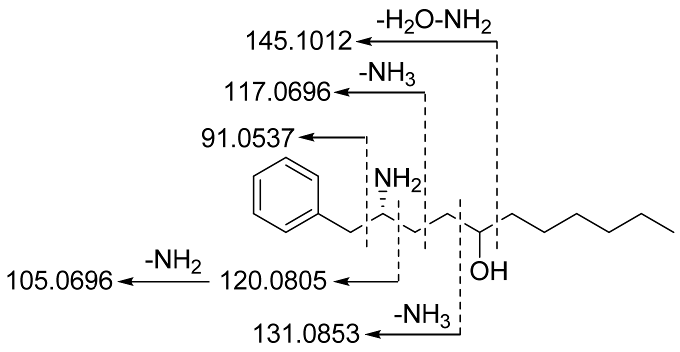

E1

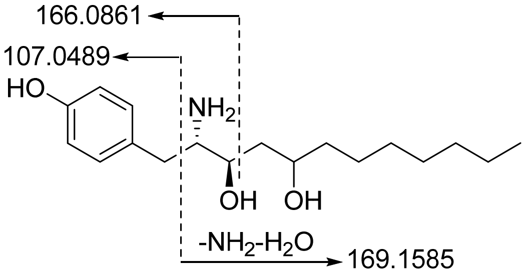

G1

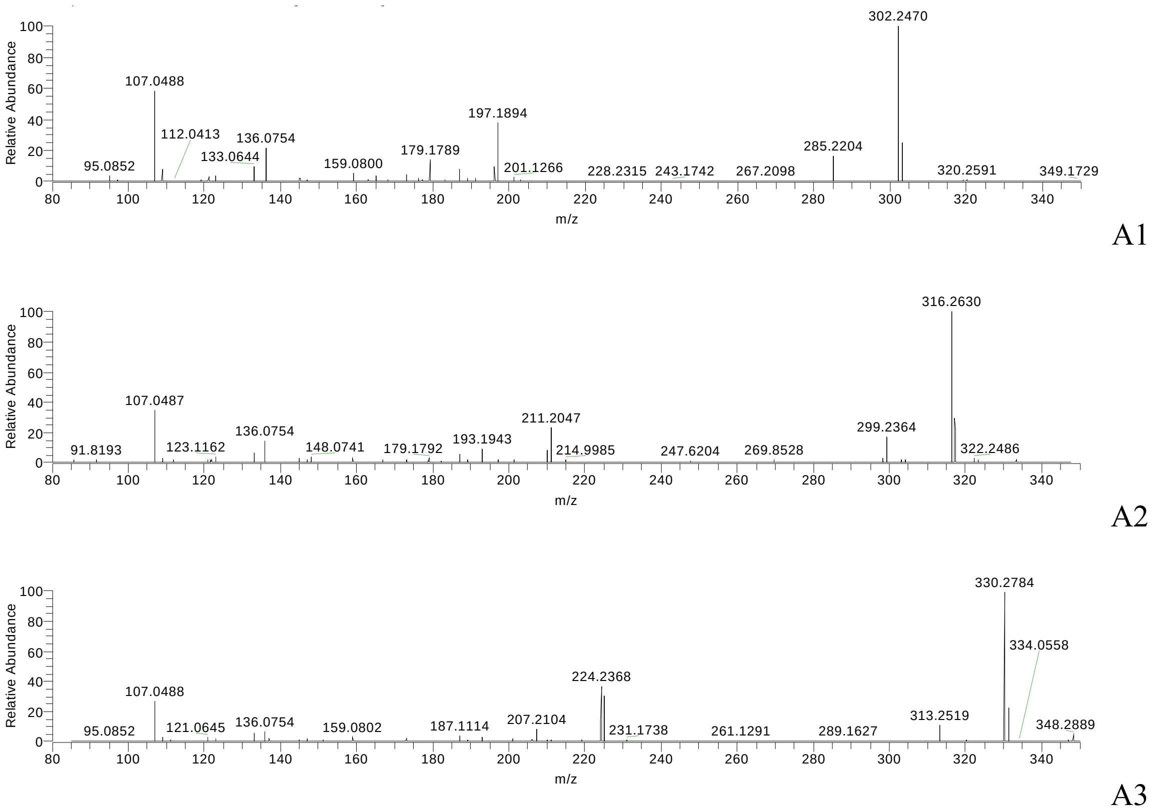

Figure S6. HR-ESIMS/MS spectra of subgroup A

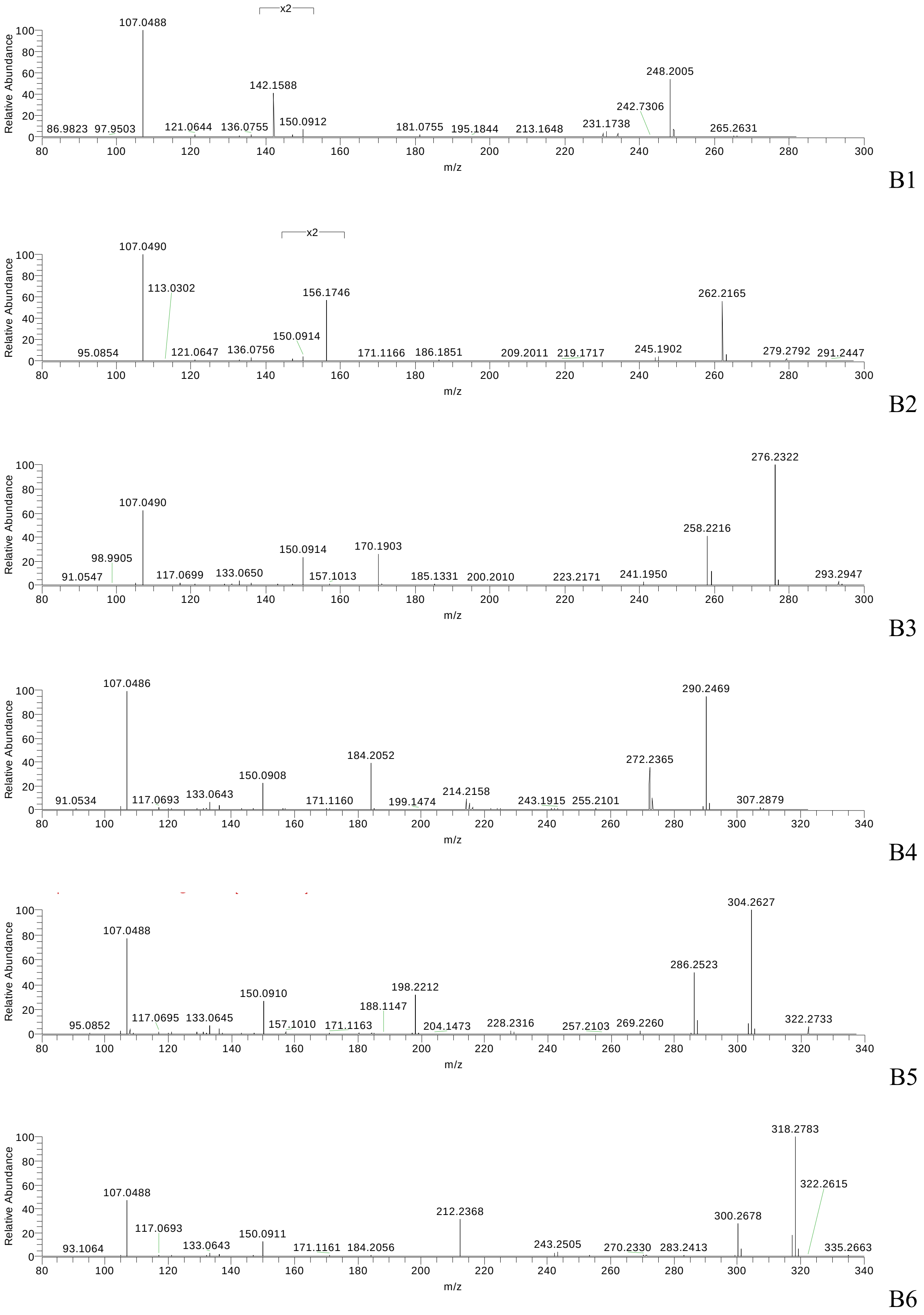

Figure S7. HR-ESIMS/MS spectra of subgroup B

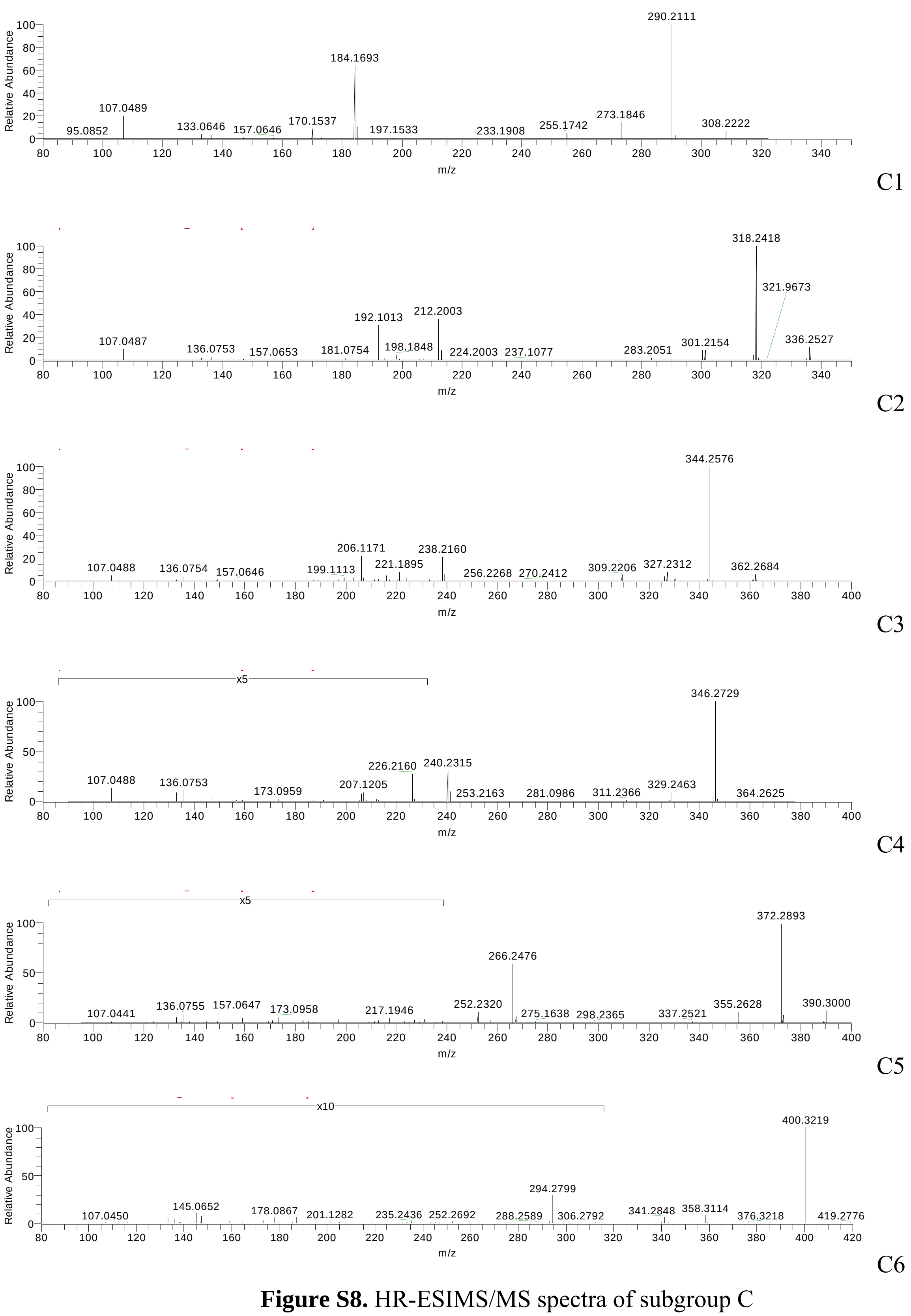

Figure S8. HR-ESIMS/MS spectra of subgroup C

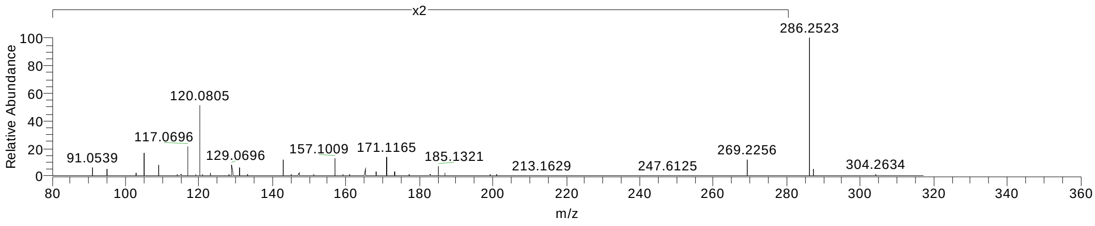

D1

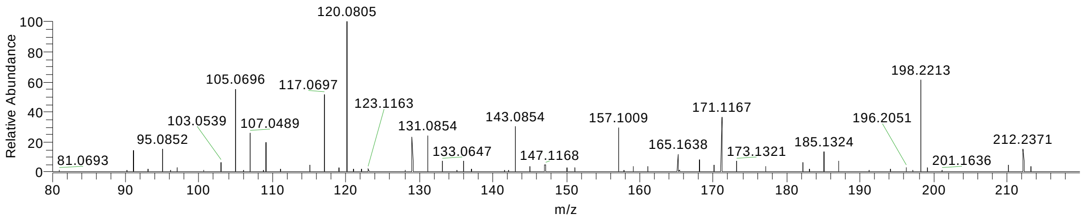

D1*

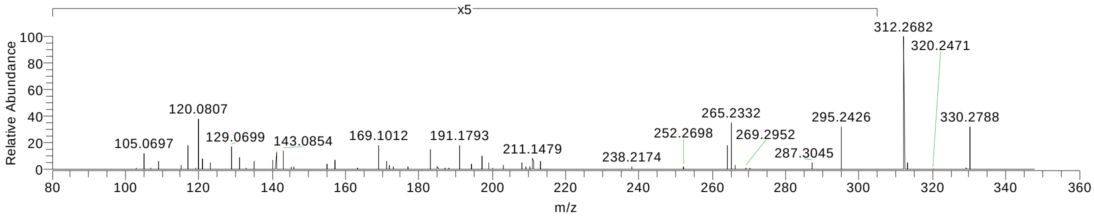

D2

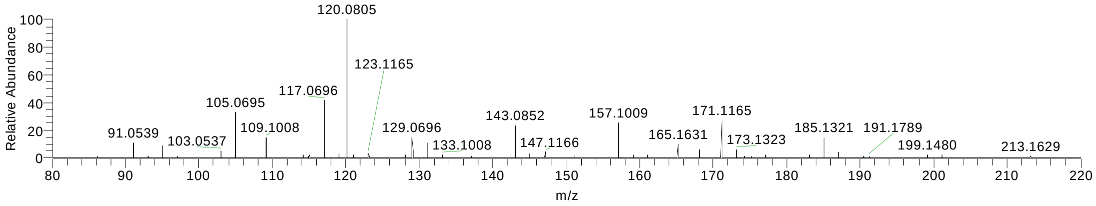

D2*

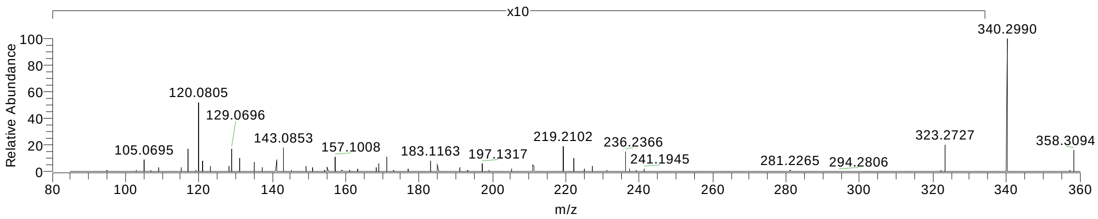

D3

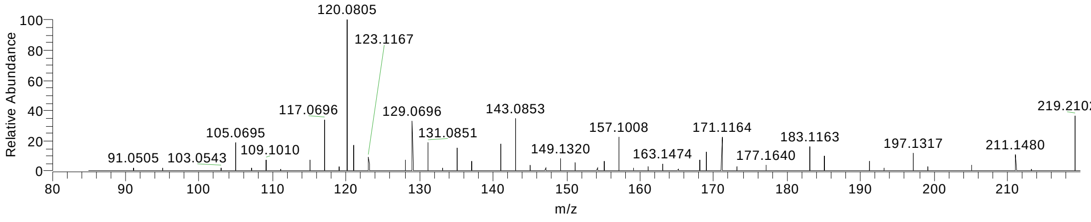

D3*

Figure S9. HR-ESIMS/MS spectra of subgroup D (*expanded spectra)

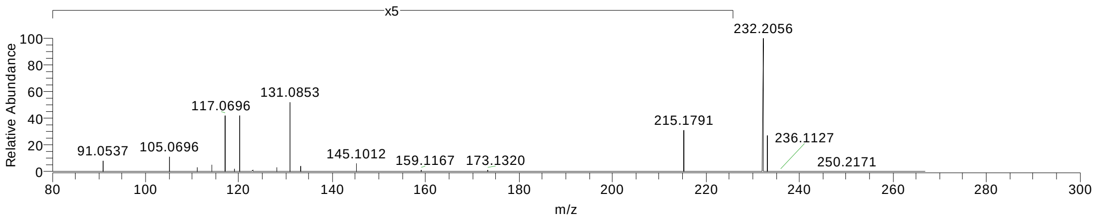

E1

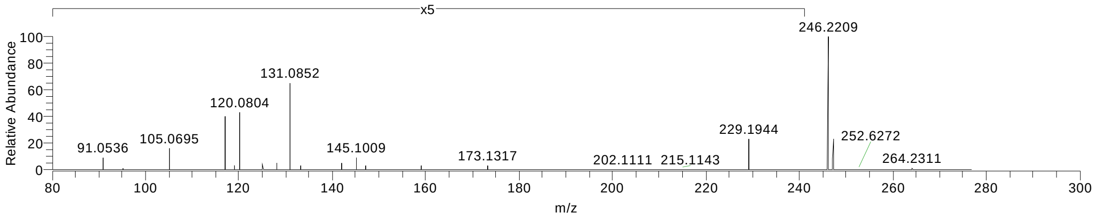

E2

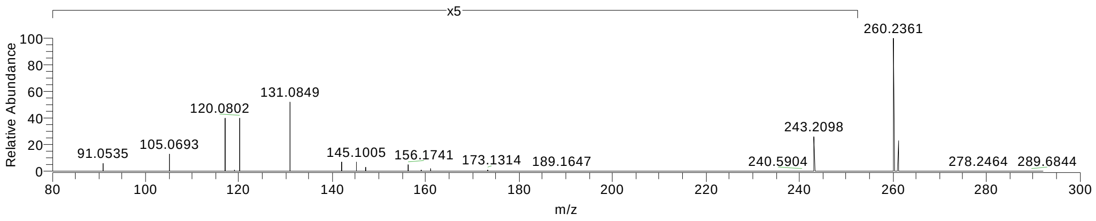

E3

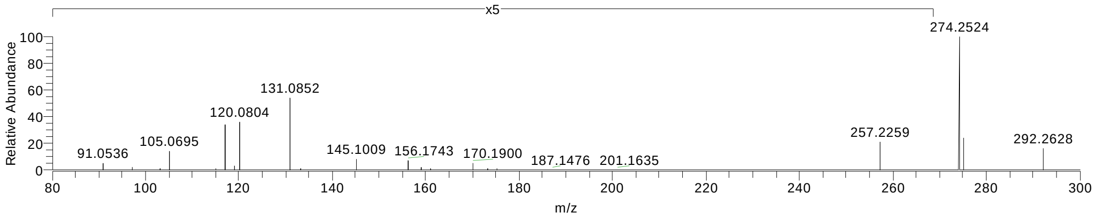

E4

Figure S10. HR-ESIMS/MS spectra of subgroup E

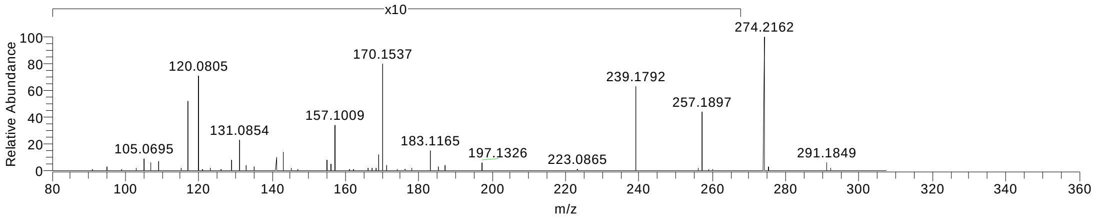

F1

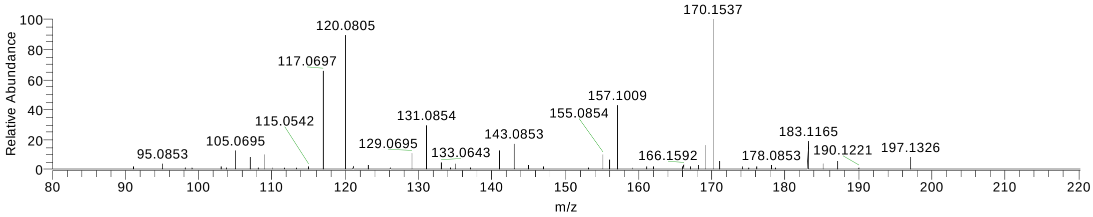

F1*

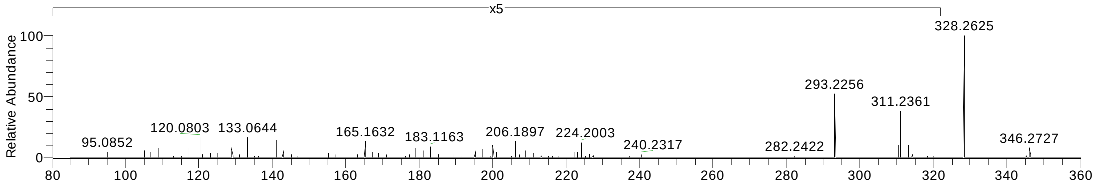

F2

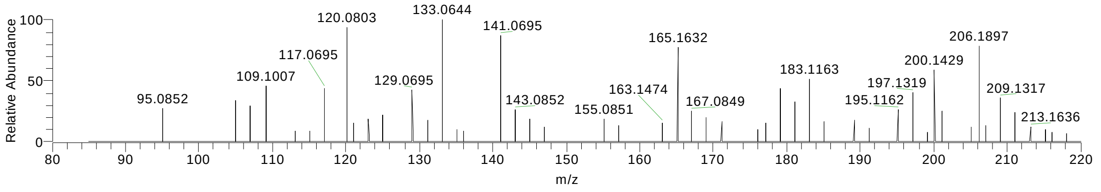

F2*

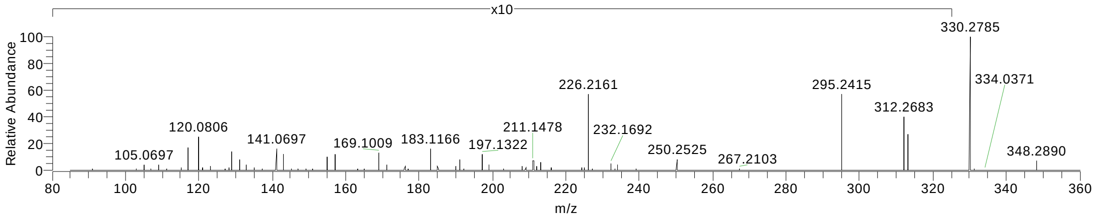

F3

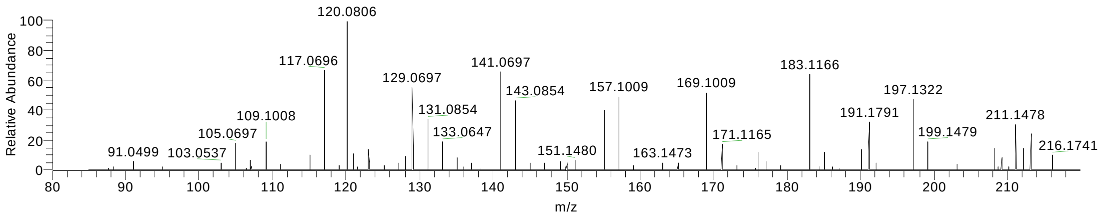

F3*

Figure S11. HR-ESIMS/MS spectra of subgroup F (*expanded spectra)

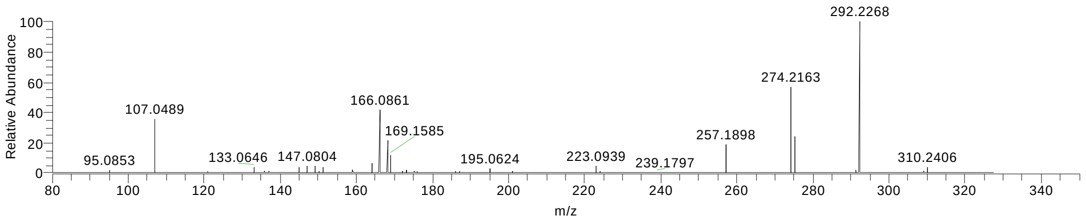

G1

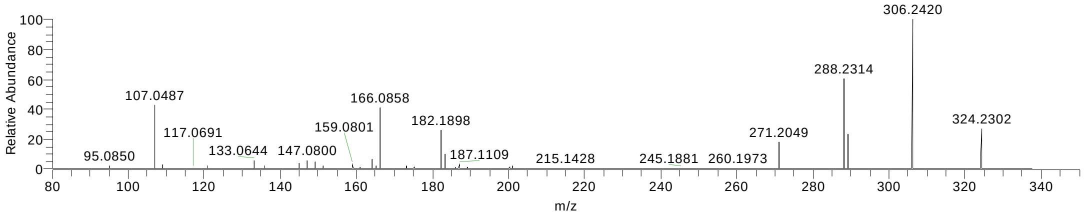

G2

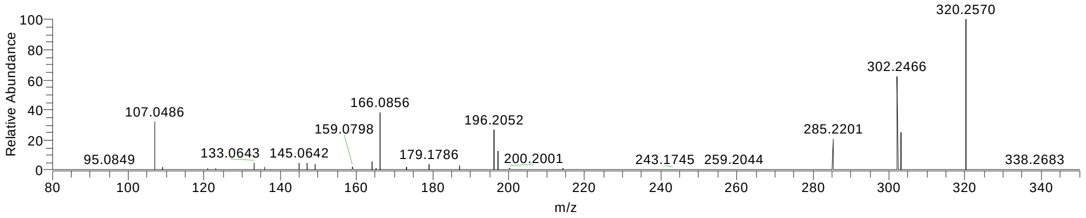

G3

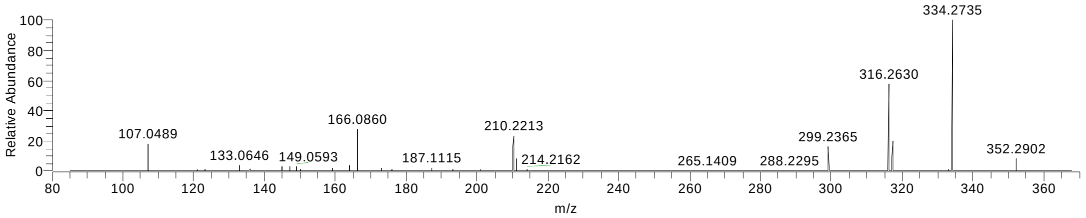

G4

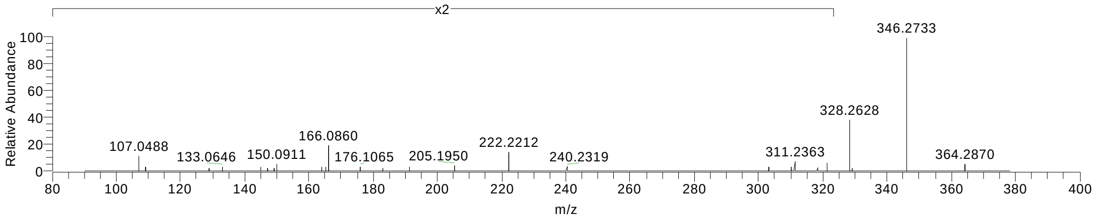

G5

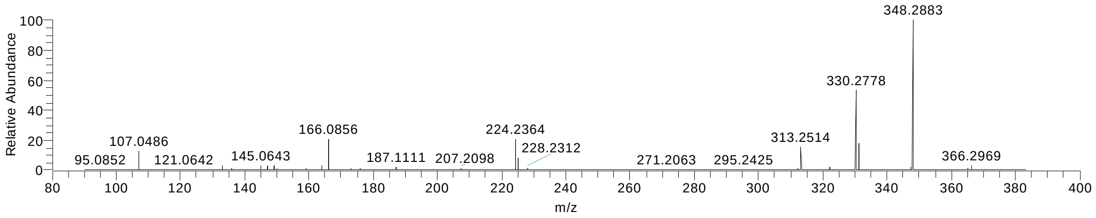

G6

Figure S12. HR-ESIMS/MS spectra of subgroup G

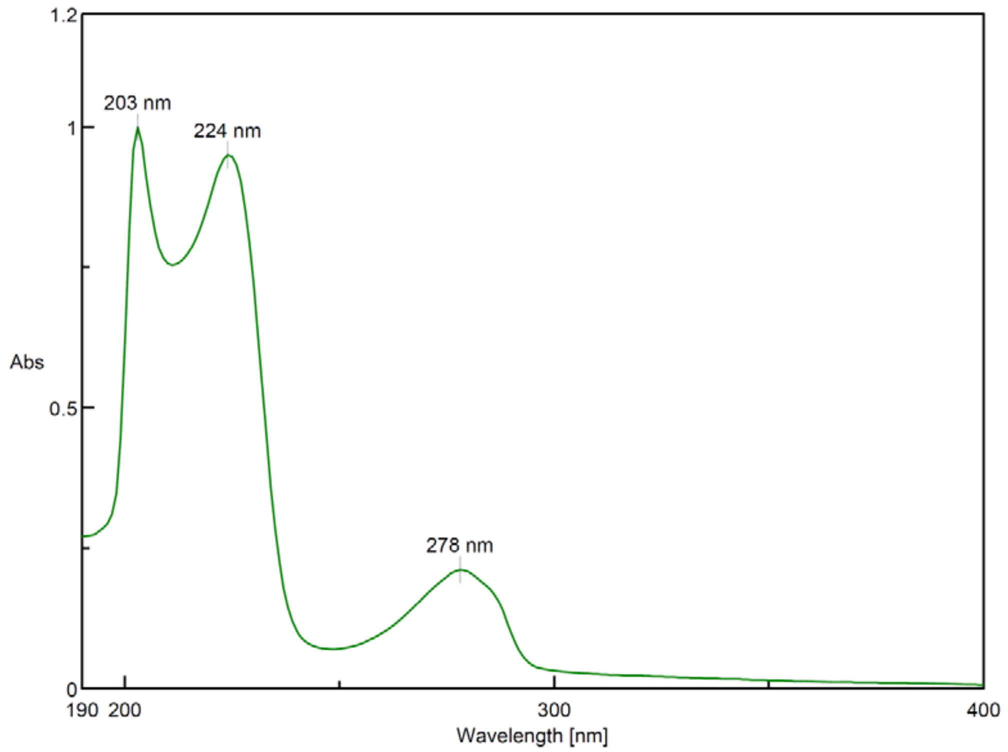

Figure S13. UV spectrum of **1**

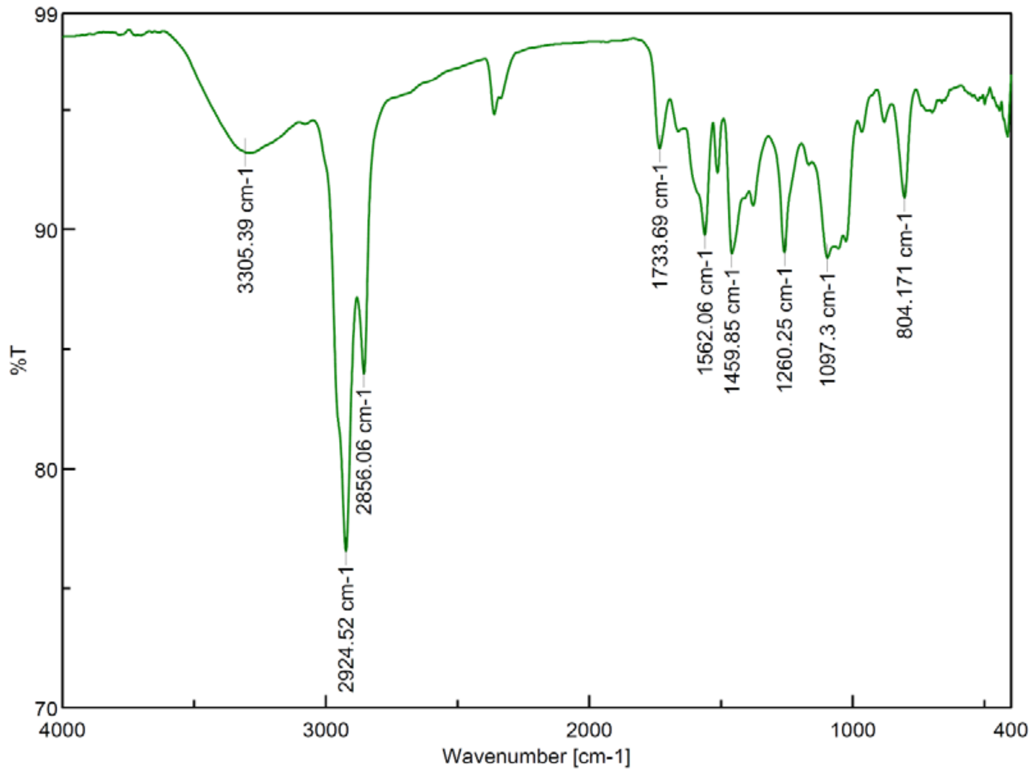

Figure S14. FTIR spectrum of **1**

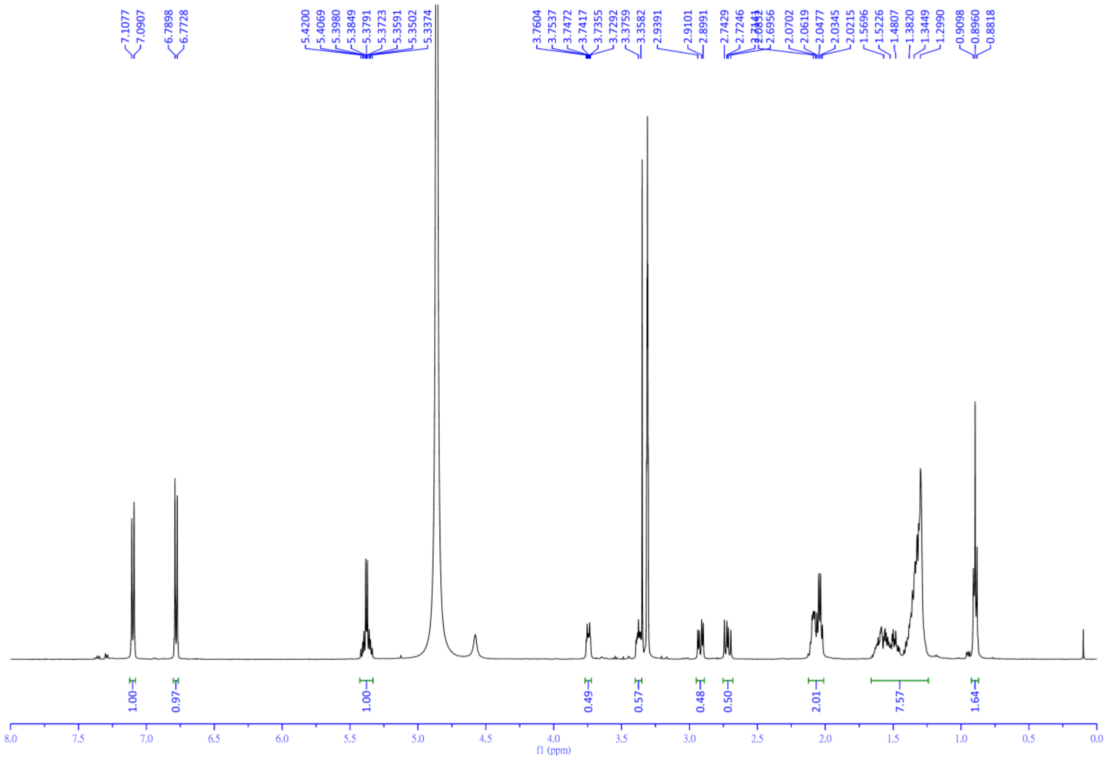

Figure S15. ^{1}H NMR spectrum of **1**

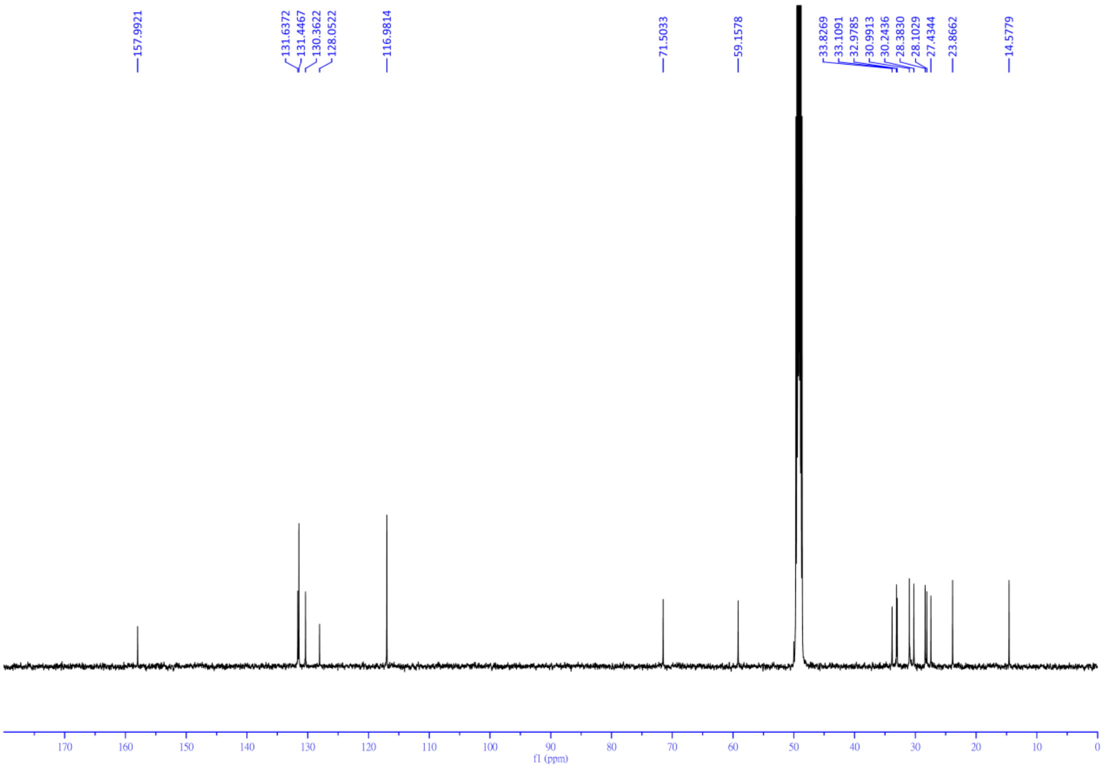

Figure S16. ^{13}C NMR spectrum of **1**

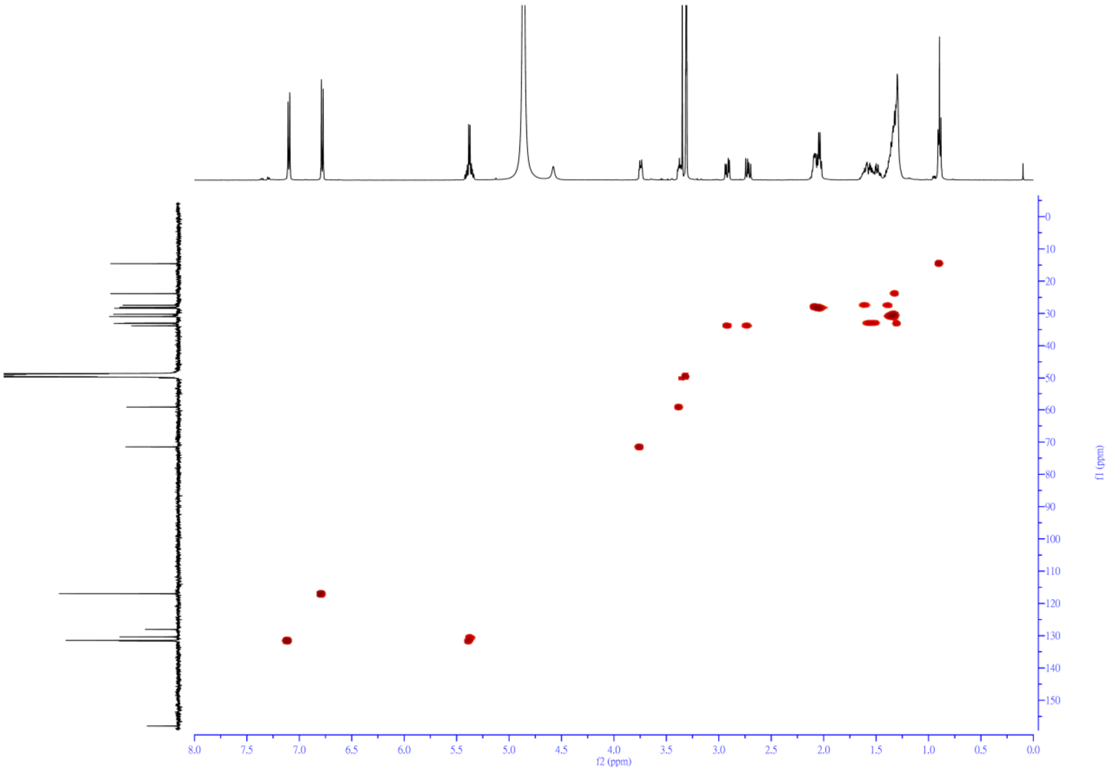

Figure S17. HSQC spectrum of **1**

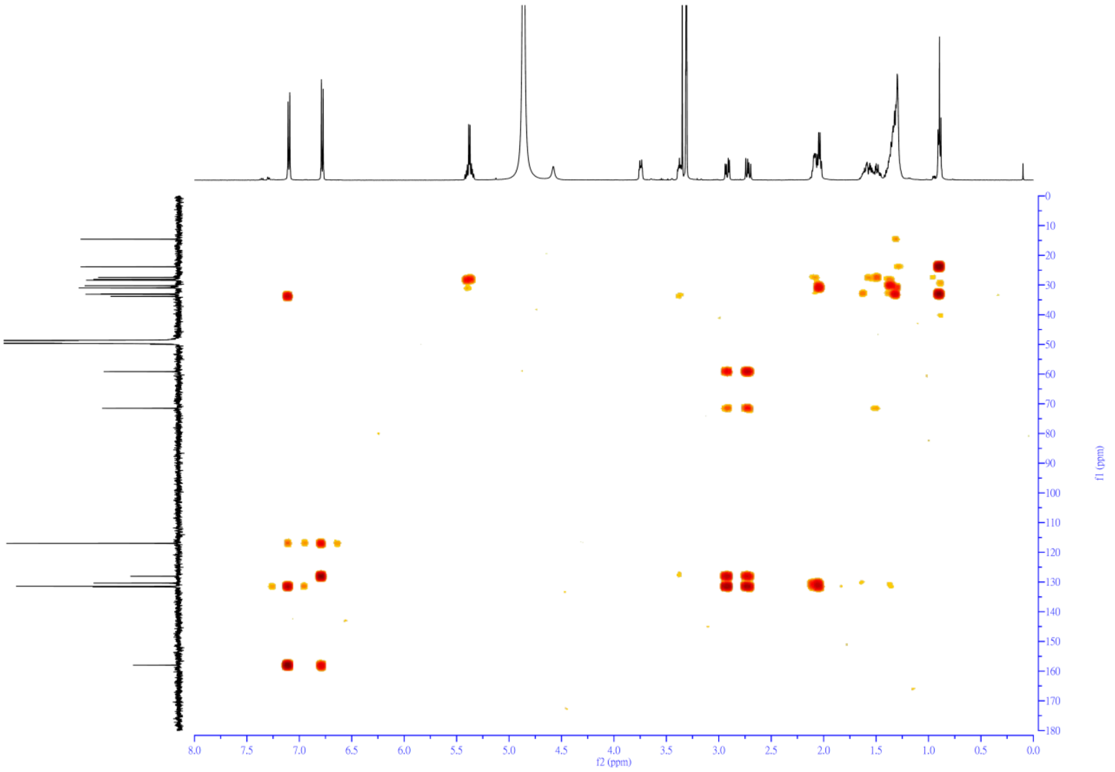

Figure S18. HMBC spectrum of **1**

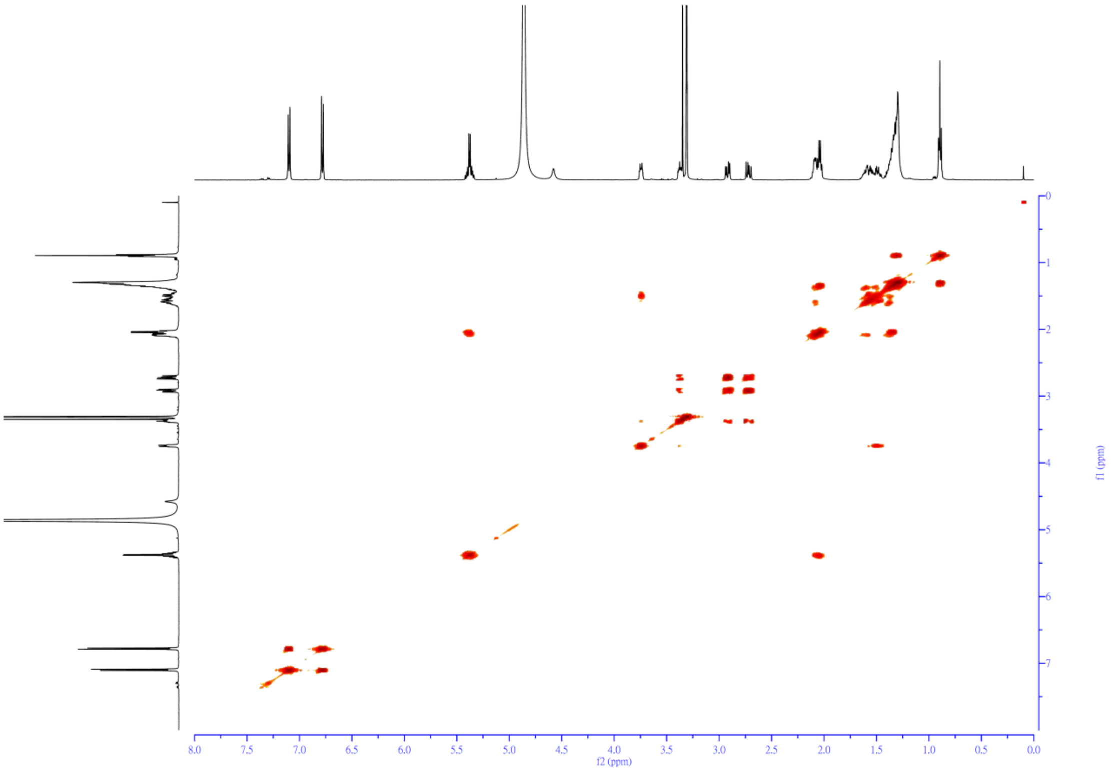

Figure S19. ^{1}H-^{1}H COSY spectrum of **1**

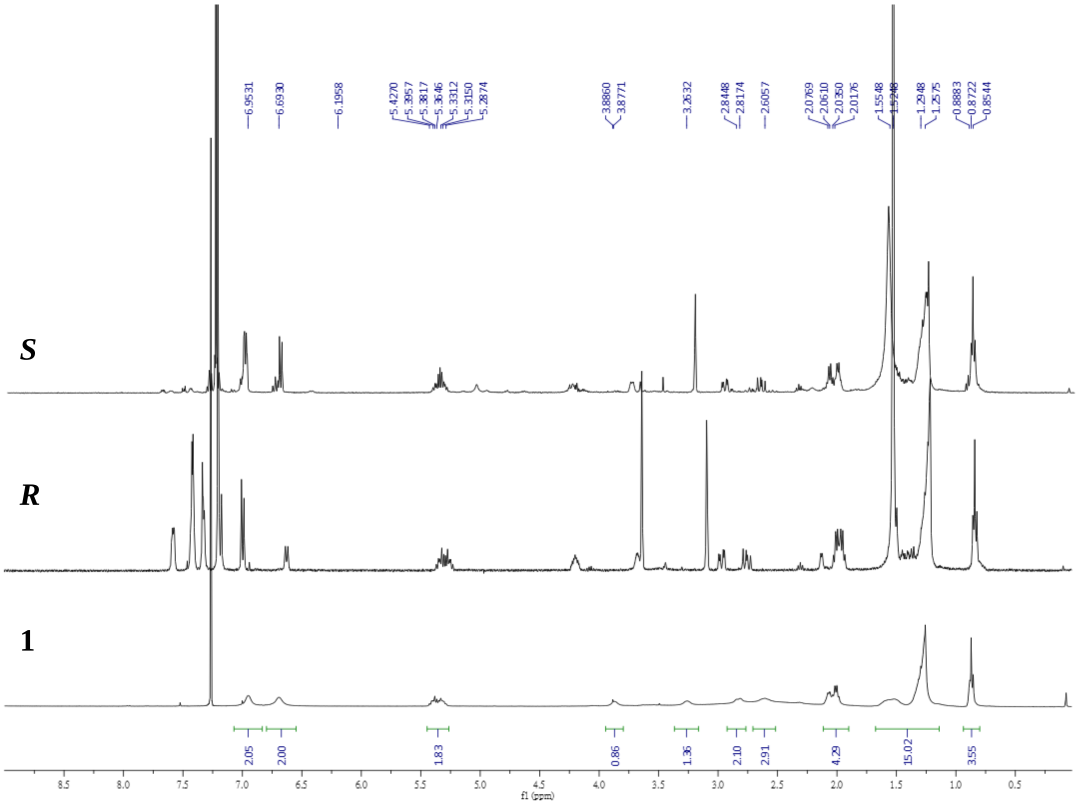

Figure S20. ^{1}H NMR Spectra of **1** and its ***R*** and ***S*** Mosher derivatives in $CDCl_3$

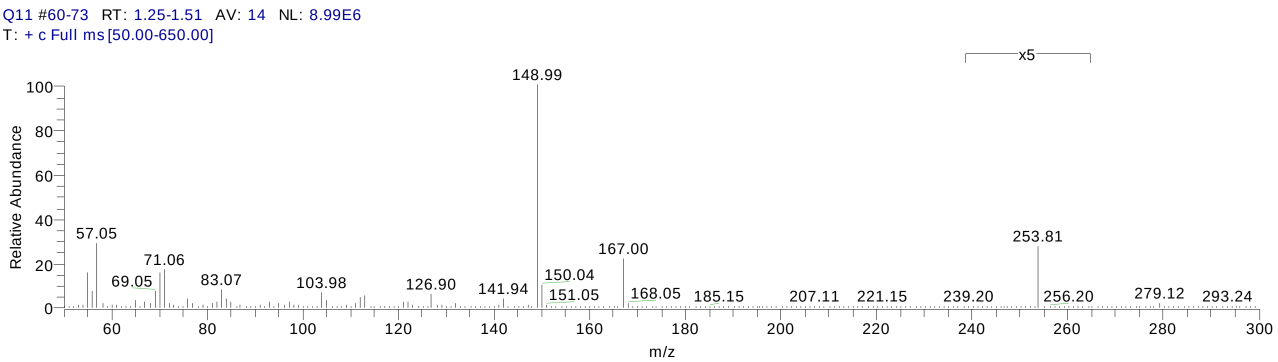

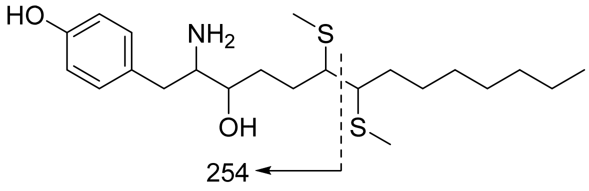

Figure S21. EIMS spectrum and fragmentation of the dimethyl disulfide derivative of **1**

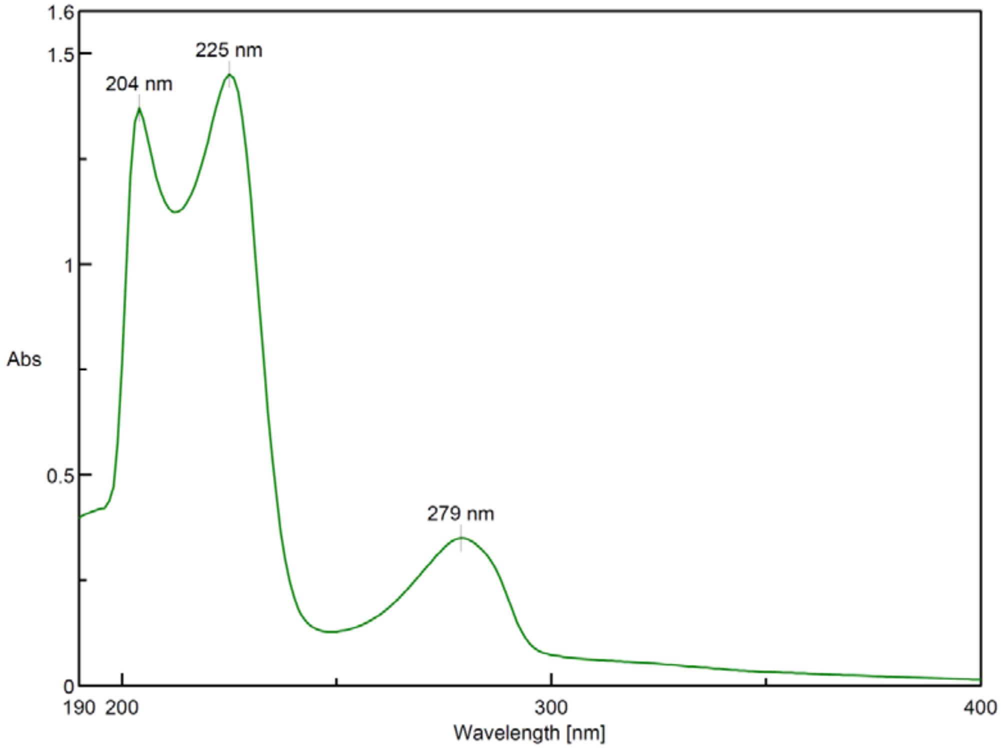

Figure S22. UV spectrum of **2**

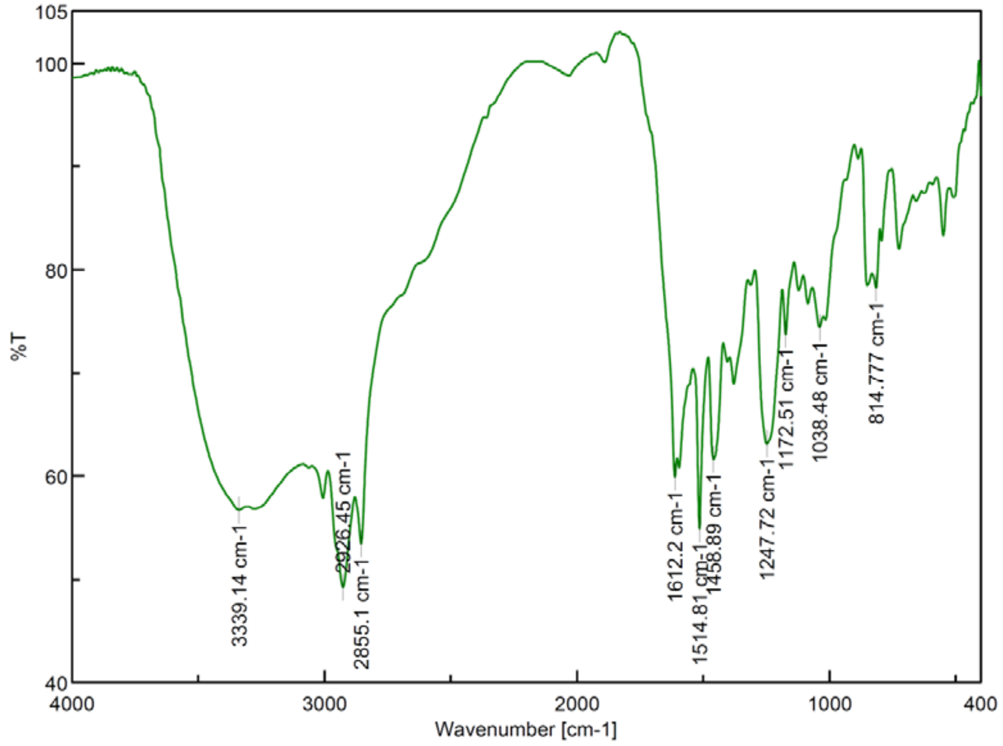

Figure S23. FTIR spectrum of **2**

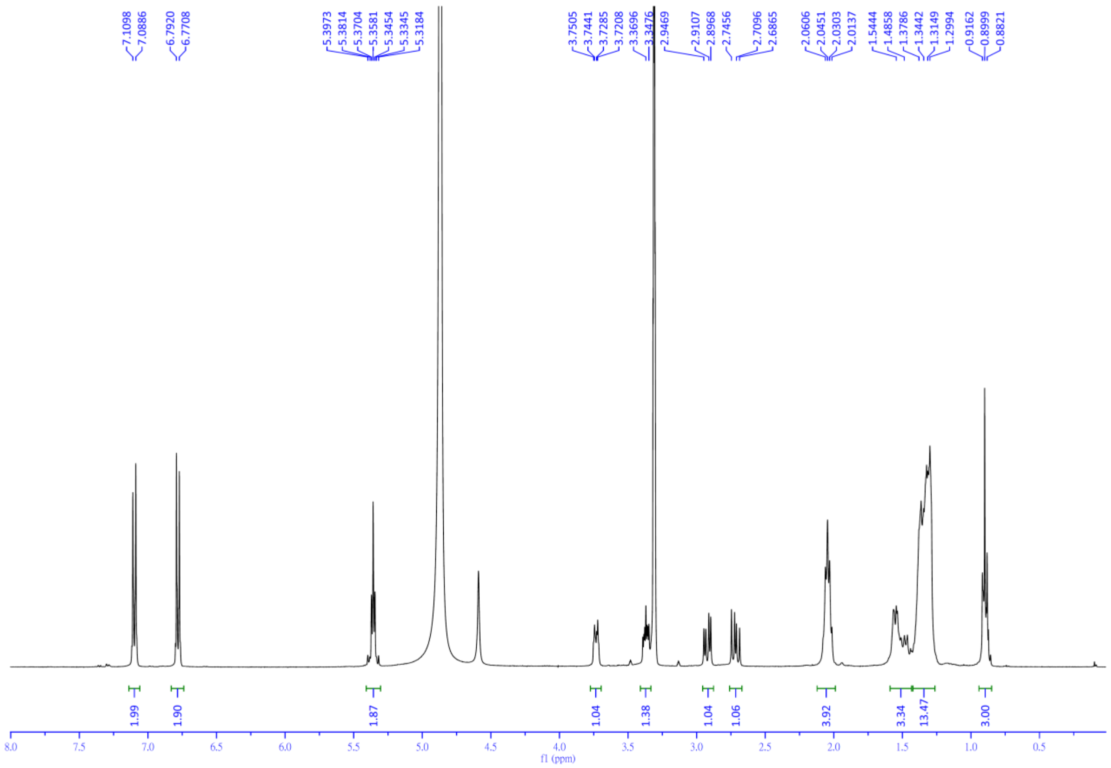

Figure S24. ^{1}H NMR spectrum of **2**

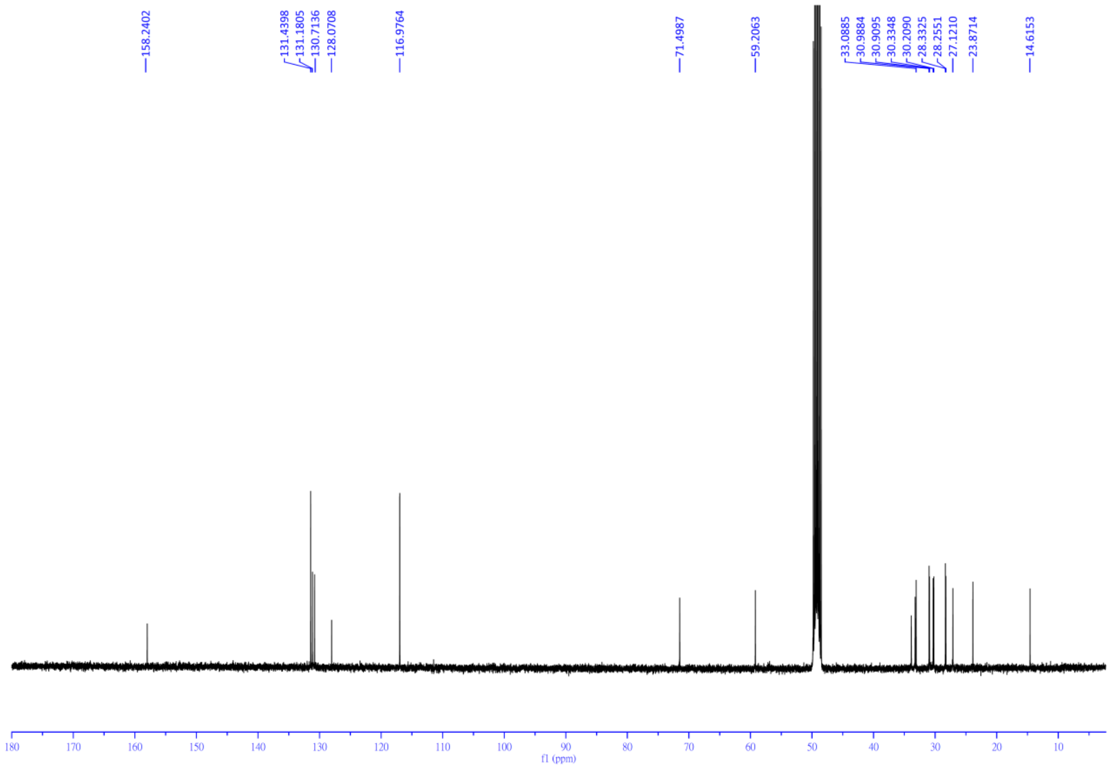

Figure S25. ^{13}C NMR spectrum of **2**

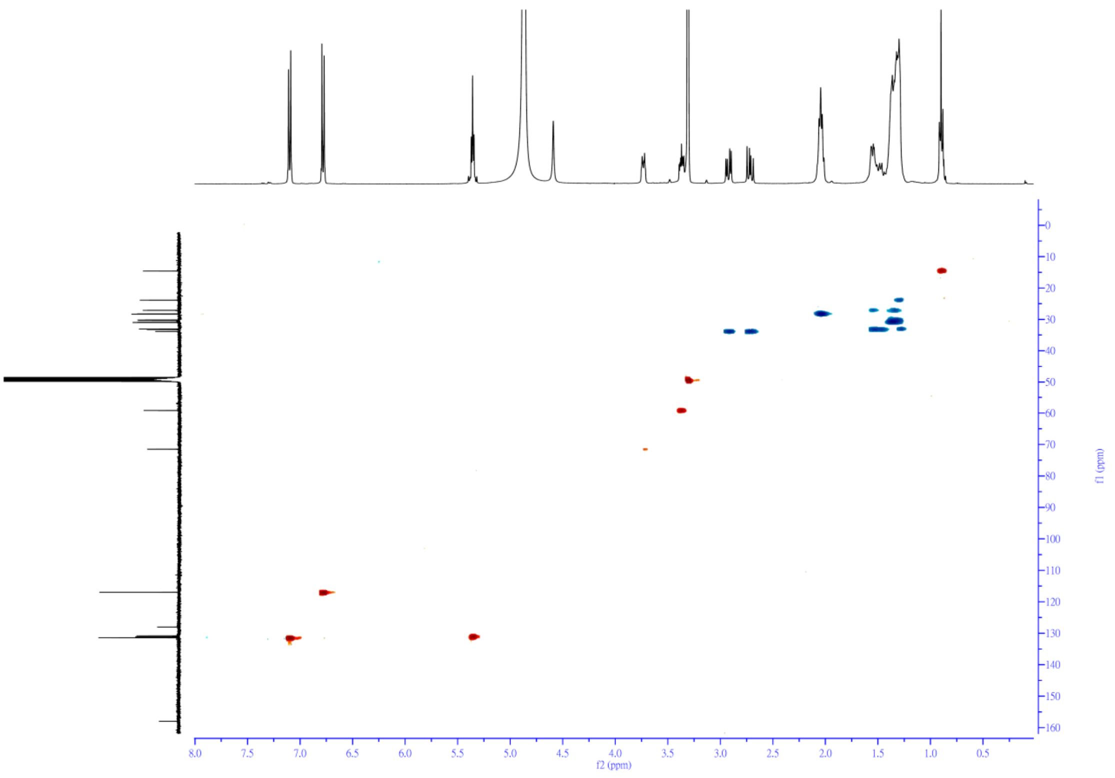

Figure S26. HSQC spectrum of **2**

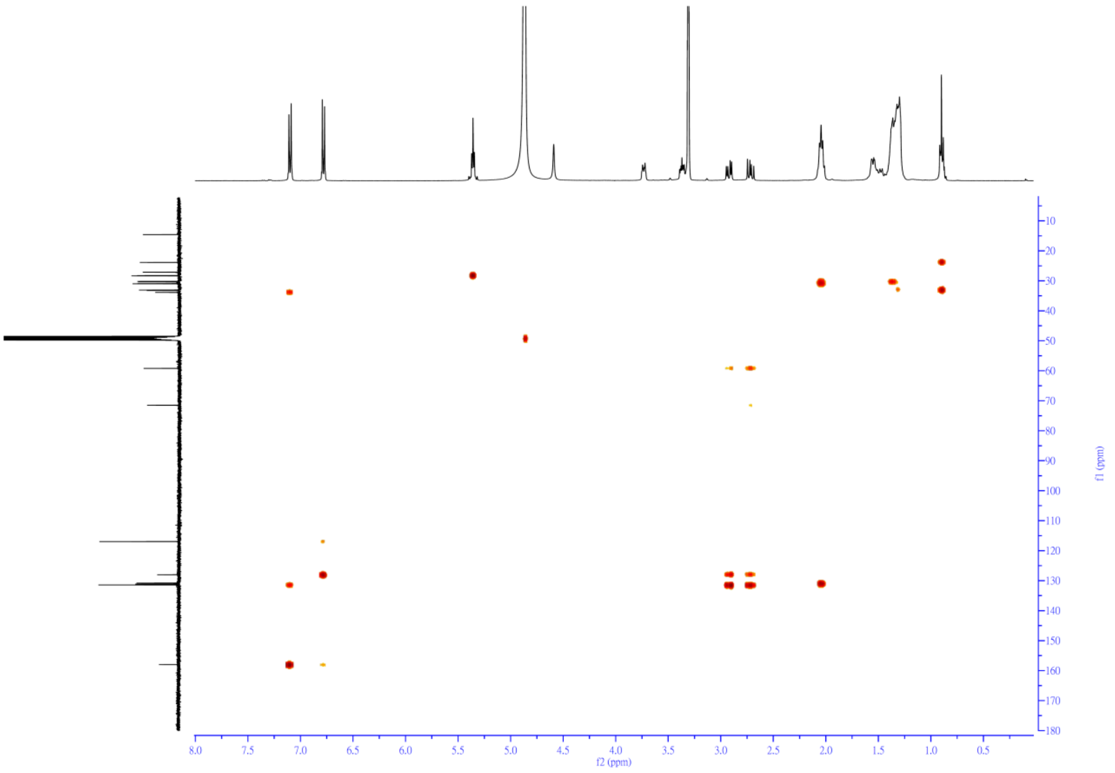

Figure S27. HMBC spectrum of **2**

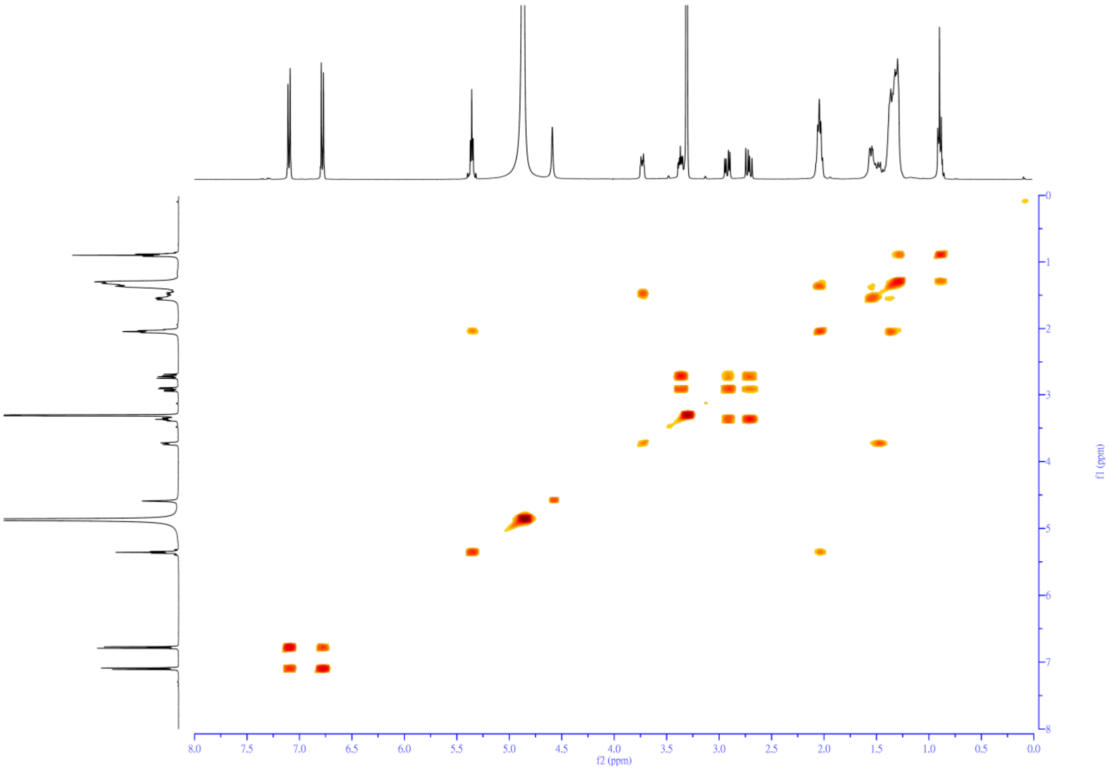

Figure S28. ^{1}H-^{1}H COSY spectrum of **2**

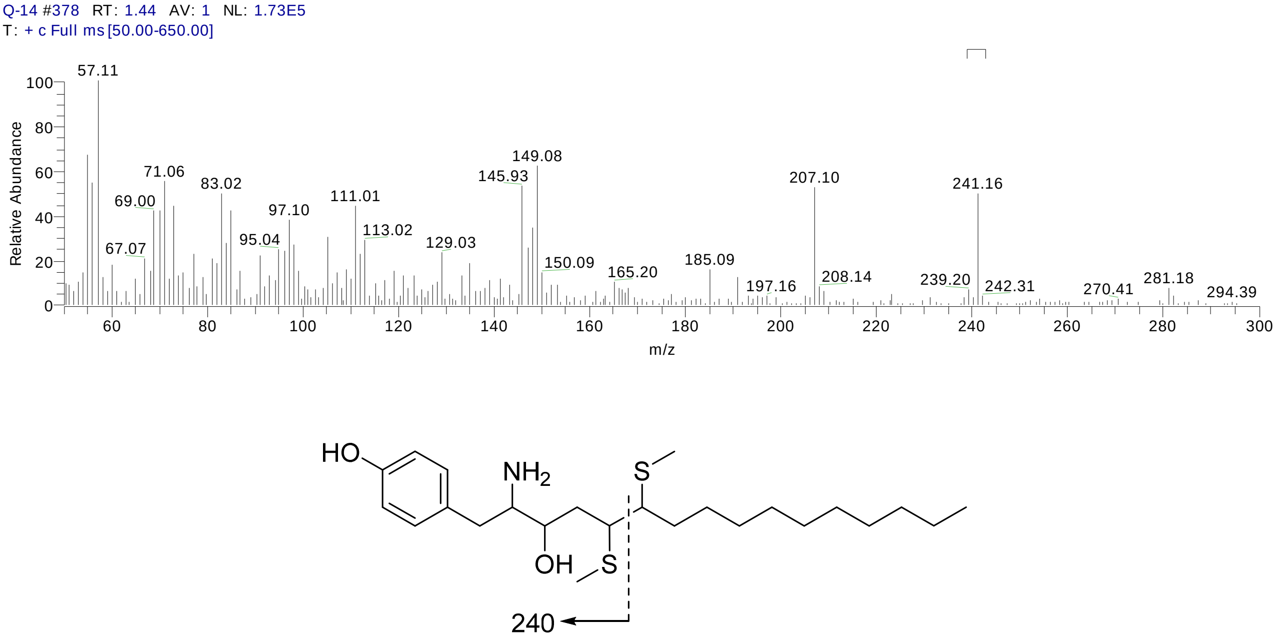

Figure S29. EIMS spectrum and fragmentation of the dimethyl disulfide derivative of **2**

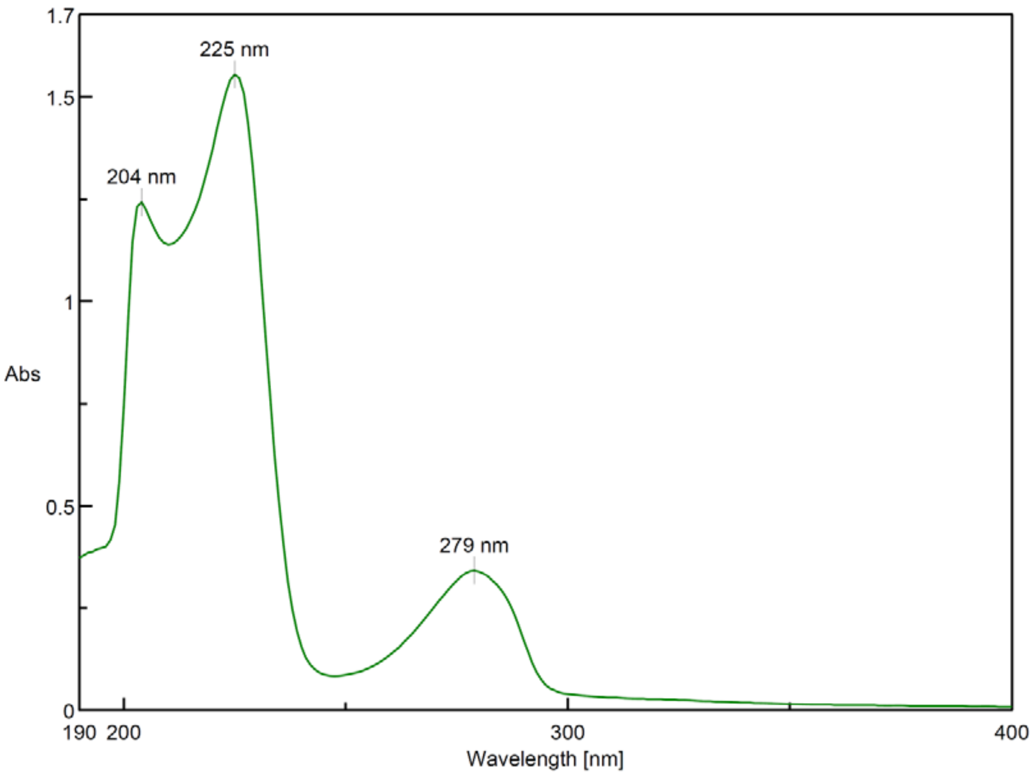

Figure S30. UV spectrum of **3**

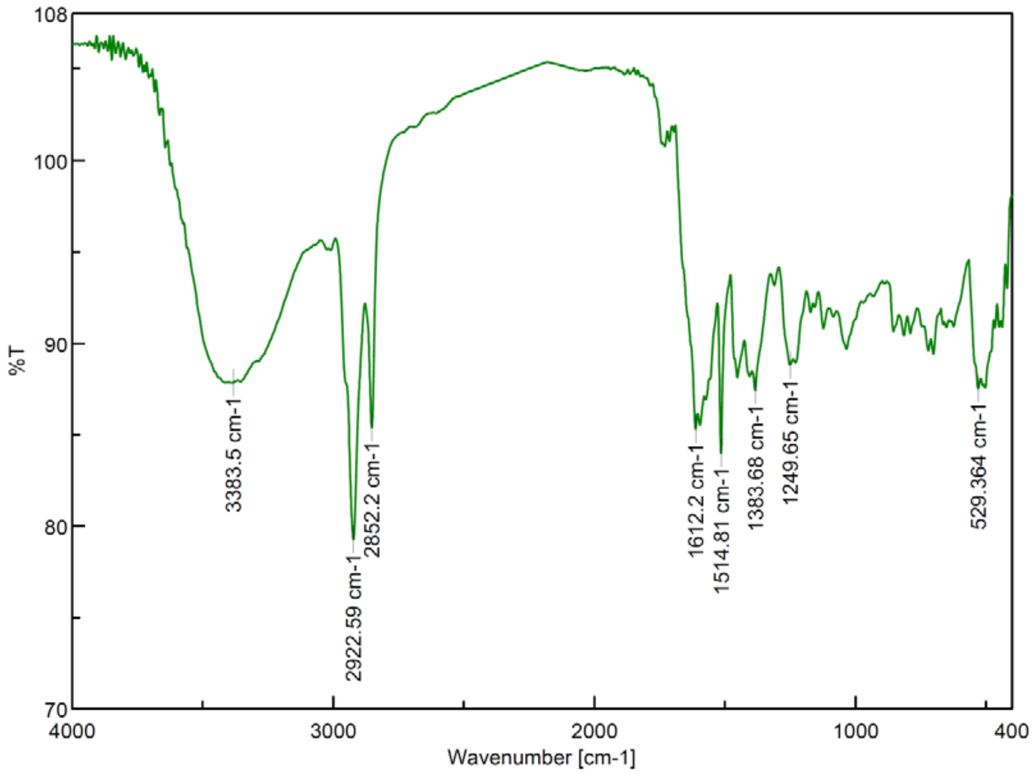

Figure S31. FTIR spectrum of **3**

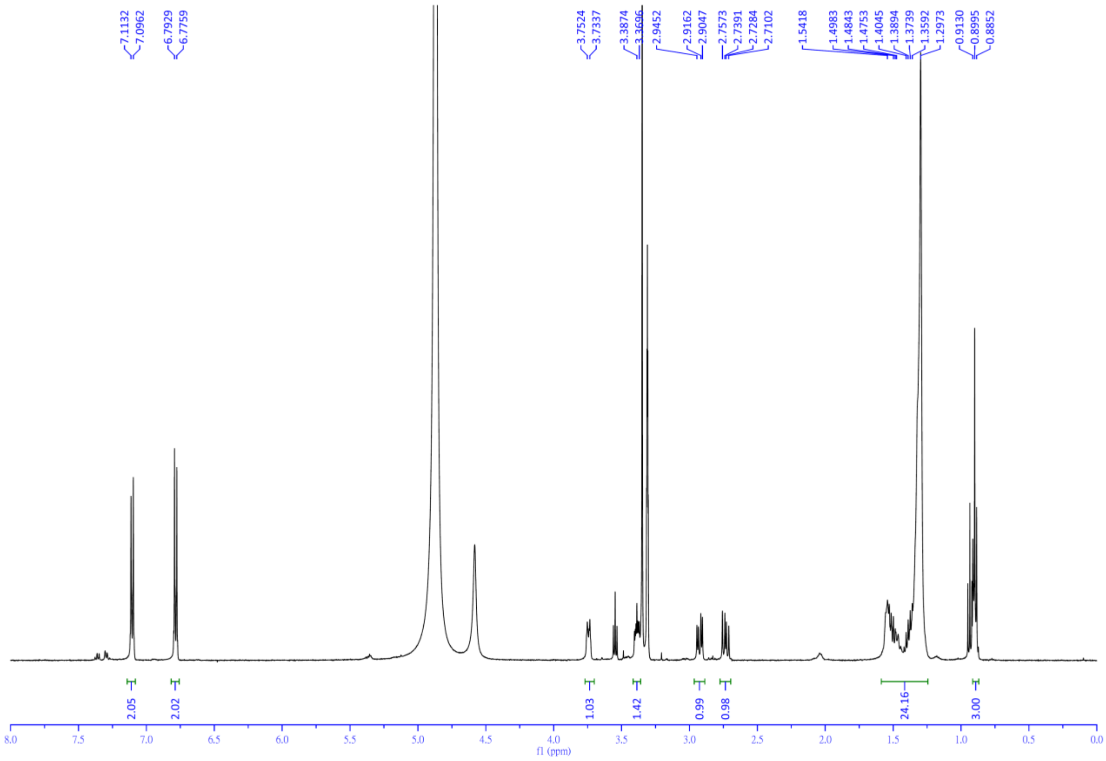

Figure S32. ^{1}H NMR spectrum of **3**

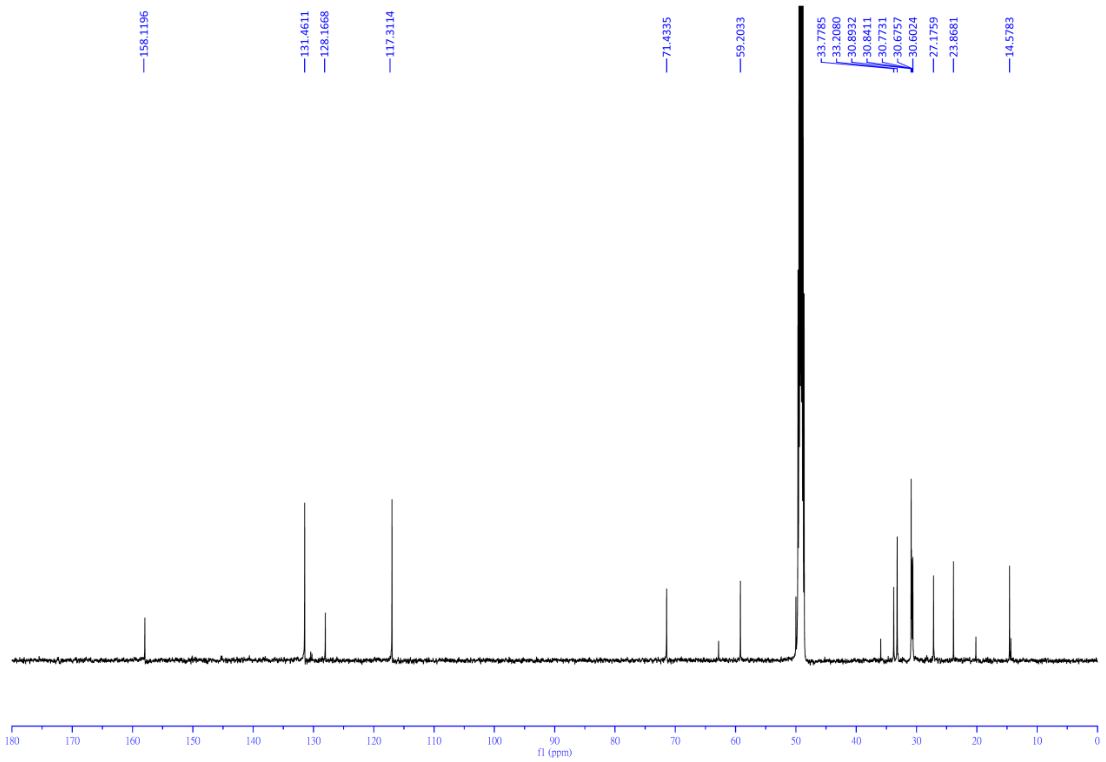

Figure S33. ^{13}C NMR spectrum of **3**

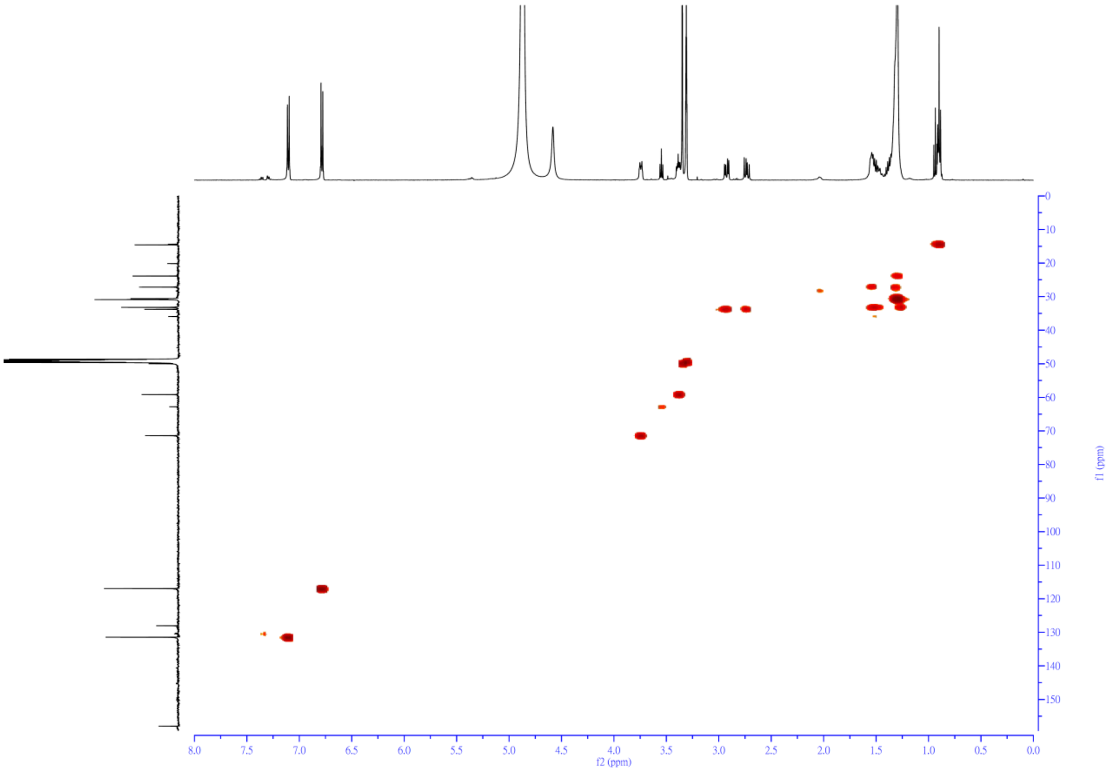

Figure S34. HSQC spectrum of **3**

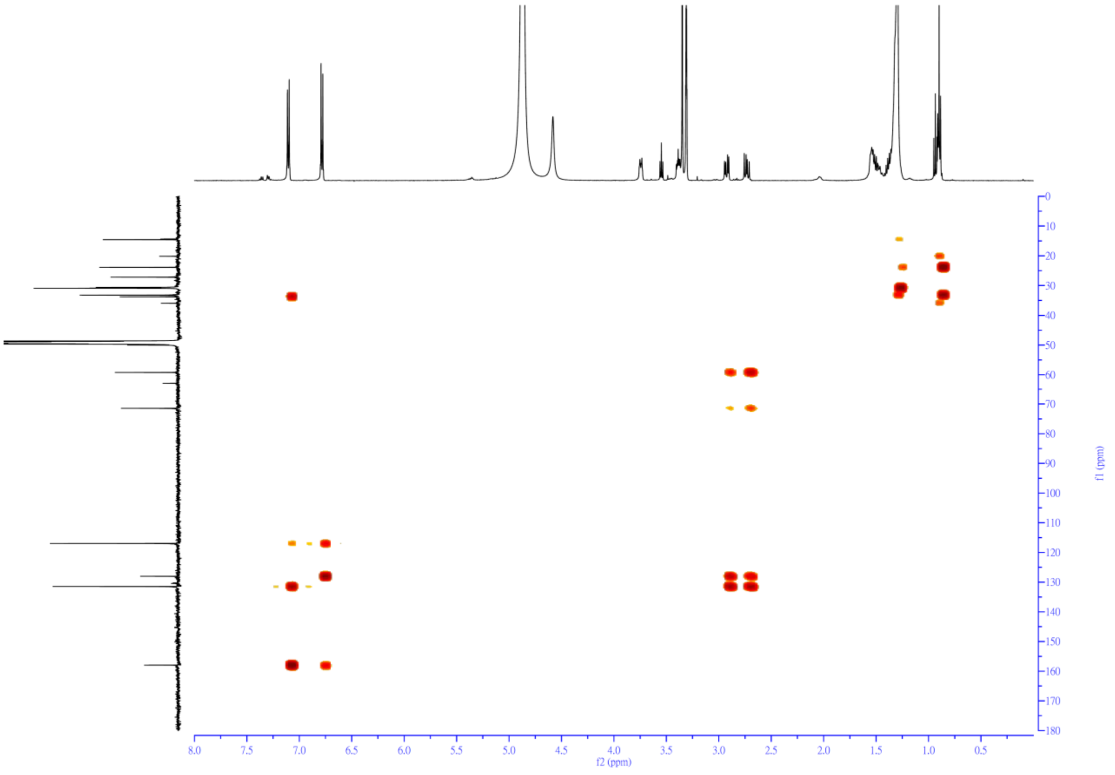

Figure S35. HMBC spectrum of **3**

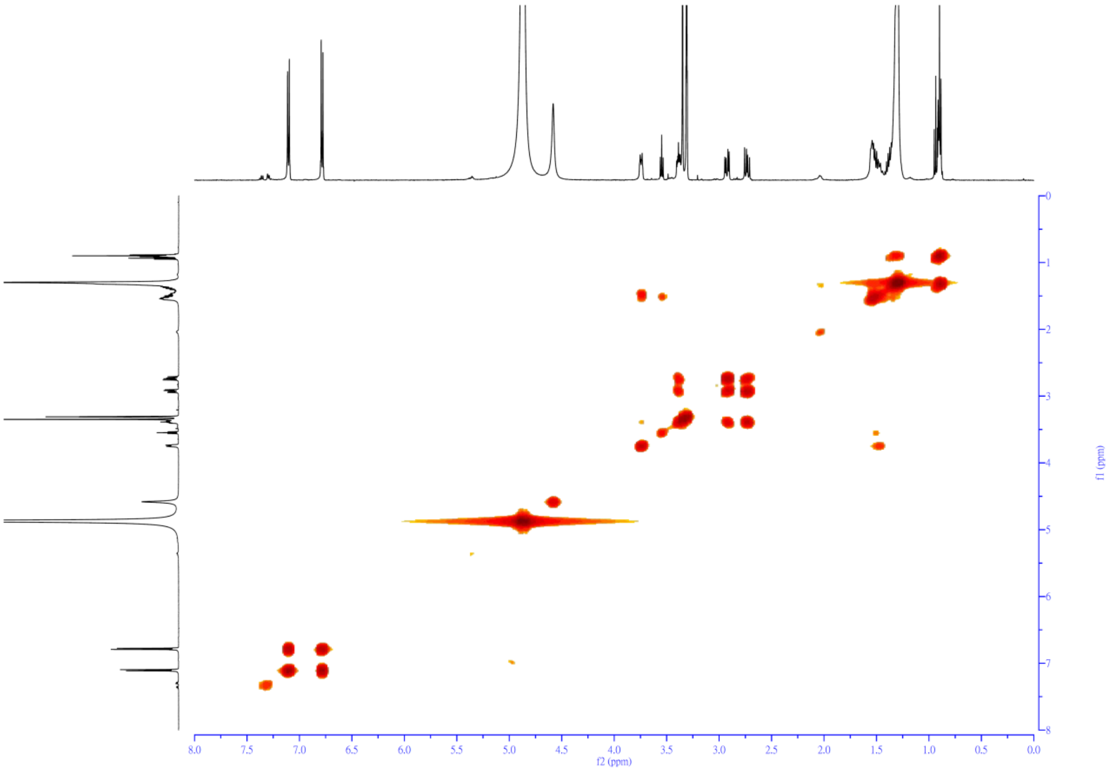

Figure S36. ^{1}H-^{1}H COSY spectrum of **3**

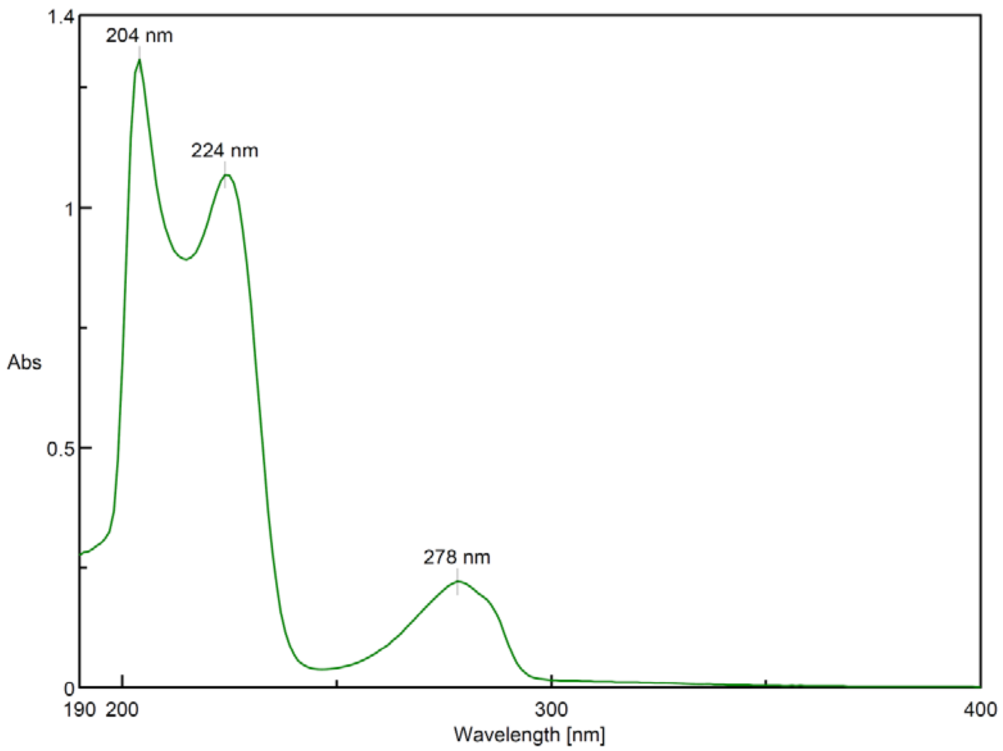

Figure S37. UV spectrum of **4**

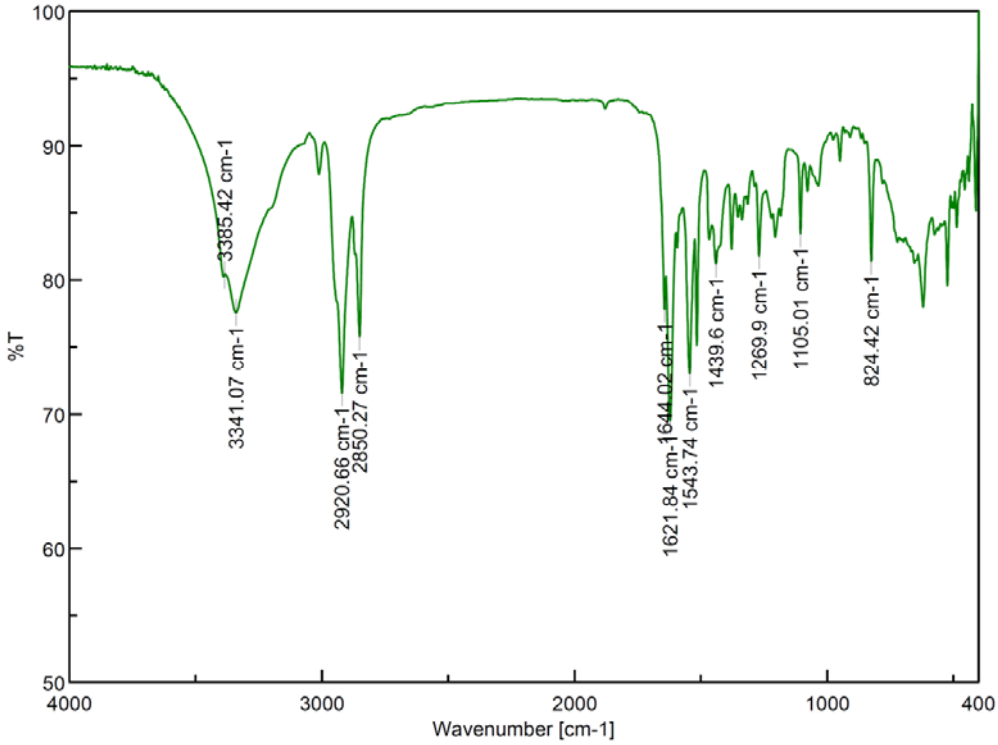

Figure S38. FTIR spectrum of **4**

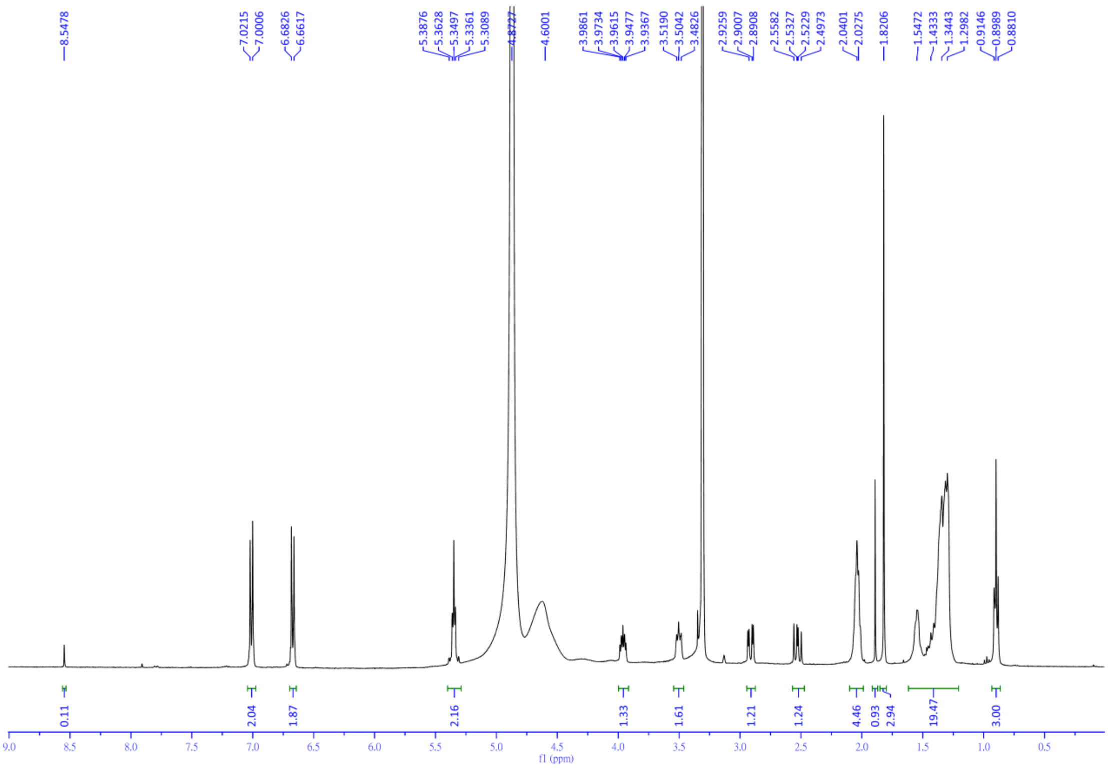

Figure S39. ^{1}H NMR spectrum of **4**

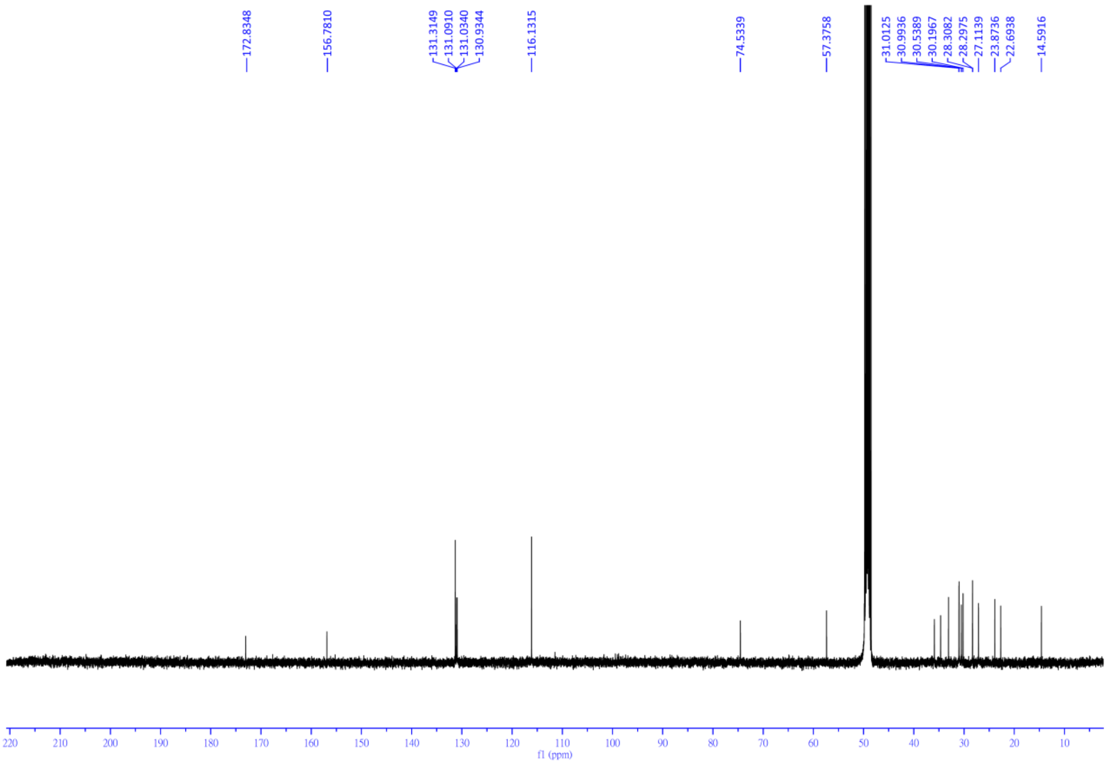

Figure S40. ^{13}C NMR spectrum of **4**

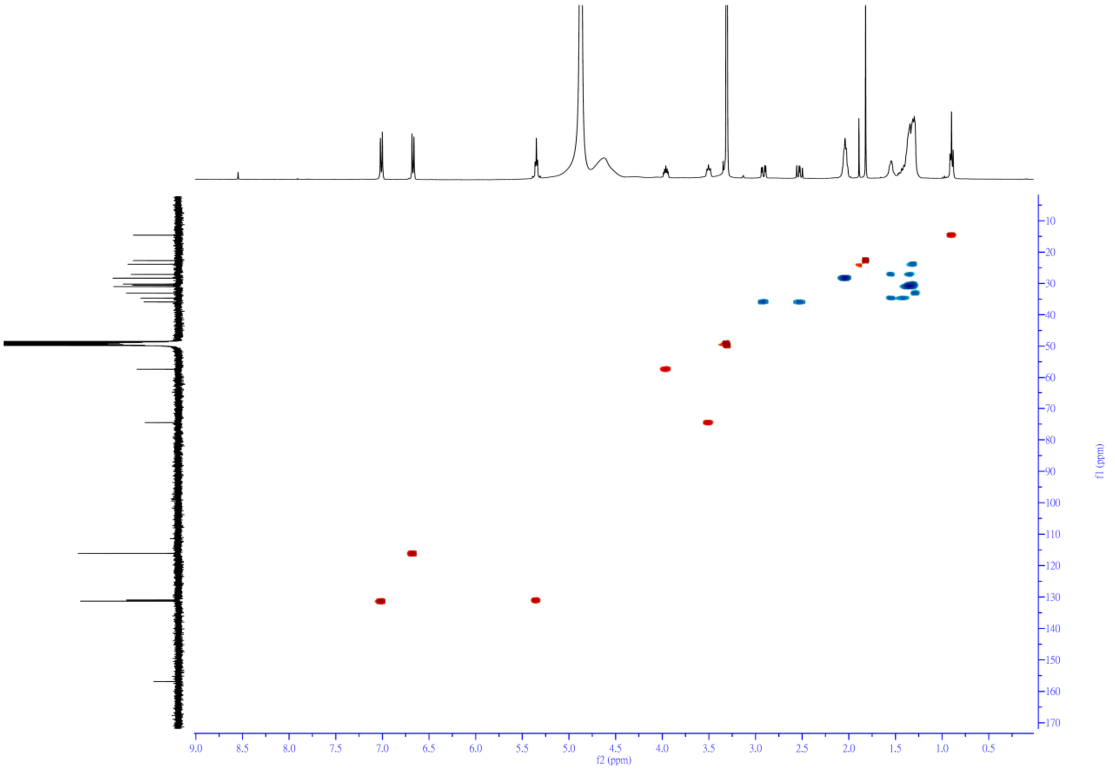

Figure S41. HSQC spectrum of **4**

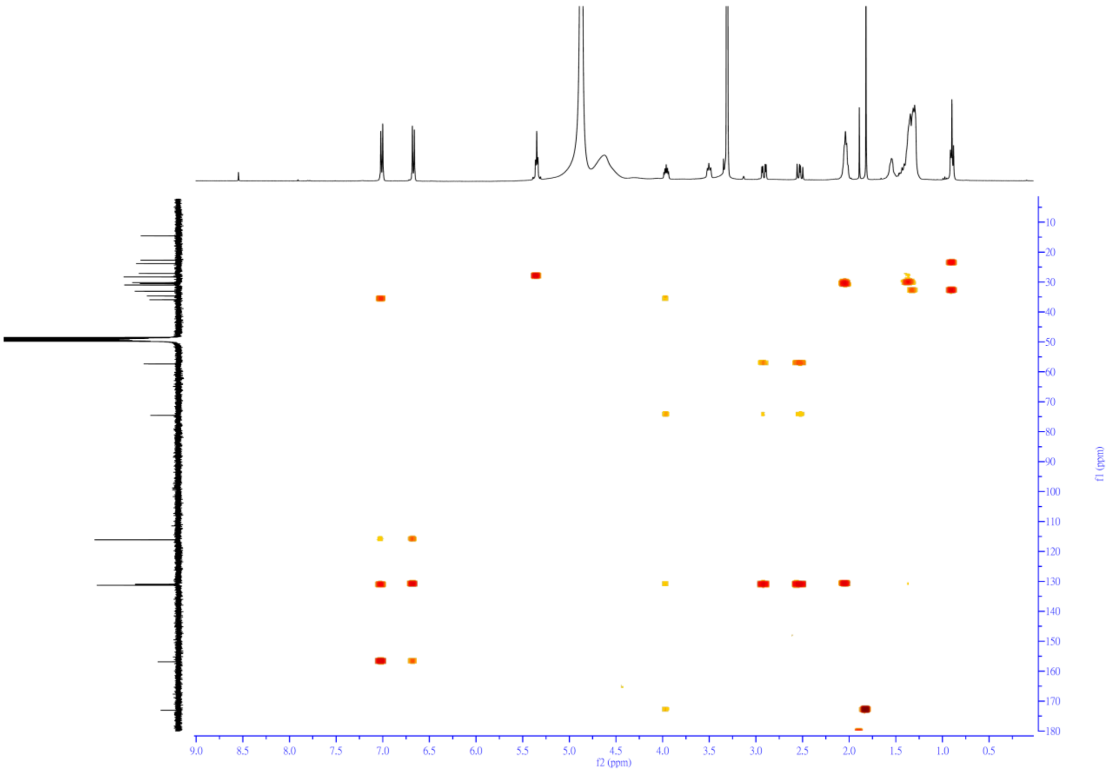

Figure S42. HMBC spectrum of **4**

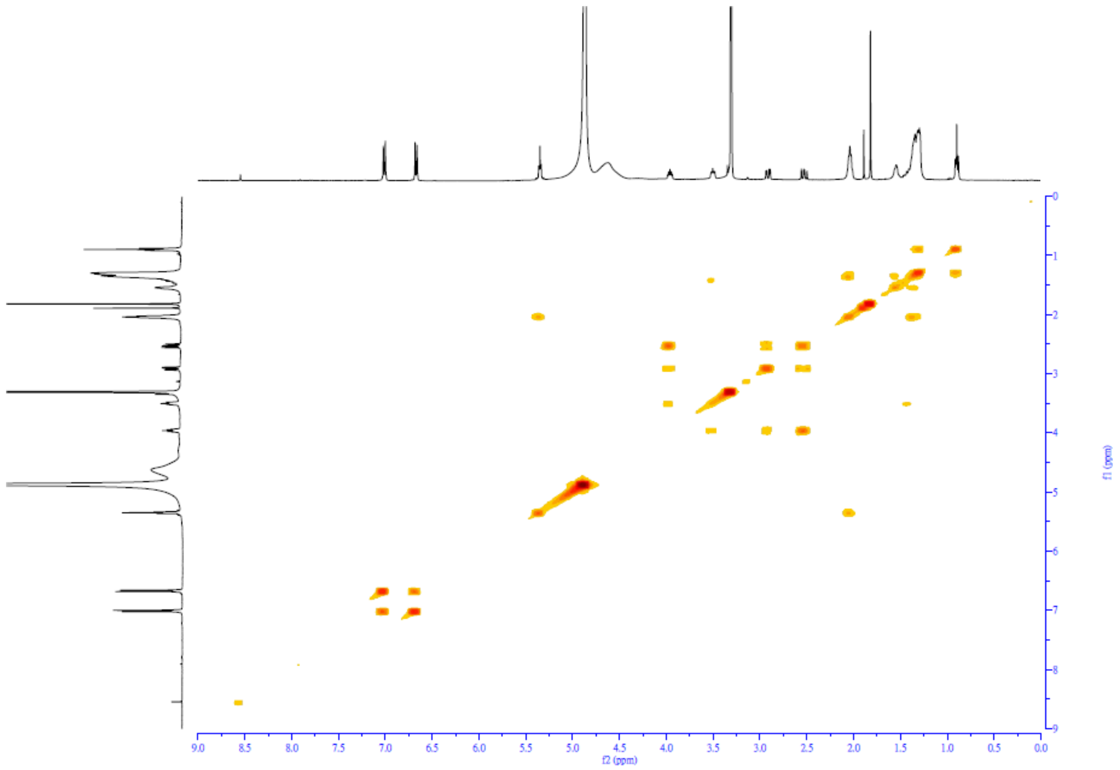

Figure S43. ^{1}H-^{1}H COSY spectrum of **4**

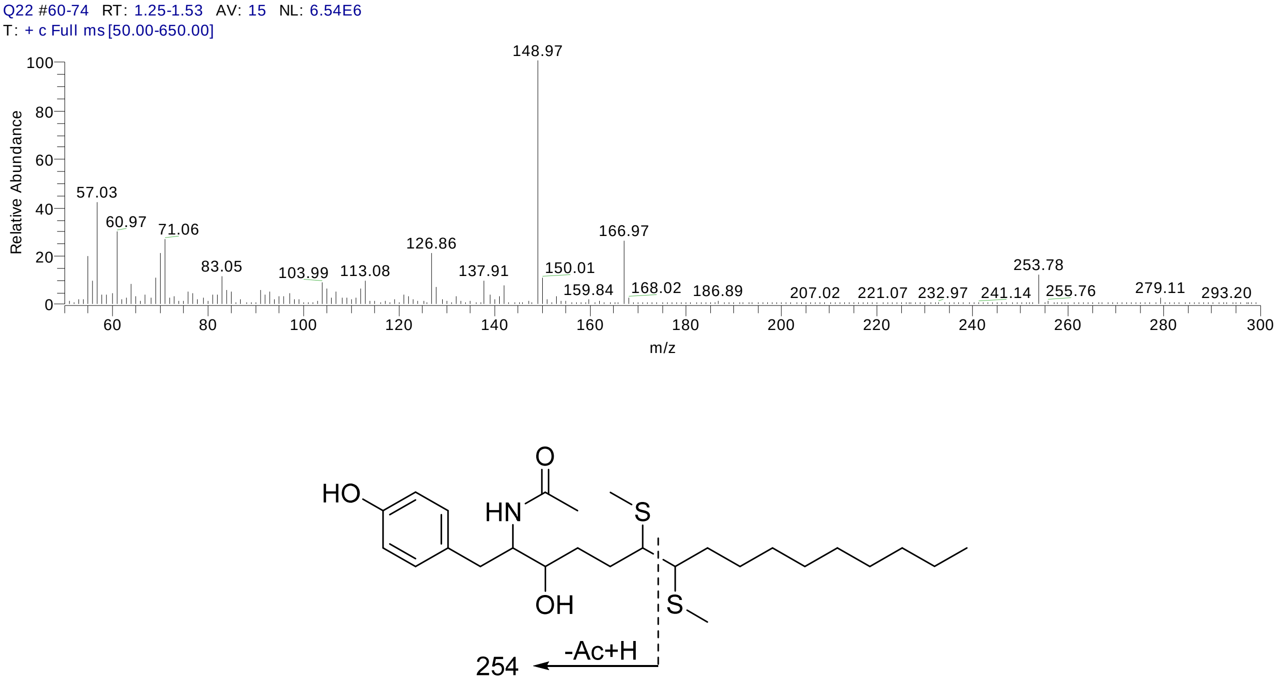

Figure S44. EIMS spectrum and fragmentation of the dimethyl disulfide derivative of **4**

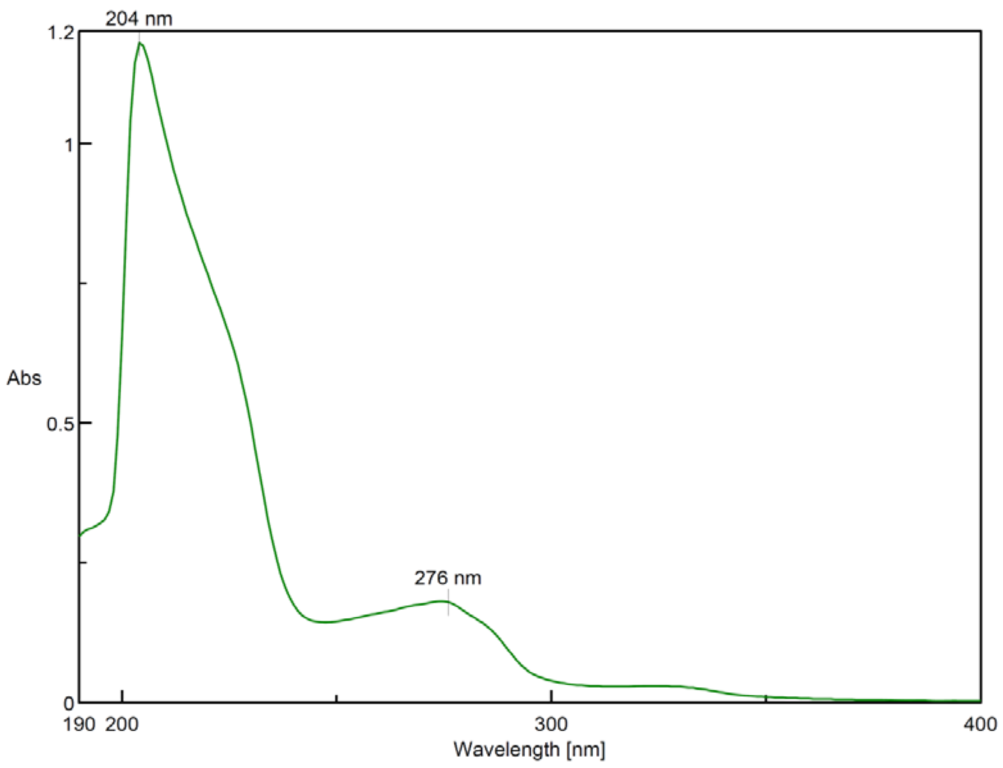

Figure S45. UV spectrum of **5**

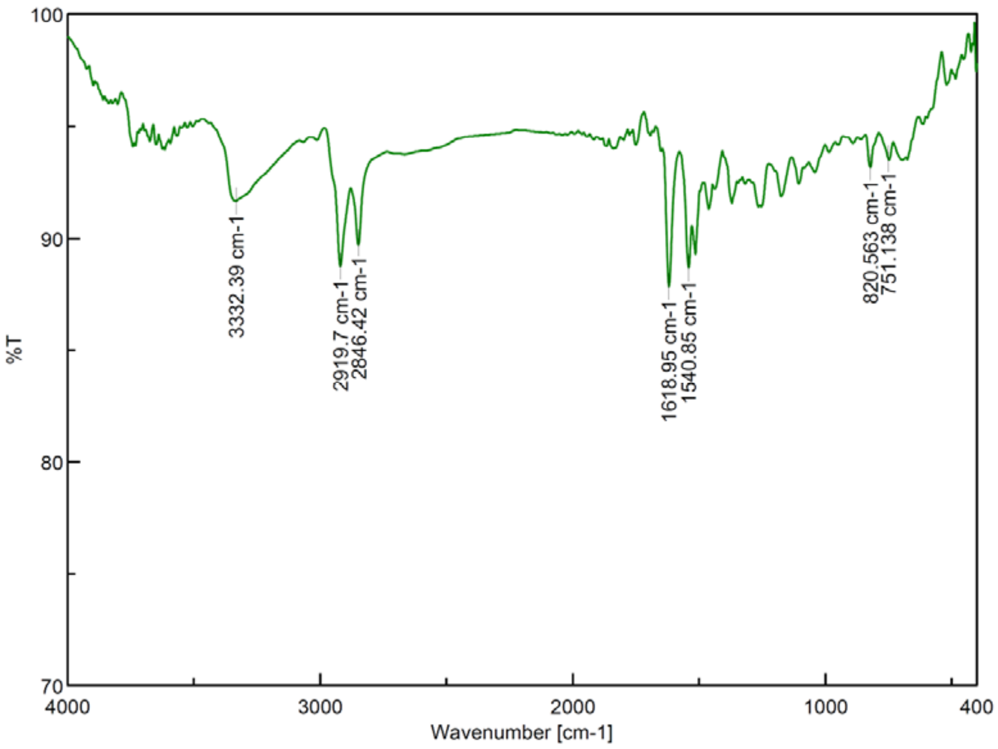

Figure S46. FTIR spectrum of **5**

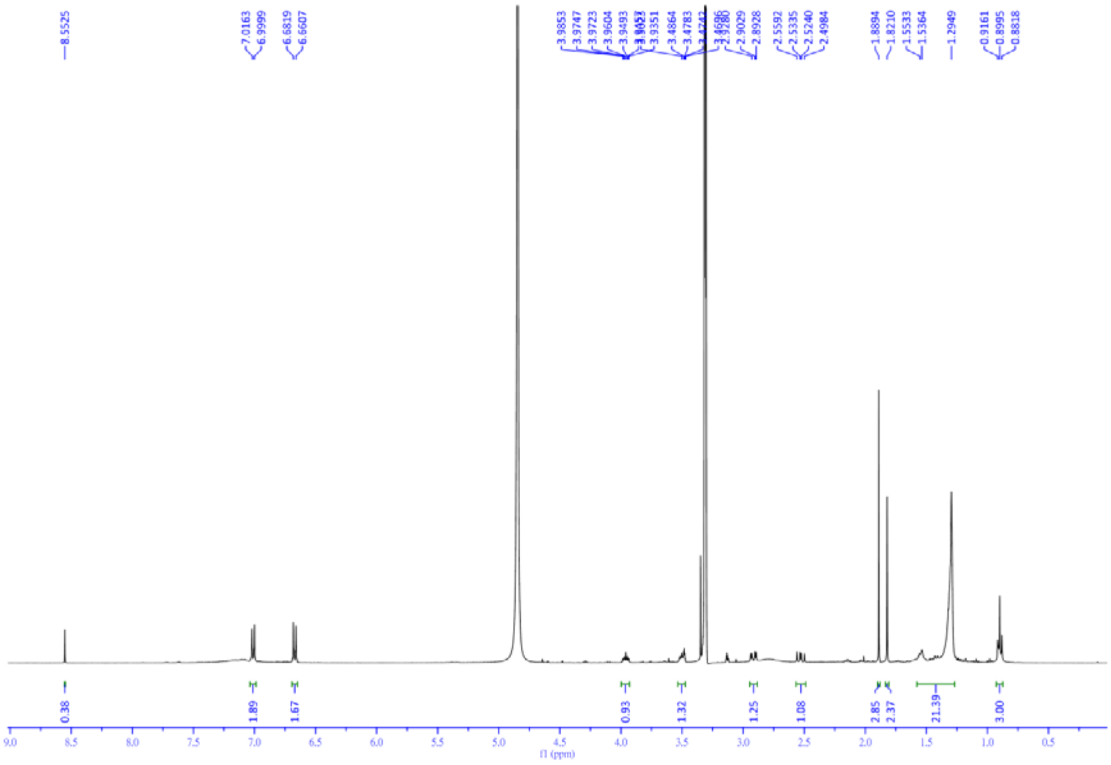

Figure S47. ^{1}H NMR spectrum of **5**

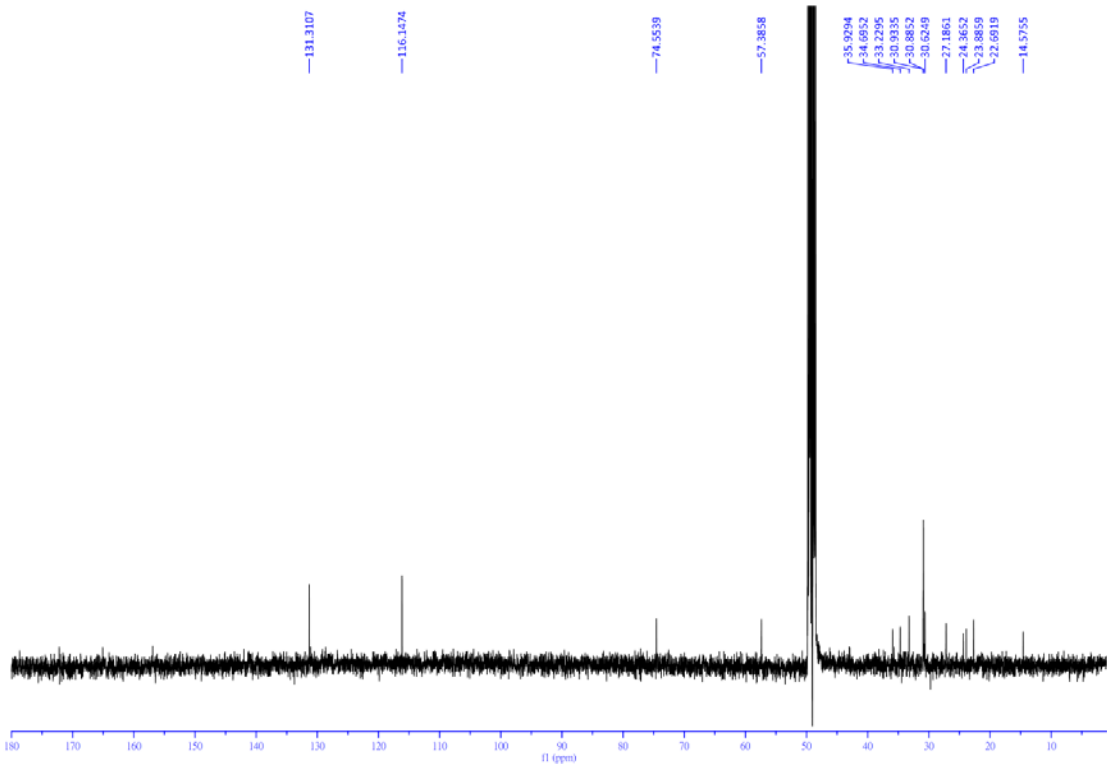

Figure S48. ^{13}C NMR spectrum of **5**

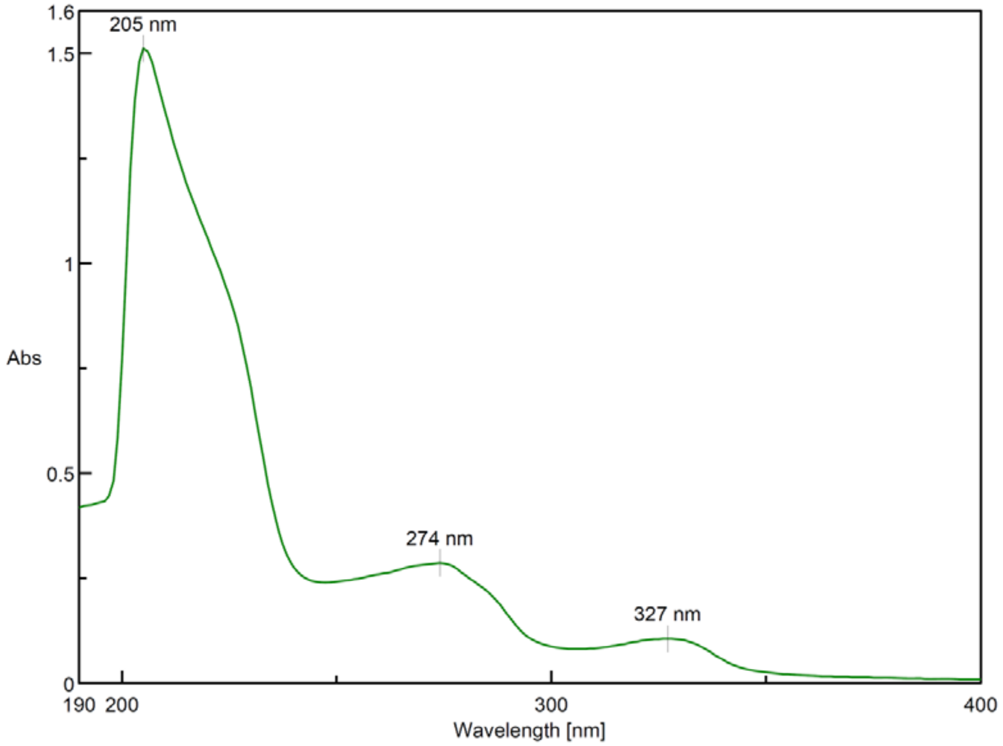

Figure S49. UV spectrum of **6**

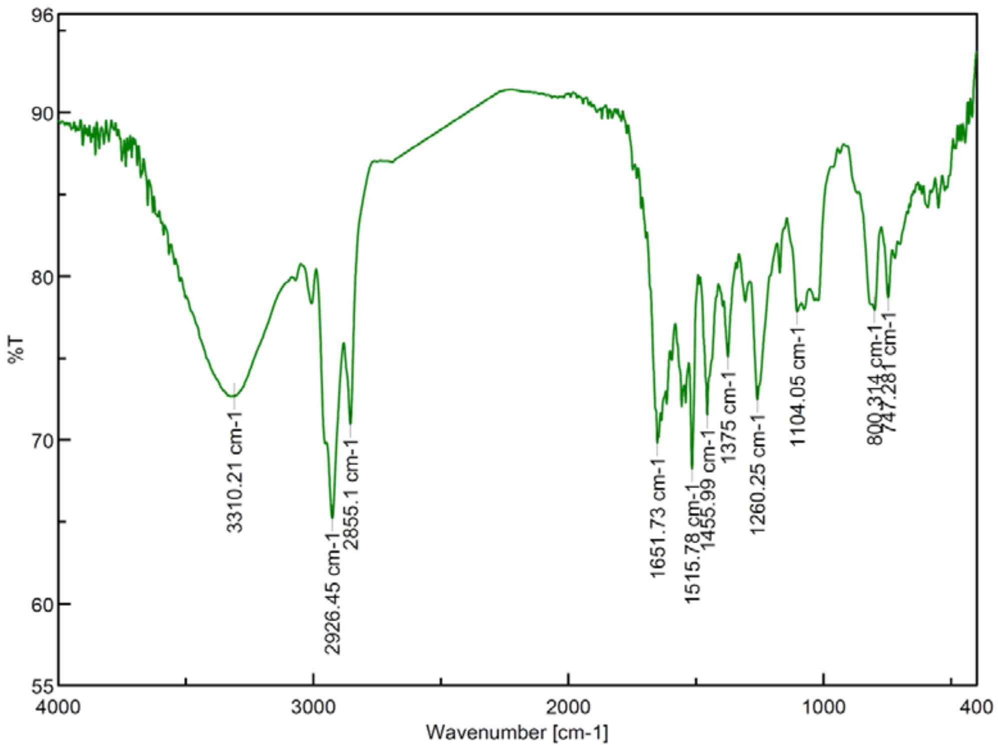

Figure S50. FTIR spectrum of **6**

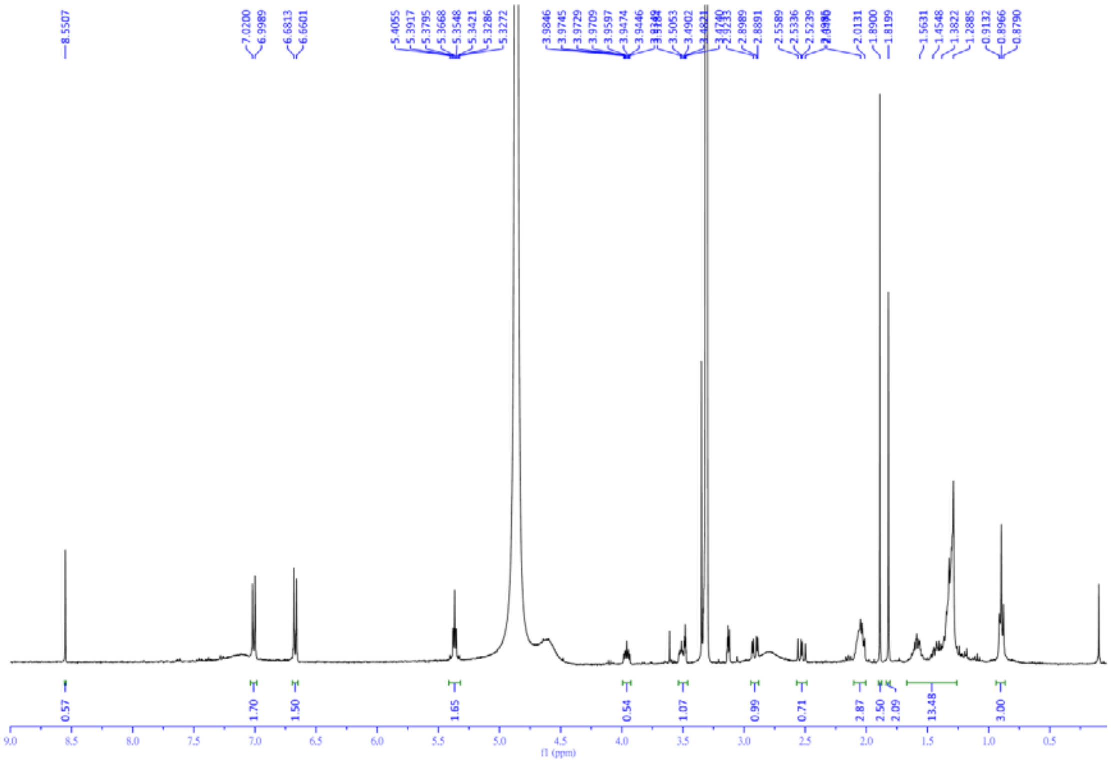

Figure S51. ^{1}H NMR spectrum of **6**

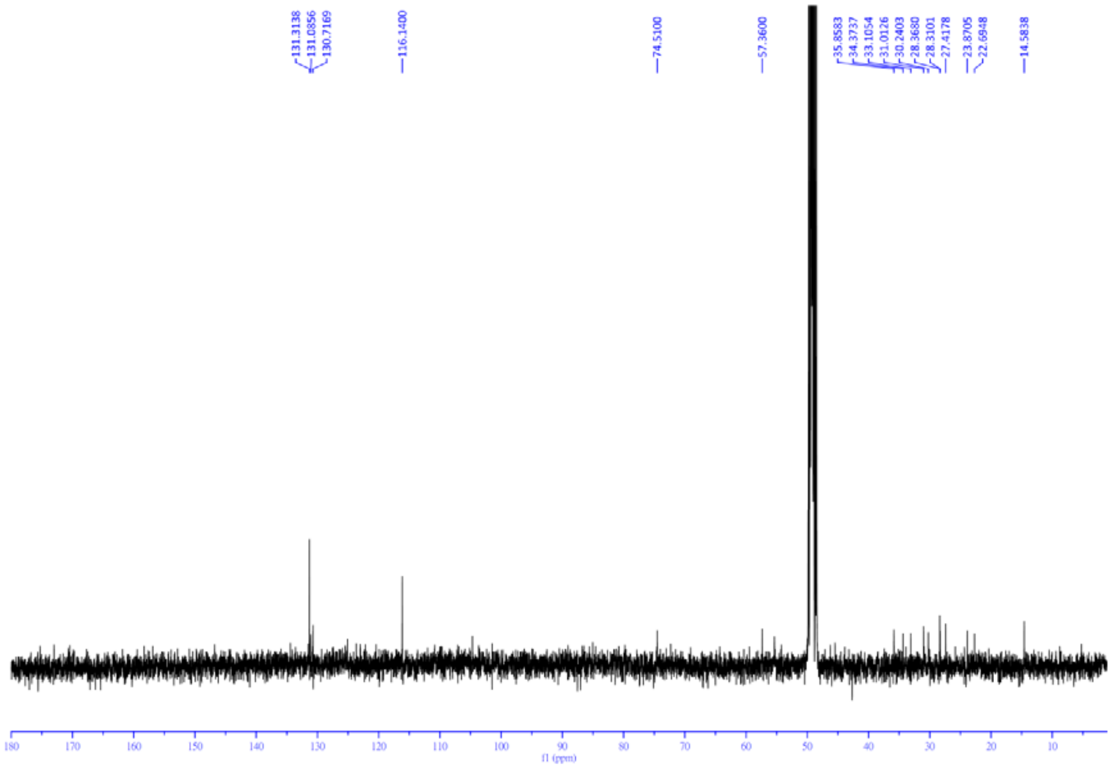

Figure S52. ^{13}C NMR spectrum of **6**

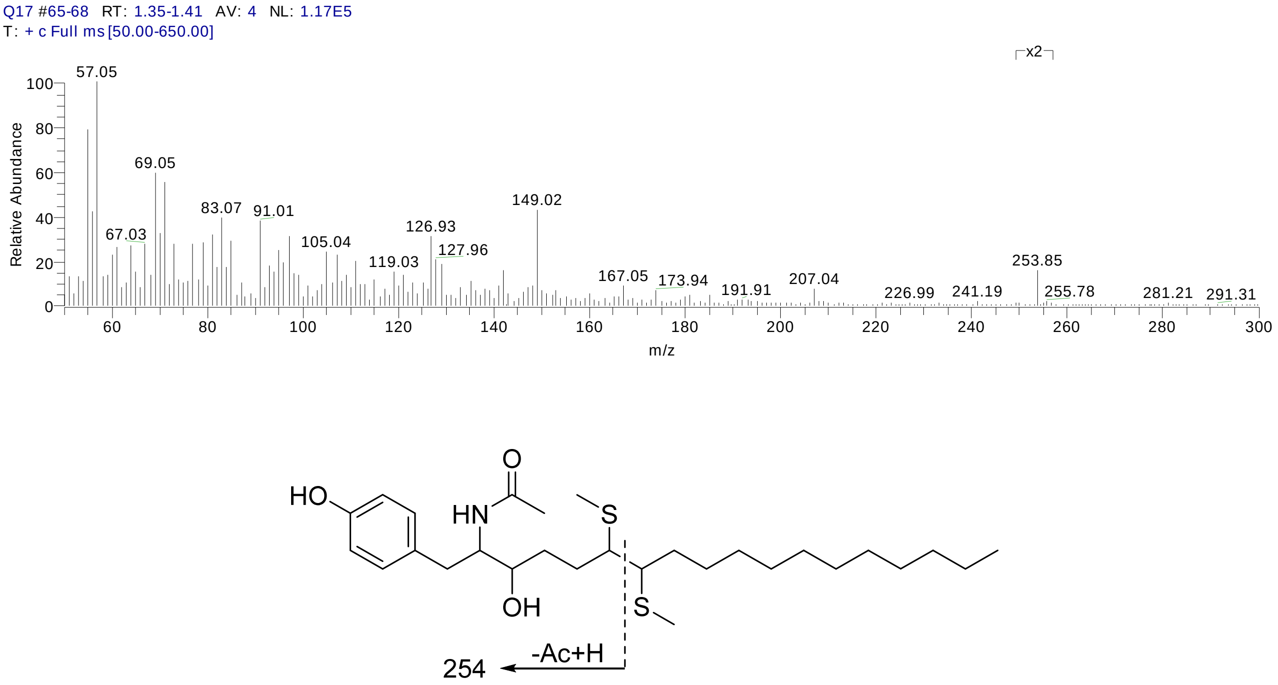

Figure S53. EIMS spectrum and fragmentation of the dimethyl disulfide derivative of **6**

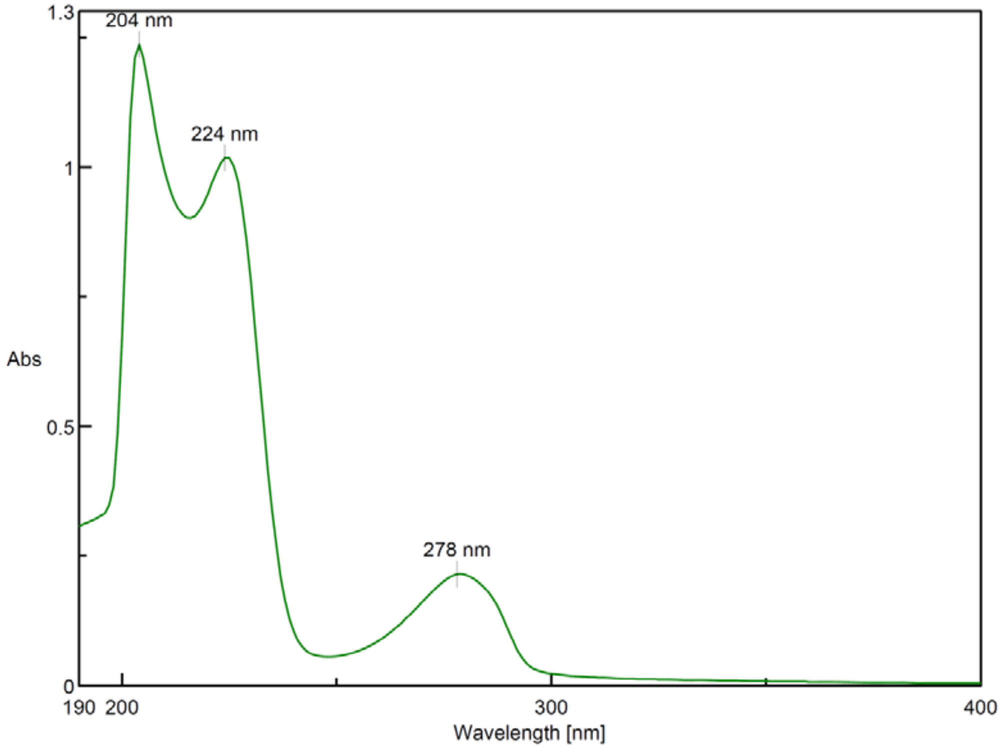

Figure S54. UV spectrum of **7**

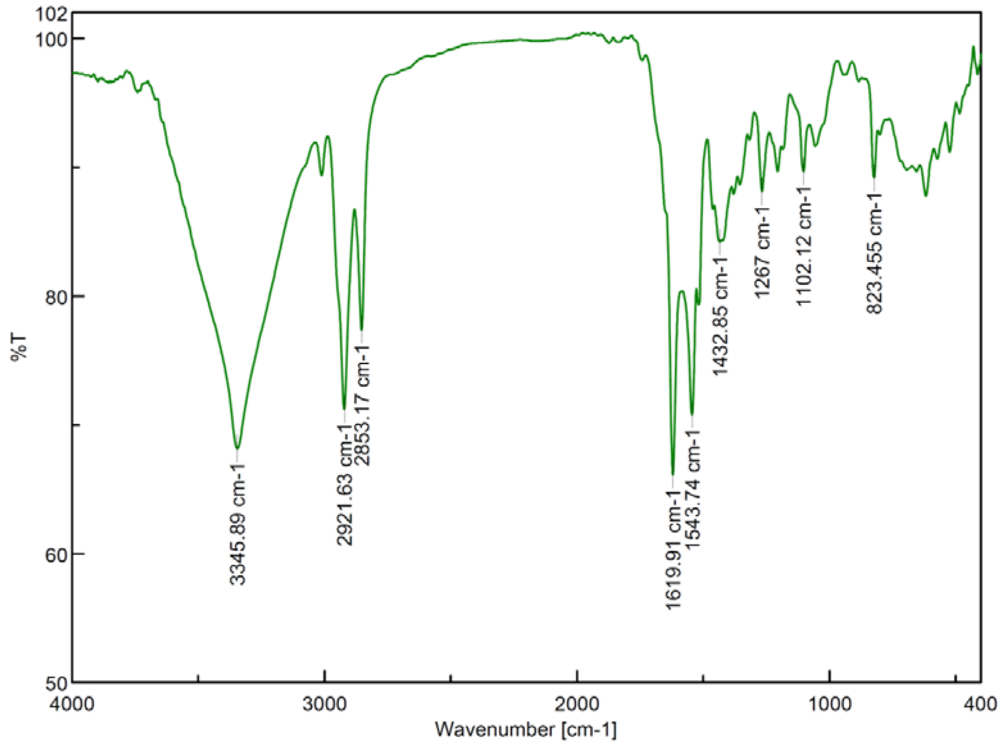

Figure S55. FTIR spectrum of **7**

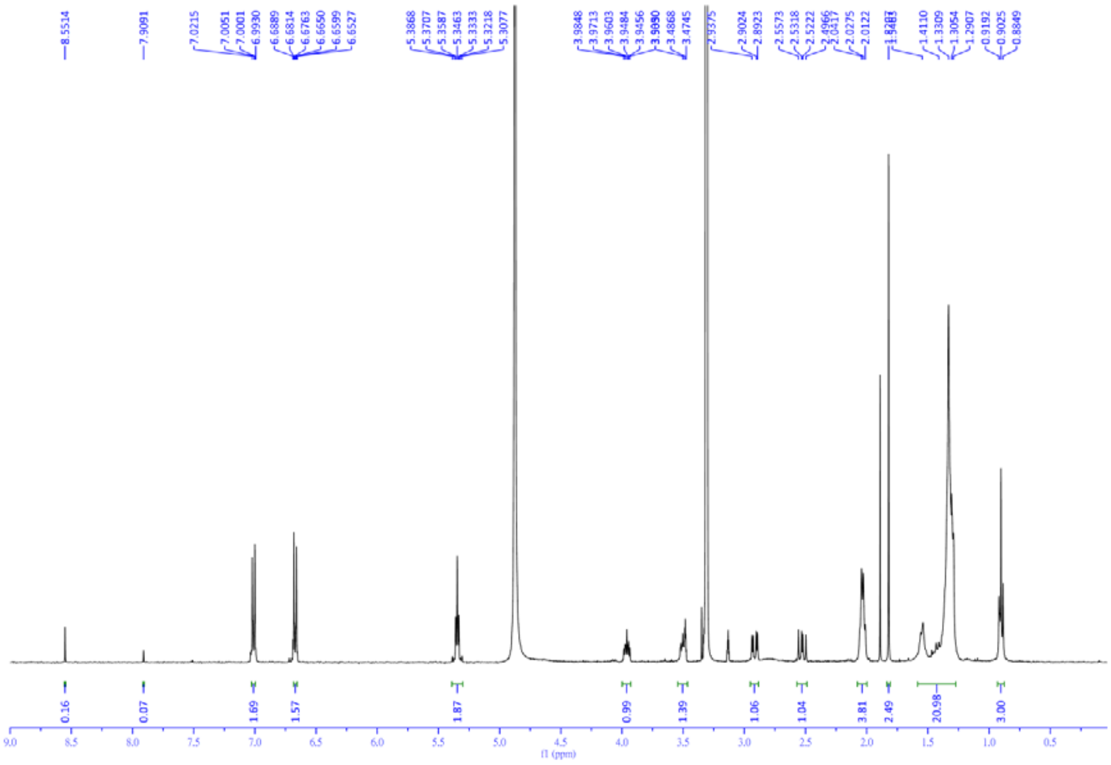

Figure S56. ^{1}H NMR spectrum of **7**

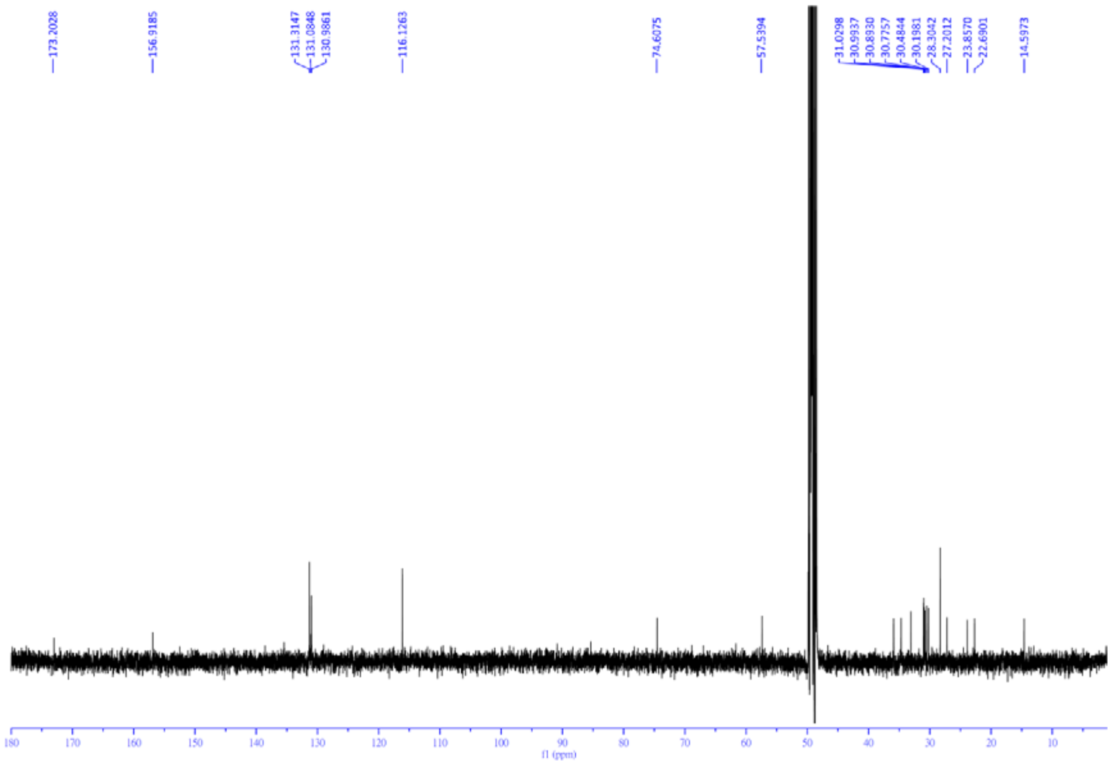

Figure S57. ^{13}C NMR spectrum of **7**

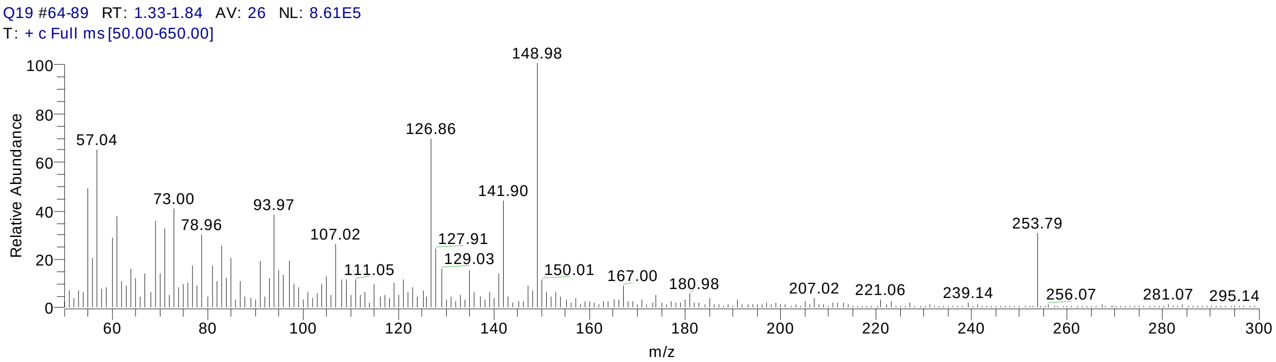

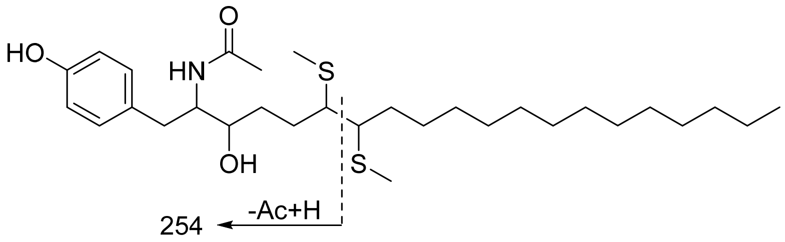

Figure S58. EIMS spectrum and fragmentation of the dimethyl disulfide derivative of **7**

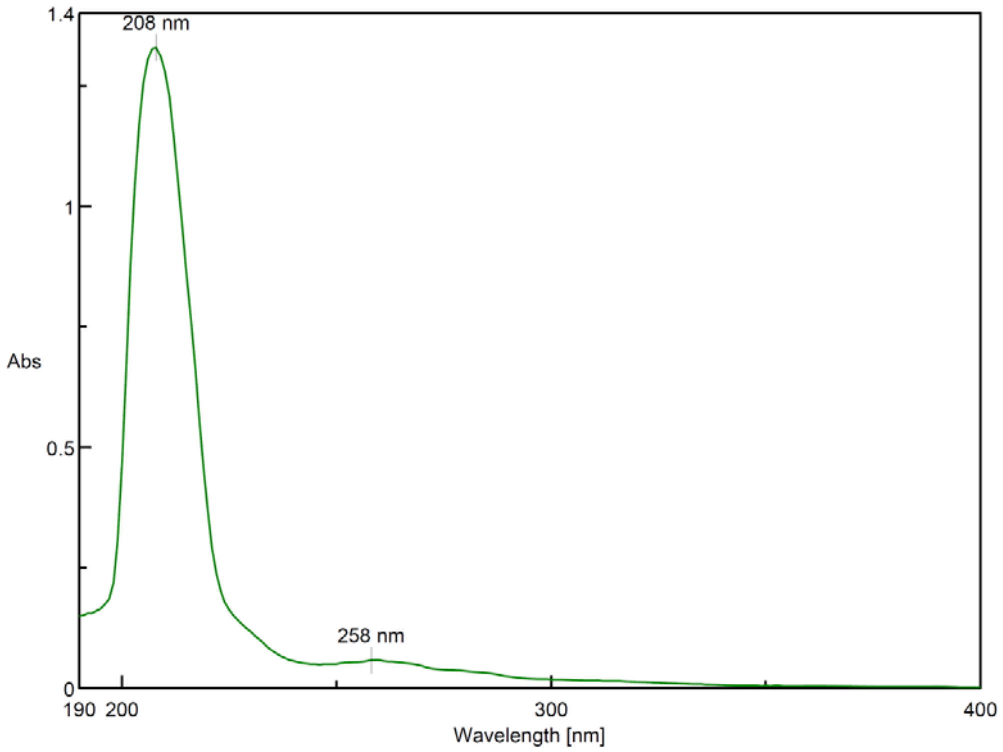

Figure S59. UV spectrum of **8**

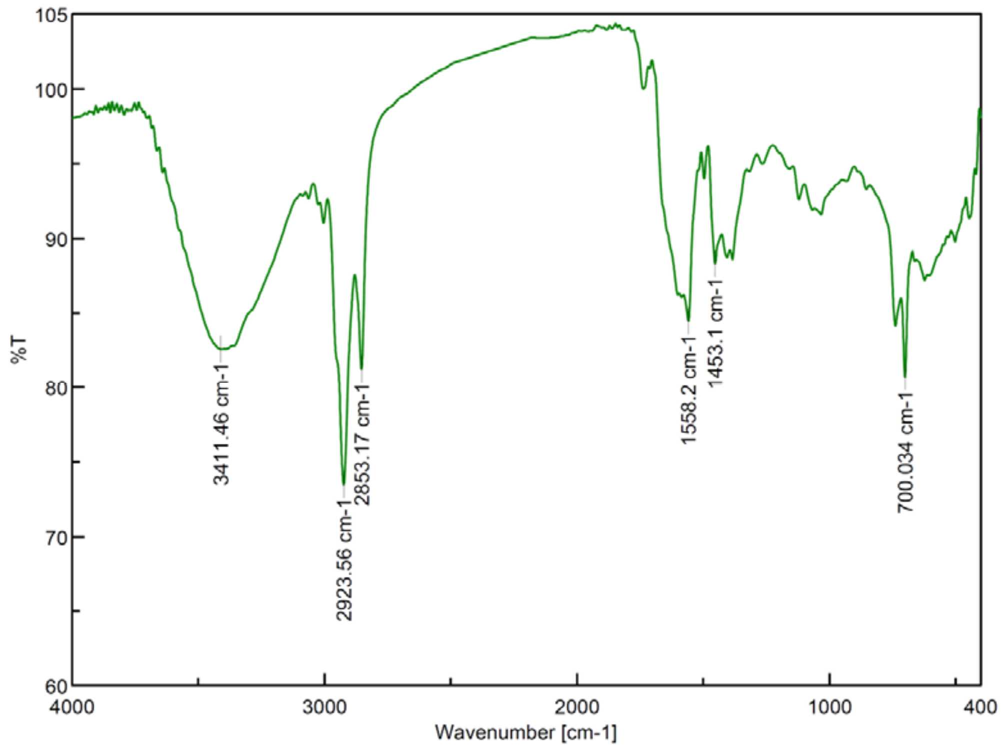

Figure S60. FTIR spectrum of **8**

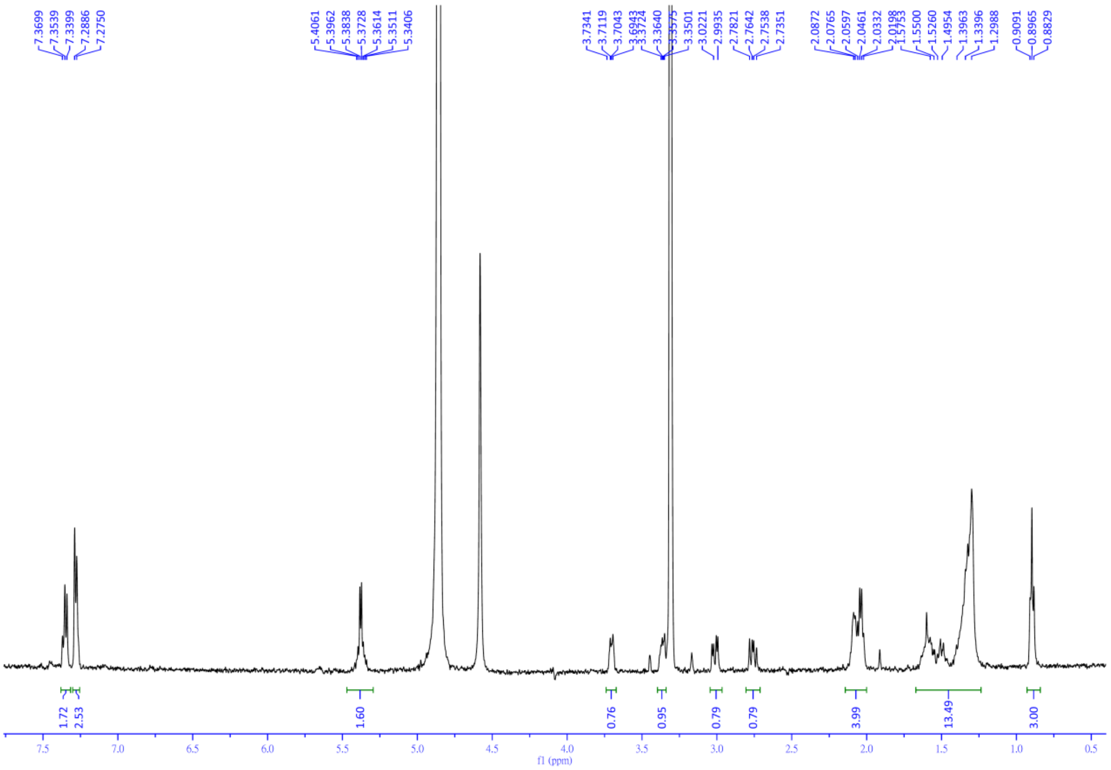

Figure S61. ^{1}H NMR spectrum of **8**

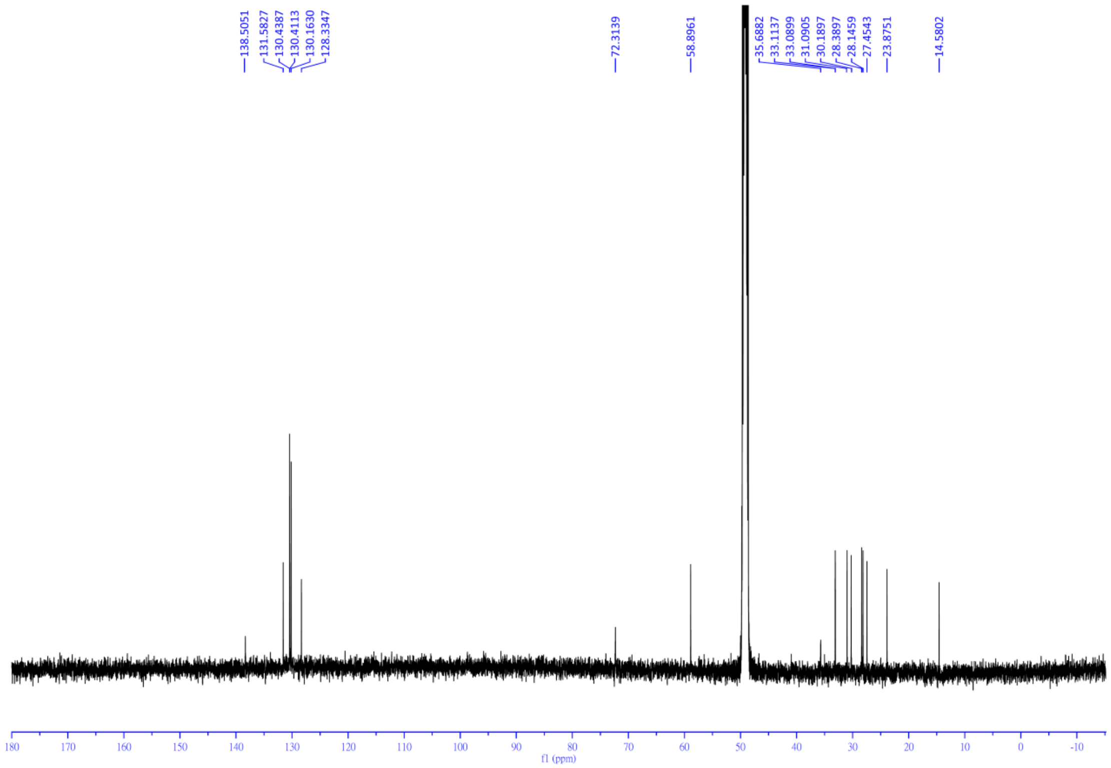

Figure S62. ^{13}C NMR spectrum of **8**

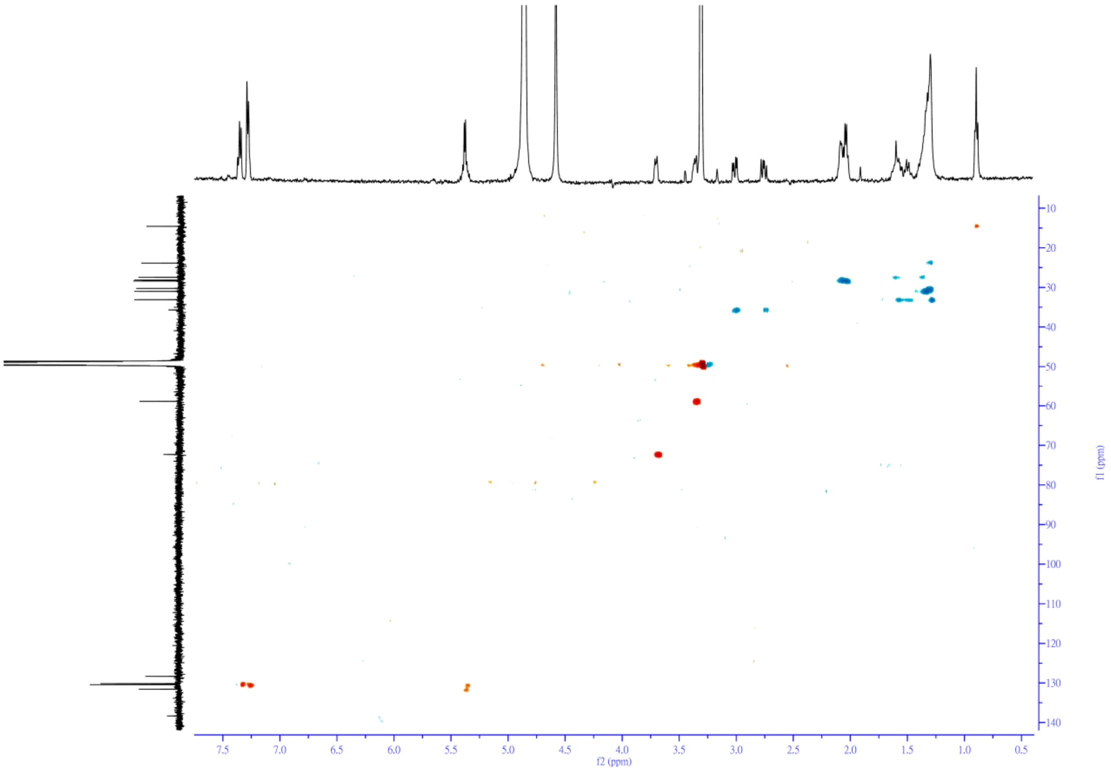

Figure S63. HSQC spectrum of **8**

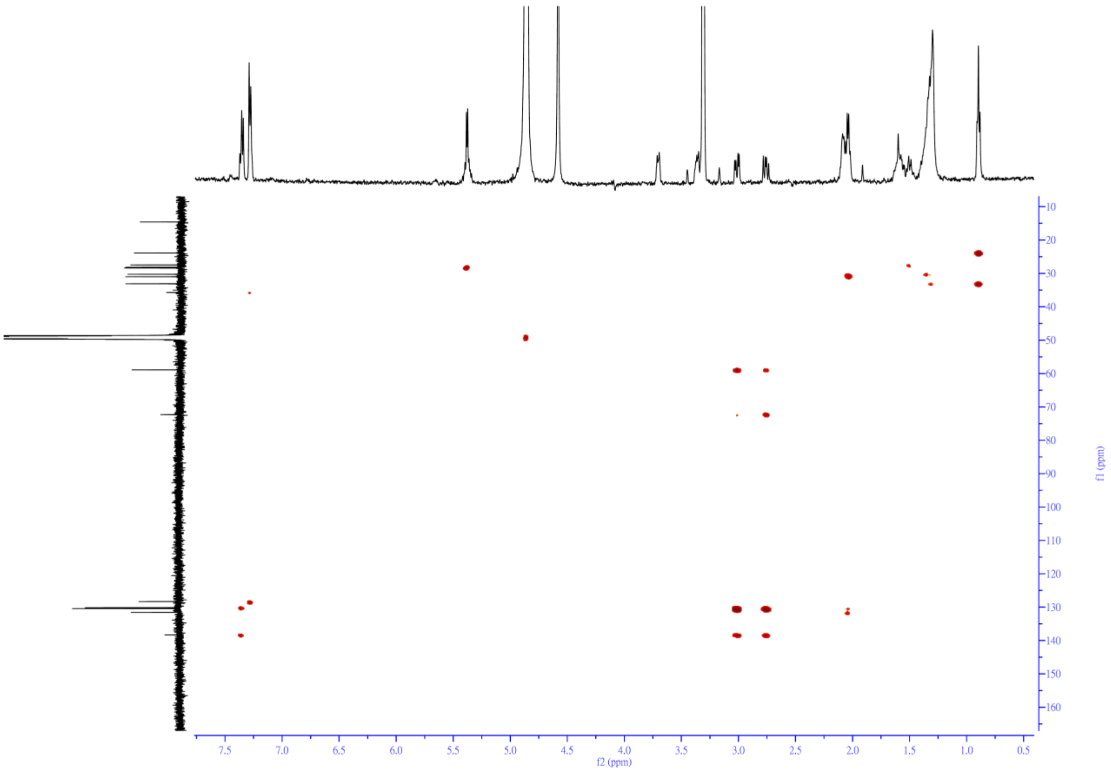

Figure S64. HMBC spectrum of **8**

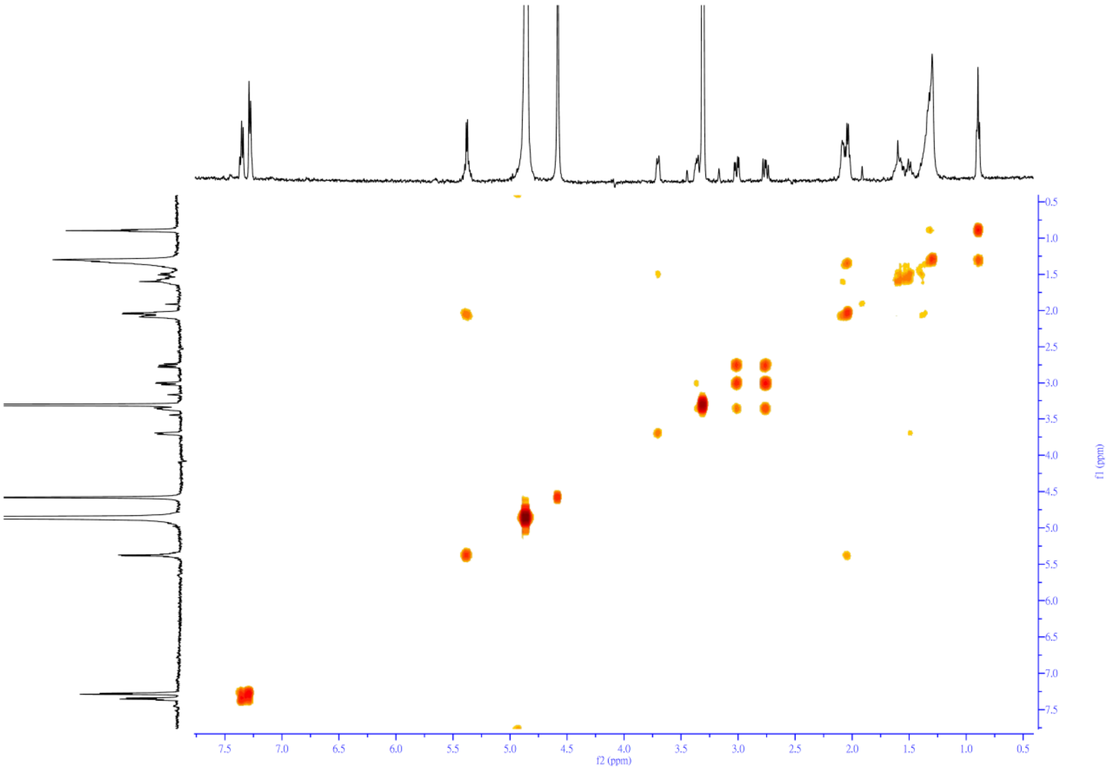

Figure S65. ^{1}H-^{1}H COSY spectrum of **8**

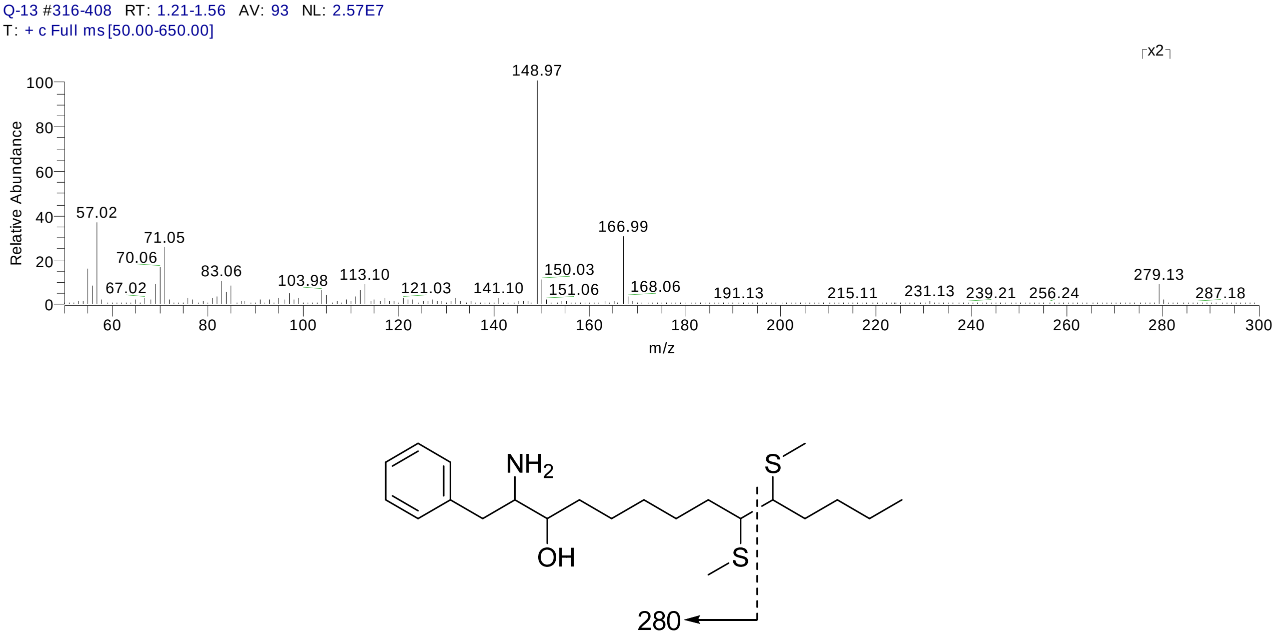

Figure S66. EIMS spectrum and fragmentation of the dimethyl disulfide derivative of **8**

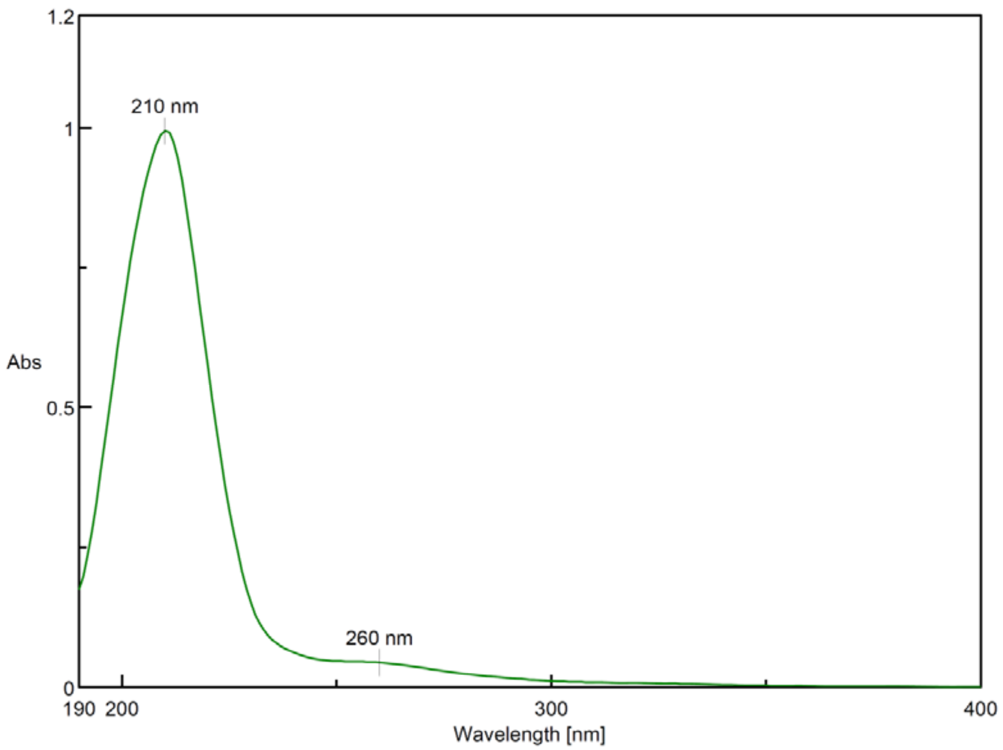

Figure S71. UV spectrum of **9**

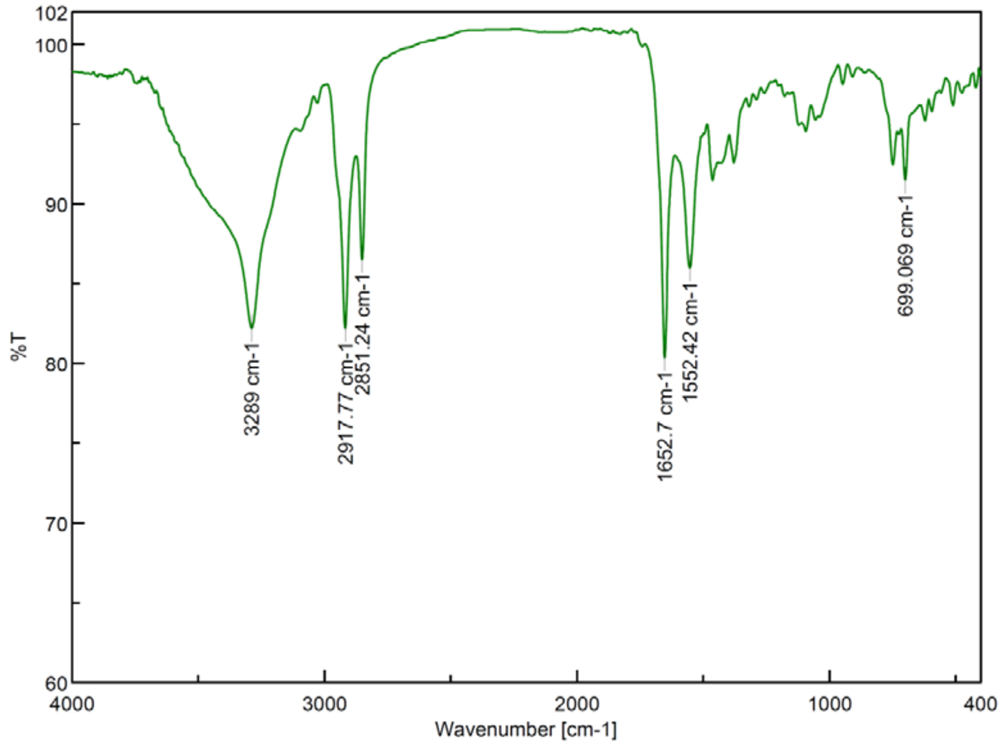

Figure S72. FTIR spectrum of **9**

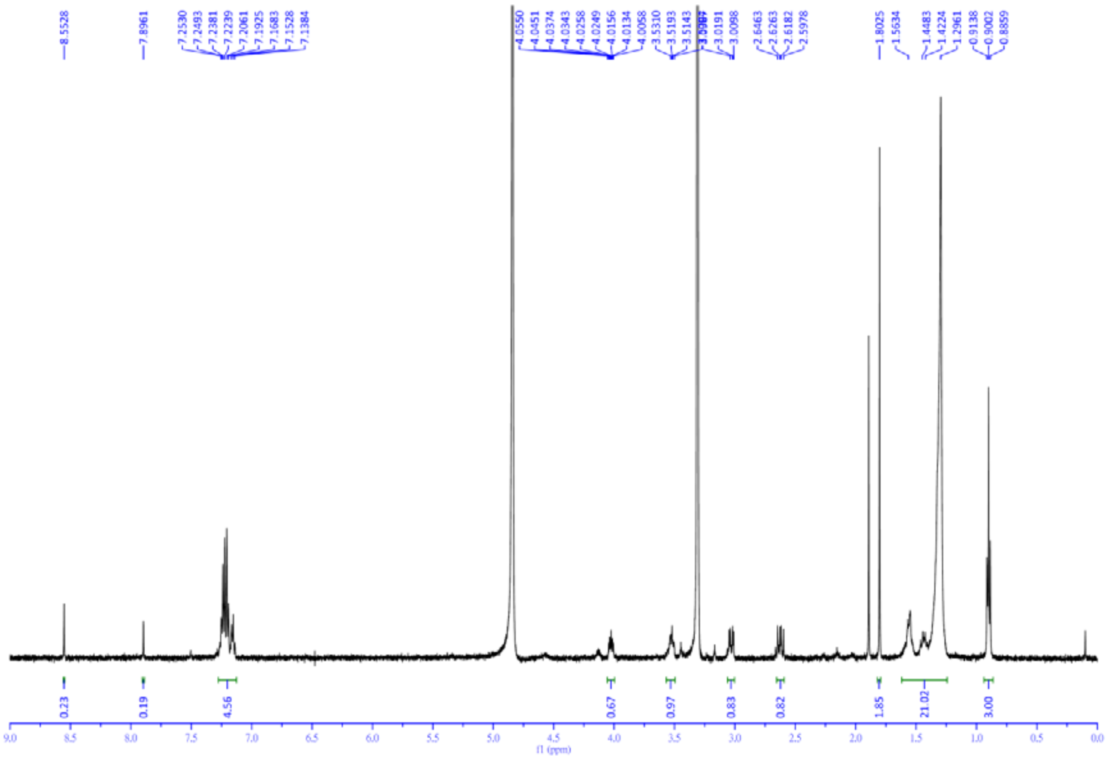

Figure S73. ^{1}H NMR spectrum of **9**

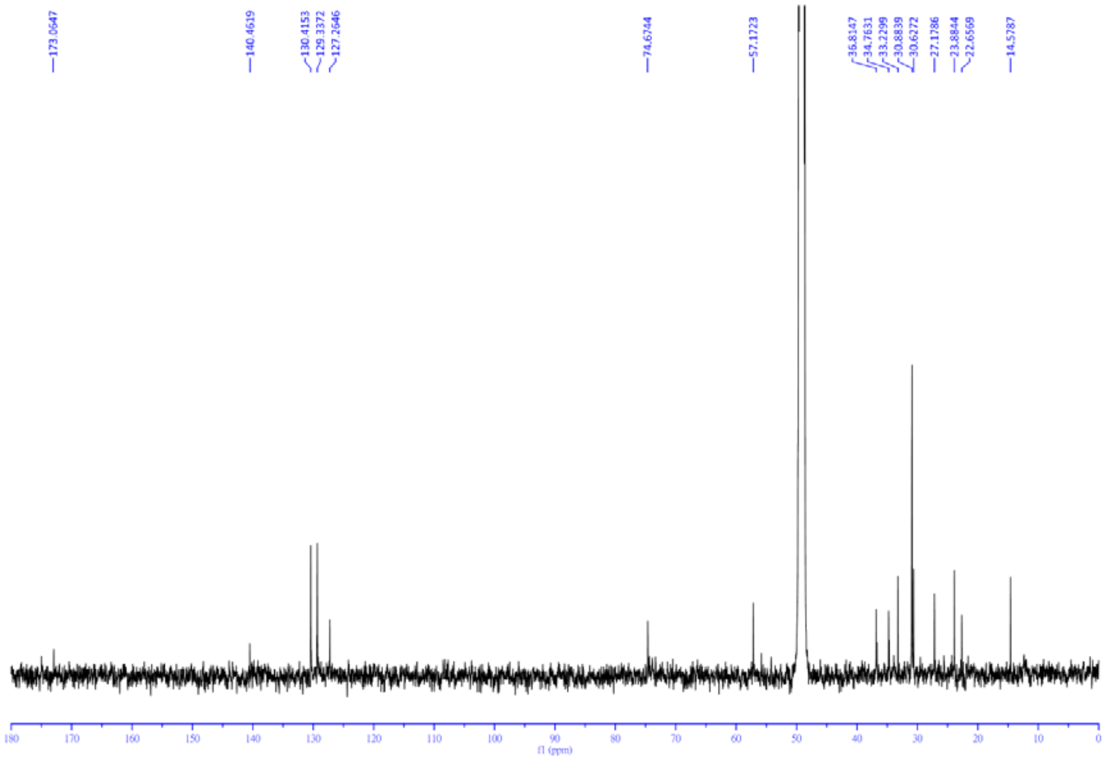

Figure S74. ^{13}C NMR spectrum of **9**

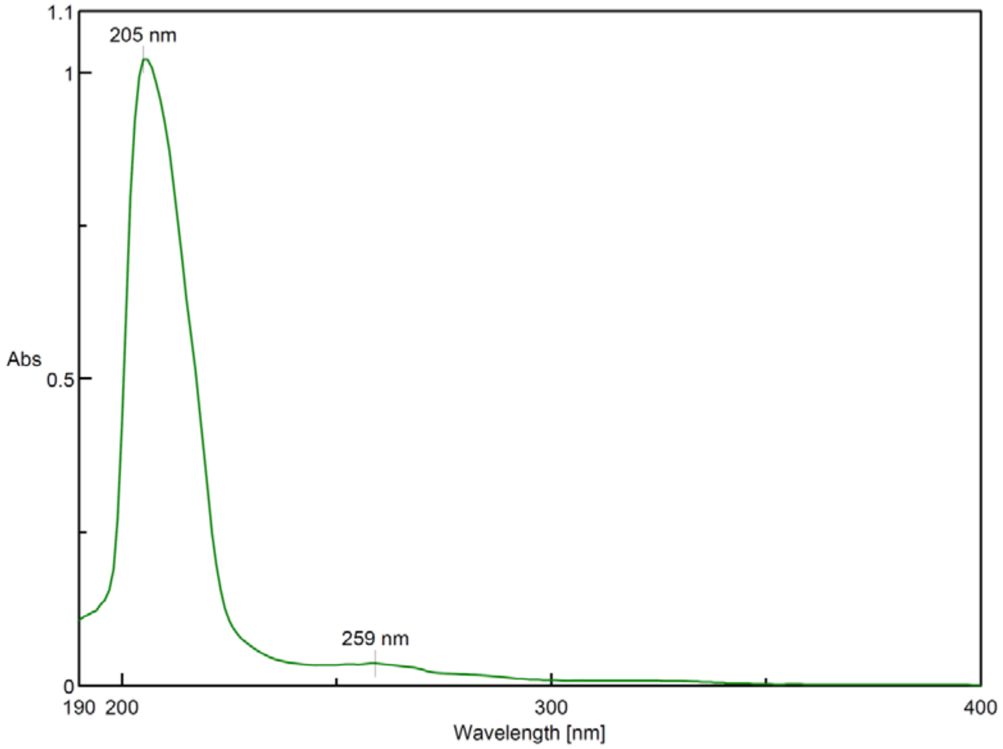

Figure S67. UV spectrum of **10**

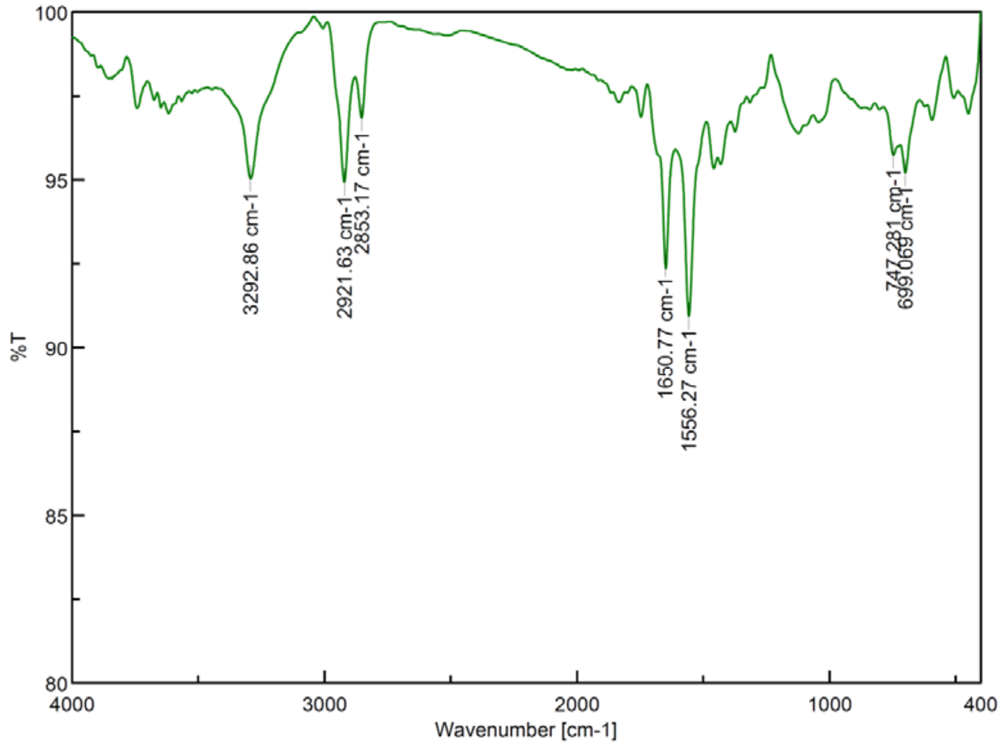

Figure S68. FTIR spectrum of **10**

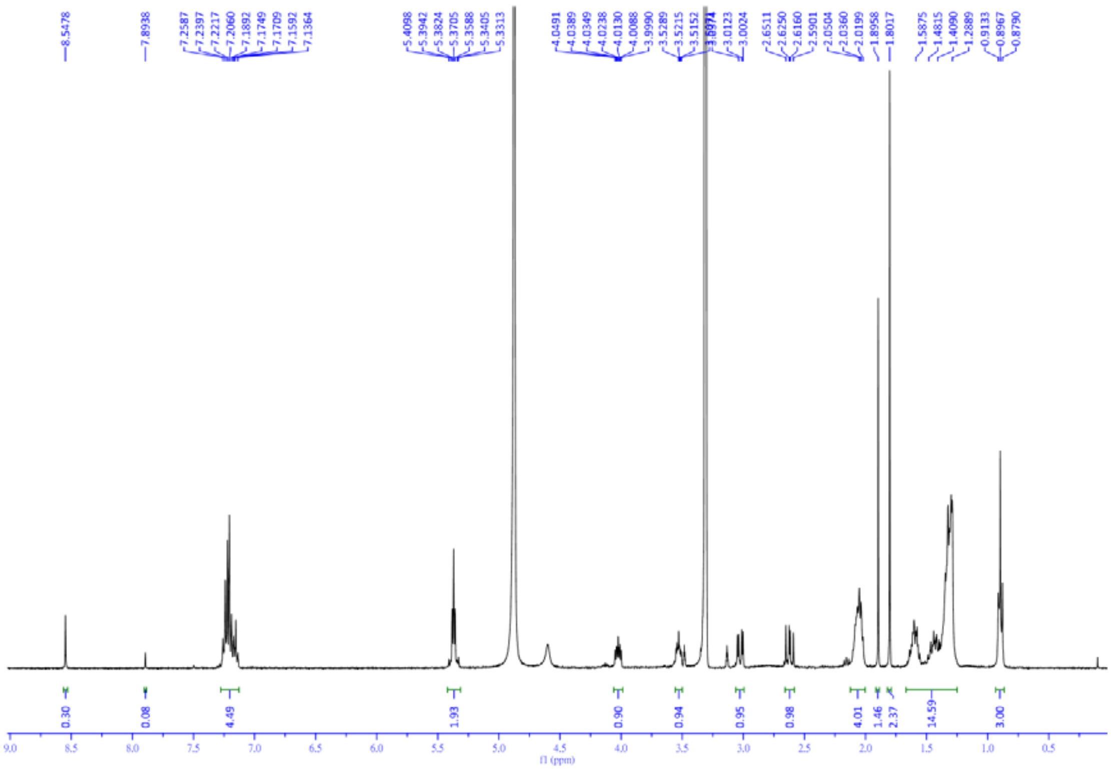

Figure S69. ^{1}H NMR spectrum of **10**

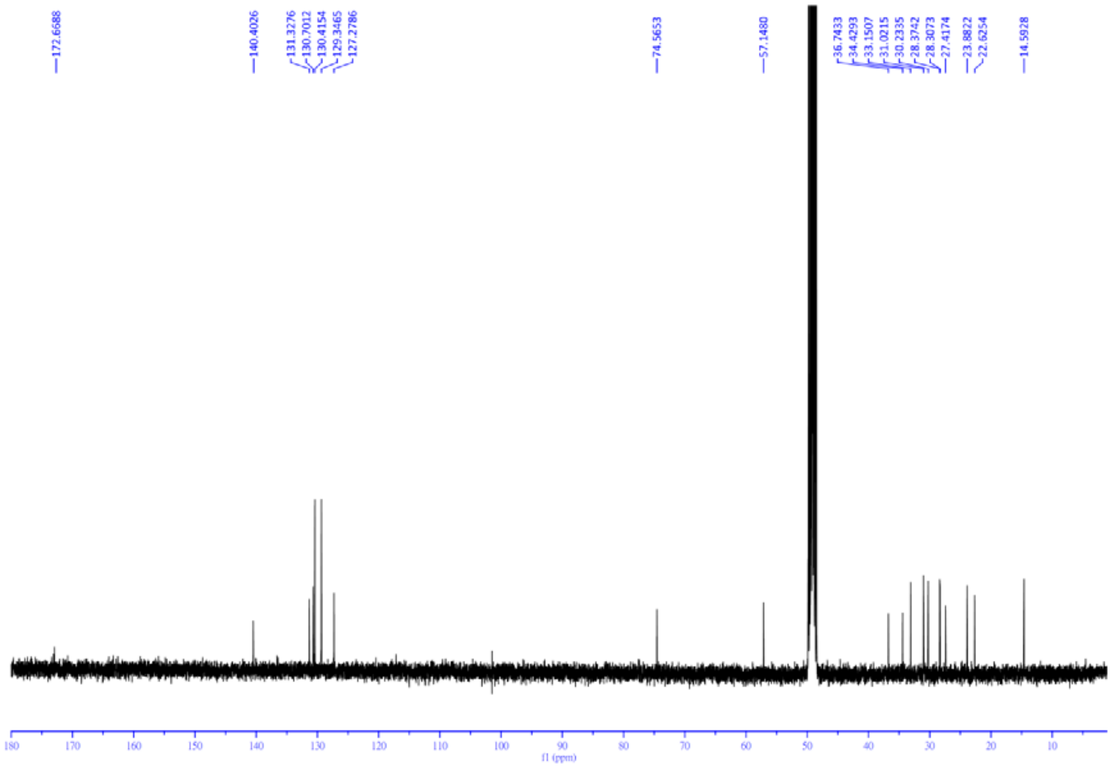

Figure S70. ^{13}C NMR spectrum of **10**

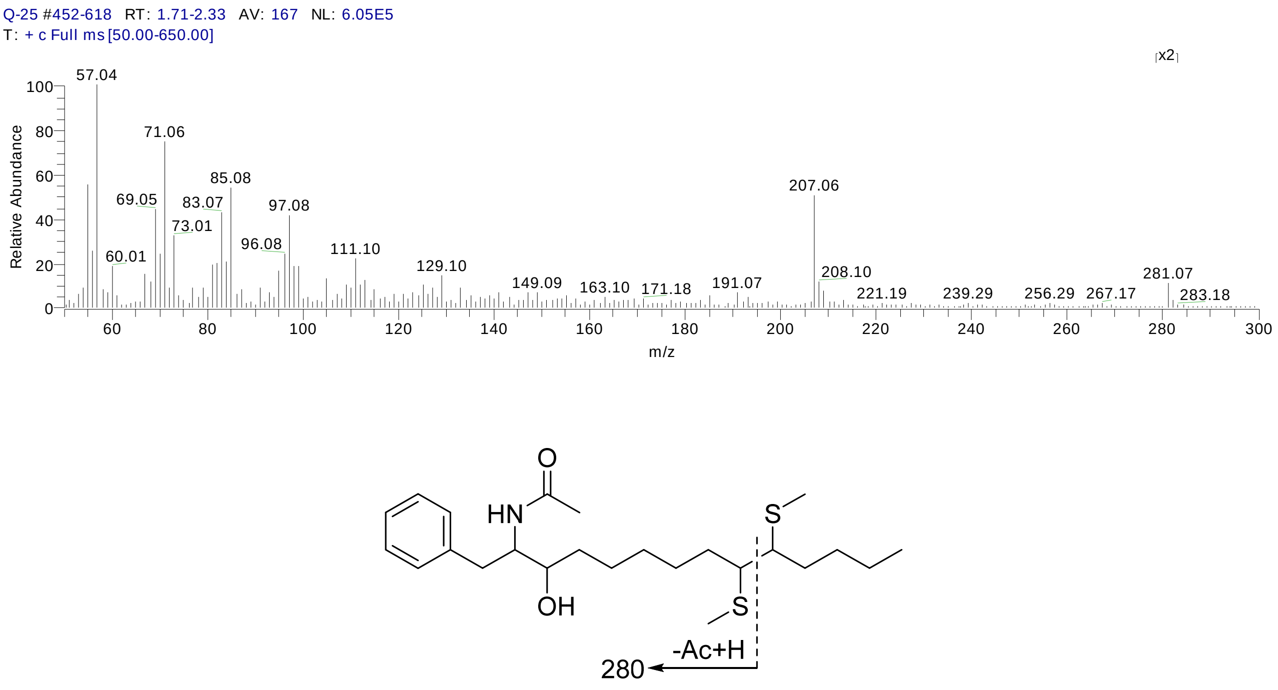

Figure S75. EIMS spectrum and fragmentation of the dimethyl disulfide derivative of **10**

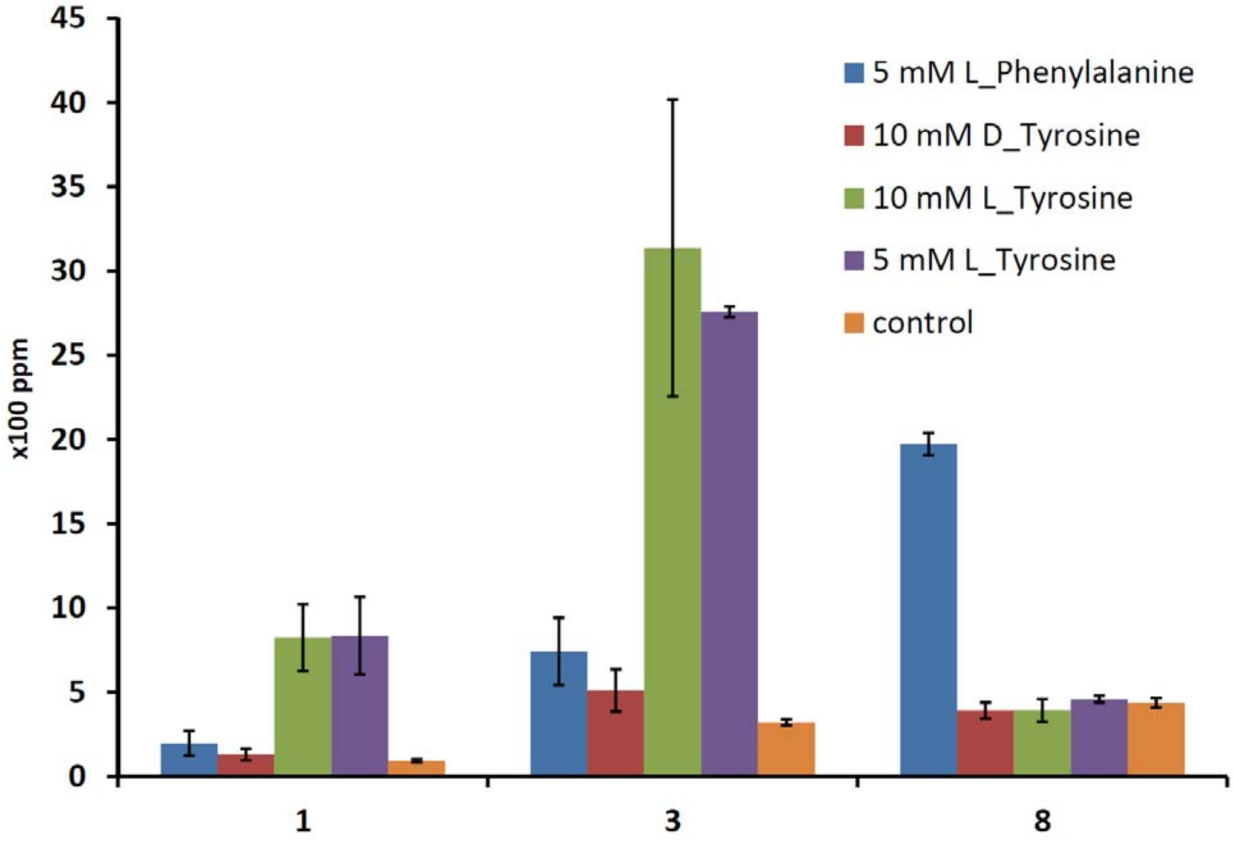

Figure S76. Quantitation results of **1**, **3**, and **8** from *Vibrio* sp. QWI-06 under different cultural condition. Control is marine agar medium and tyrosine and phenylalanine were added for evaluating the production of vitroprocines.

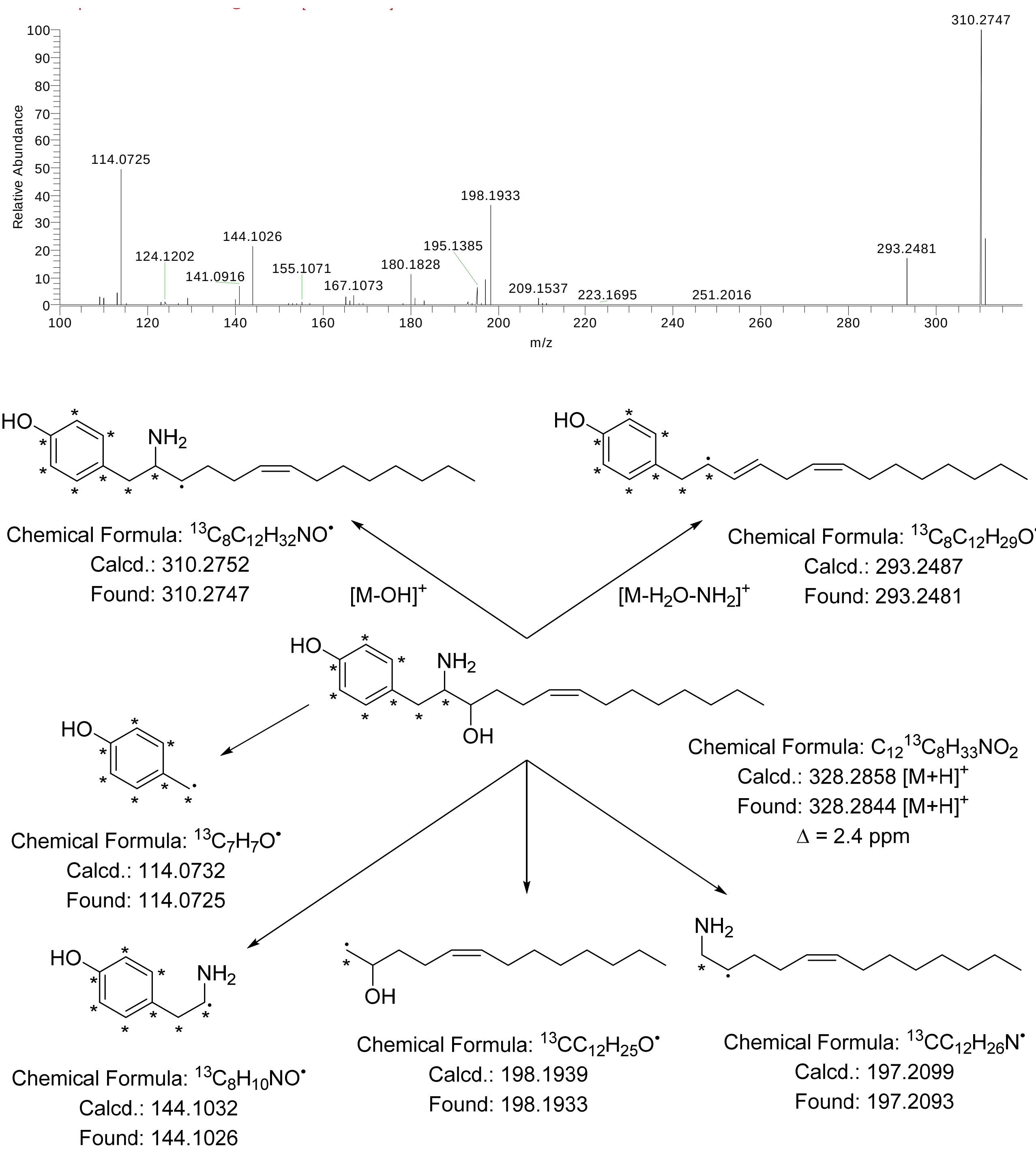

Figure S77. HR-ESIMS/MS spectrum and fragmentation of **1** from *Vibrio* sp. QWI-06 feed with $^{13}C_9$-tyrosine.

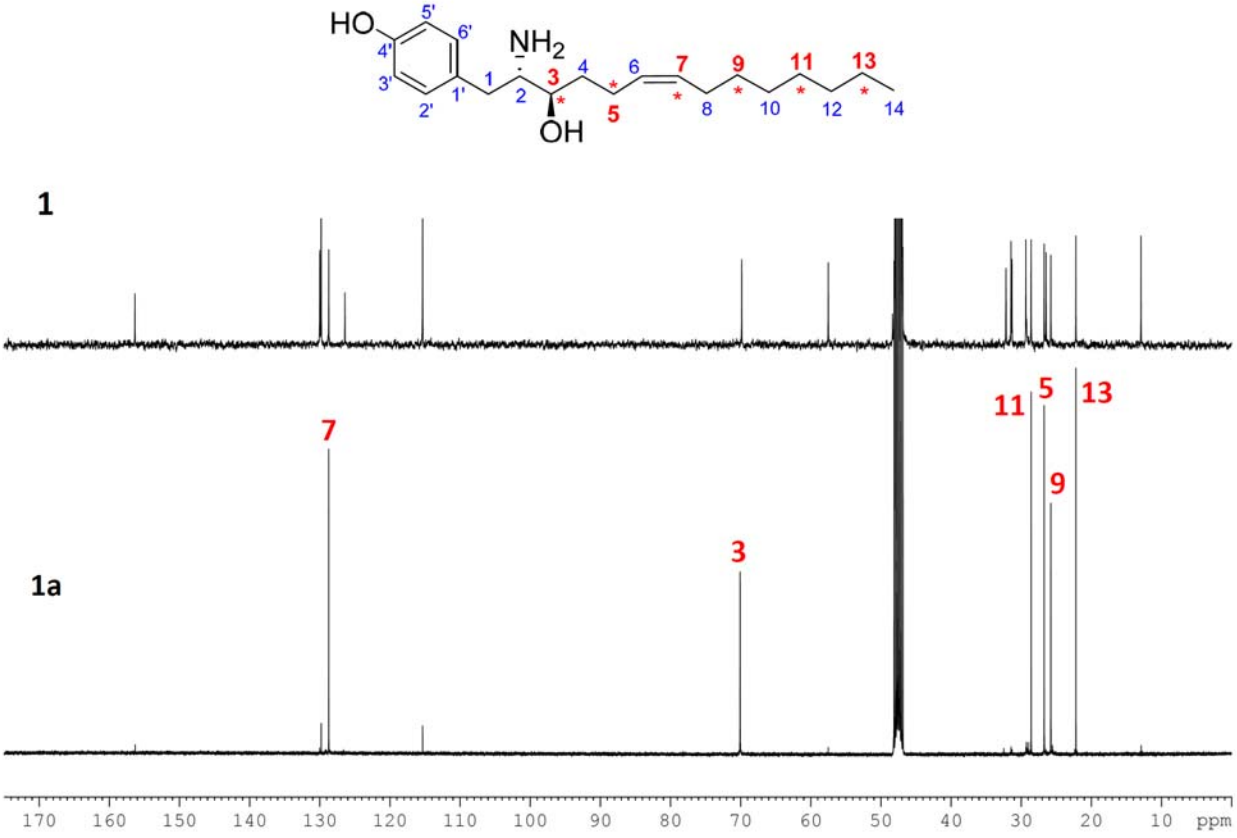

Figure S78. ^{13}C NMR spectra of **1** and **1a**. **1a** was isolated from *Vibrio* sp. QWI-06 feed with 1-^{13}C-sodium acetate. The enhanced carbon signals of **1a** were marked in red.
